# A Bioconjugate Vaccine Against Extra-Intestinal Pathogenic *Escherichia coli* (ExPEC)

**DOI:** 10.3390/vaccines13040362

**Published:** 2025-03-28

**Authors:** Linhui Hao, Wenhua Huang, Yan Guo, Xiankai Liu, Jun Wu, Li Zhu, Chao Pan, Hengliang Wang

**Affiliations:** 1 Laboratory of Advanced Biotechnology, Beijing Institute of Biotechnology, Beijing 100071, China; 15305168327@163.com (L.H.); yanguobrilliant@163.com (Y.G.); liuxk007@163.com (X.L.); wujun@bmi.ac.cn (J.W.); jewly54@bmi.ac.cn (L.Z.); 2 State Key Laboratory of Pathogen and Biosecurity, Academy of Military Medical Sciences, Beijing 100071, China; huangwh1993@163.com

**Keywords:** extra-intestinal pathogenic *Escherichia coli* (ExPEC), bioconjugate vaccine, protein glycan coupling technology (PGCT), cholera toxin B subunit (CTB)

## Abstract

**Background**: Extra-intestinal pathogenic *Escherichia coli* (ExPEC) represents a major global public health challenge due to its ability to cause diverse clinical infections, including urinary tract infections, bacteremia, neonatal meningitis, and sepsis. The growing prevalence of multidrug-resistant (MDR) ExPEC strains, which rapidly erode antibiotic efficacy, underscores vaccine development as a critical priority. Bioconjugate vaccines have emerged as a promising approach to mitigate ExPEC-associated infections. **Methods and Results**: In this study, we utilized protein glycan coupling technology (PGCT) based on oligosaccharyltransferase (OST) PglL to engineer a tetravalent bioconjugate vaccine targeting four predominant ExPEC serotypes (O1, O2, O6, and O25). We conducted a series of experiments to demonstrate the efficacy of the conjugate vaccine in eliciting humoral immune responses and inducing the production of specific antibodies against *Escherichia coli* O1, O2, O6, or O25 serotypes. **Conclusions**: This work establishes the first application of the *O*-linked PGCT system for engineering bioconjugate vaccines against ExPEC infections.

## 1. Introduction

Extraintestinal pathogenic *Escherichia coli* (ExPEC) poses a major global public health challenge due to its capacity to induce diverse infections, including urinary tract infections, bacteremia, neonatal meningitis, and sepsis [1]. Distinct from commensal *E. coli* or intestinal pathogenic strains, ExPEC establishes extraintestinal infections by employing virulence factors such as adhesins, toxins, iron-uptake systems, and capsular polysaccharides that mediate tissue colonization [2,3]. The progressive emergence of multidrug-resistant ExPEC lineages carrying extended-spectrum β-lactamases [4], carbapenemases (e.g., NDM-1) [5], and plasmid-mediated AmpC β-lactamases (e.g., CMY) [6], is rapidly eroding the efficacy of antibiotics. Vaccine development has emerged as a high priority to safeguard global antimicrobial stewardship efforts [7].

The O-antigen polysaccharide (OPS), a component of bacterial surface lipopolysaccharide (LPS), has been identified as a promising target for bacterial vaccine development [8]. *E. coli* can be classified into 181 serotypes based on O-antigen variability, yet only a limited subset demonstrates pathogenic potential [9,10]. Epidemiological data from 2011 to 2017 have indicated that O25, O2, O6, and O1 serotypes constitute the predominant epidemic strains of ExPEC [11]. In 2014, the Janssen company developed a bioconjugate vaccine targeting these four serotypes and subsequently advanced it into clinical trials [12]. The phase 1 clinical trial demonstrated that this tetravalent ExPEC bioconjugate candidate vaccine exhibited favorable safety and elicited robust antibody responses against vaccine-targeted serotypes.

Current antibacterial vaccine strategies encompass live-attenuated, inactivated whole-cell, subunit, and polysaccharide-based formulations [13]. Among these, bioconjugate vaccines are formed by covalently conjugating antigenic polysaccharides to carrier proteins. As the resulting glycoconjugates act as T-cell-dependent antigens [14], they engage T cells during immunization, activate immunological memory, and induce antibody isotype switching to complement-activating subtypes such as IgG1 [15]. These vaccines exhibit significantly higher antibody affinity than traditional polysaccharide vaccines. Bioconjugate vaccines represent a highly promising vaccine platform, as they overcome the limitations of traditional polysaccharide vaccines that are classified as T-cell-independent antigens [16,17]. This platform has been successfully implemented in licensed vaccines against *Streptococcus pneumoniae* (e.g., PREVNAR^®^ 13) [18], demonstrating clinical efficacy against invasive bacterial diseases.

With the breakthroughs in elucidating prokaryotic protein glycosylation mechanisms and their practical applications, the development of bioconjugate vaccines based on protein glycan coupling technology (PGCT) has emerged as a prominent focus in vaccine research [19,20,21,22]. This innovative methodology utilizes bacterial oligosaccharyltransferases (OSTs) to mediate OPS transfer from lipid carriers to carrier proteins [23]. In OSTs research, the OST PglB of *Campylobacter jejuni* represents the first identified *N*-linked glycosylation enzyme, successfully achieving glycosylation of its natural substrate PEB3 and AcrA within the *E. coli* expression system [24]. Our team previously developed a bioconjugate vaccine platform leveraging the OST PglL of *Neisseria meningitidis* [25]. The OST PglS of *Acinetobacter* species has recently broadened bioconjugate vaccine development through enhanced technical adaptability [20]. Our team previously developed another bioconjugate vaccine platform utilizing the OST PglL of *Neisseria meningitidis*. Compared to PglB, PglL exhibits lower substrate specificity [26], enabling it to recognize a wider range of pathogen polysaccharides, including those from *Shigella*, *S. Paratyphi, Klebsiella pneumoniae* and *Yersinia enterocolitica* [22,27,28,29]. Notably, for *Salmonella* paratyphoid A OPS, which cannot be recognized by PglB, a conjugate vaccine has been successfully prepared using the PglL system [27]. Furthermore, by establishing engineered *E. coli* host cells, we have achieved a safer and more efficient method for preparing conjugate vaccines. Recently, the OST PglS of *Acinetobacter* species has further expanded the possibilities for bioconjugate vaccine development through enhanced technical adaptability.

Given the serious threat posed by ExPEC to human health, we aim to advance the development of a multivalent ExPEC vaccine based on our platform.

The application of genetically engineered *E. coli* as a microbial production platform offers a biosafe strategy for developing bioconjugate vaccines against highly pathogenic strains [30]. In this study, experiments were conducted using the engineered *E. coli* WdlO-d01 constructed in our laboratory as the host cell. This recombinant strain underwent dual knockout of the *waaL* gene and *wbbH-L* gene cluster through CRISPR-Cas9 gene editing technology [29]. The deletion of the *waaL* gene, which regulates the coupling of polysaccharides with the lipid A core, eliminates competition with glycosyltransferases. Additionally, the removal of its own polysaccharide gene cluster *wbbH-L* prevents the interference of its enzymes in the synthesis of exogenous polysaccharides.

## 2. Materials & Methods

### 2.1. Bacterial Strains, Plasmids, Primers, and Growth Conditions

The bacterial strains and plasmids used in this study are listed in Table S1, and the primers are listed in Table S2. All bacterial strains were cultured in Luria–Bertani (LB) liquid medium or on LB agar (1.5%) at 37 °C. For protein expression, strains carrying plasmids were grown at 37 °C until the optical density at 600 nm reached 0.6. Protein expression was induced by adding 0.2 mM isopropyl-β-D-thiogalactopyranoside (IPTG), followed by 12 h incubation at 30 °C.

### 2.2. Plasmid Construction

The target DNA fragments were amplified through long-range PCR polymerase (Takara, Japan) and recombined into the plasmid vector using a homologous recombination system (Vazyme, Nanjing, China). The recombinant plasmid was subsequently transformed into *E. coli* NEB10-beta competent cells (Biomed, Beijing, China) for amplification and sequence verification.

### 2.3. Experimental Animals

Six-week-old female BALB/c mice (specific pathogen-free) were procured from Beijing SiPeiFu Biotechnology Company (Beijing, China). All animal experiments were conducted in accordance with the institutional guidelines established by the Institutional Animal Care and Use Committee at Beijing Institute of Biotechnology (Approval No. IACUC-DWZX-2023-059, obtained on 29 December 2023).

### 2.4. LPS and OPS Extraction

The *E. coli* O1, O2, O6, and O25 were cultured in an Erlenmeyer flask at 37 °C with shaking (200 rpm) for 12 h. Cells were collected via centrifugation (8000 rpm, 15 min), followed by supernatant removal. The pellet underwent three washing cycles with ice-cold PBS, culminating in a final brief centrifugation (3 min) for complete supernatant removal. Cells were resuspended in ddH_2_O (10 mL/g wet biomass) and subjected to three thermal-cycling treatments alternating between ice bath incubation (3 min) and 68 °C water immersion (3 min). An equal volume of 90% phenol solution was added, and the mixture was vigorously shaken at 68 °C for 30 min. After centrifugation at 8000 rpm for 30 min, the aqueous phase was collected. This extraction step was repeated with an equal volume of ddH_2_O, and the combined aqueous phases were transferred into pre-boiled dialysis tubing (3500 Da molecular weight cutoff). The sample was dialyzed against ddH₂O for 3 days with water changes every 12 h.

The dialysate was sequentially treated with DNase and RNase (1:1000 *v*/*v*) at 37 °C for 3 h, followed by Proteinase K (final concentration 100 μg/mL) at 60 °C for 1 h. The solution was then boiled for 10 min and centrifuged at 8000 rpm for 20 min to obtain LPS. For OPS purification, glacial acetic acid was added to solution to a final concentration of 1% (*v*/*v*). After boiling for 90 min and adjusting the pH to 7.0 with NaOH, the sample was centrifuged at 19,700 rpm for 5 h. The supernatant containing purified OPS was collected for further analysis.

### 2.5. Purification of Target Glycoproteins

Initially, 1 L of induced bacterial solution was centrifuged at 6000× *g* for 15 min to collect the bacterial cells. Subsequently, 40 mL of equilibration buffer A1 was added per 1 L of bacterial solution to resuspend the cells, and a high-pressure homogenizer was utilized for cell disruption, with the process repeated three times. After completion, the supernatant was collected by centrifuging at 8000× *g* for 30 min. In the Ni-NTA column (Roche, Germany) purification process, the column was first cleaned by sequential rinsing with ddH2O, 0.5 M NaOH, and ddH2O until both the ion concentration and UV absorption peak stabilized. Following this, the nickel column was equilibrated using equilibration buffer A1. The supernatant obtained from the previous step was then loaded onto the column at a flow rate of 4 mL/min. After sample loading, the column was further rinsed with equilibration buffer A1 until a stable A280 UV absorption value was achieved, thereby removing unbound proteins. Next, elution buffer B1 was used for elution, and the A280 UV absorption was monitored. When the value began to rise, the eluate was collected in 10 mL tubes. Finally, the column was cleaned by sequential rinsing with water, NaOH, and water again, treated with 20% ethanol, and stored at 4 °C. The pooled eluate was concentrated using a 30 kDa ultrafiltration centrifugal device (Merck, Kenilworth, NJ, USA) to a final volume of 1 mL. Preparation before sample loading involved sequential cleaning of the Sephadex 200 column (GE Healthcare, Chicago, IL, USA) with water, NaOH, and water again, followed by equilibration with PBS. Sample loading was then initiated by restarting the system, adjusting the injection valve to the loading mode, setting the flow rate to 1.5 mL/min, and maintaining the pressure at 4 MPa. During the collection phase, the glycoprotein started to emerge at approximately 10 min, with 1.5 mL collected in each tube.

### 2.6. Western Blot Analysis

We took 10 μL of the validated sample and added it to the sample loading well of a 4~20% SDS-PAGE precast gel (Genscript, Nanjing, China) for electrophoretic analysis. After electrophoresis was complete, we used the eBlot L1 (Genscript, China) rapid wet transfer device for membrane transfer. Following this, we immersed the PVDF membrane in Western blot blocking solution for 2 h, then washed the membrane twice with 1 × TBST for 7 min each time. Next, we incubated the PVDF membrane in antibody solution at room temperature for 1 h, followed by washing the membrane three times with 1 × TBST for 7 min each. If a secondary antibody was required, we incubated the PVDF membrane in goat anti-rabbit IgG antibody at room temperature for 20 to 30 min, and then we washed the membrane three times with TBST solution for 7 min each. Finally, we prepared ECL enhanced chemiluminescence substrate (CWBIO, Taizhou, China), incubated it on the membrane surface and detected using the ChemiDoc imaging system (Bio-Rad, San Francisco, CA, USA).

Horseradish peroxidase (HRP)-conjugated anti-6 × His antibody (CWBIO, Taizhou, China) (1:1500) was used to detect 6 × His-tag-fused proteins. *E. coli* O1, O6, and O25 antibodies (Nisseiken, Yamanashi, Japan) were utilized to detect LPS and glycoproteins. The antibody against *E. coli* O2 was produced by immunizing rabbits with whole *E. coli* O2 cells and subsequently adsorbing it with *E. coli* W3110 cell lysates. HRP-conjugated anti-rabbit IgG (Biodragon, Beijing, China) (1:15,000) was used as a secondary antibody.

### 2.7. Agglutination Assay

The bacterial test strain was washed three times with sterile saline and centrifuged at 8000 rpm for 5 min. The sediment was resuspended to a final concentration of approximately 1.0 × 10⁸ CFU/mL. Clean glass slides were divided into two areas: Zone A was prepared by mixing 10 μL of test serum mixed with 10 μL bacterial suspension, while Zone B was prepared by mixing 10 μL of test serum with 10 μL negative control (suspension of *E. coli* W3110 wild-type strain). The slide was incubated at room temperature for 15 min, and agglutination was evaluated through visual observation. A positive result was defined as the formation of visible granular or flocculent aggregates, contrasting with the uniform suspension maintained in Zone B.

### 2.8. Animal Immunization and Challenge

BALB/c mice were subcutaneously injected with different vaccine formulations on days 0, 14, and 28. The monovalent vaccine efficacy evaluation included the following groups: C-OPS_ECO1_ (containing 2.5 μg OPS_ECO1_), C-OPS_ECO2_ (containing 2.5 μg OPS_ECO2_), C-OPS_ECO6_ (containing 2.5 μg OPS_ECO6_), C-OPS_ECO25_ (containing 5.0 μg OPS_ECO25_), and PBS (negative control). For tetravalent vaccine assessment, experimental groups comprised: (1) ExPEC 4V (C-OPS_ECO1_:C-OPS_ECO2_:C-OPS_ECO6_:C-OPS_ECO25_ = 2.5:2.5:2.5:5.0 μg); (2) monovalent controls: C-OPS_ECO1_ (containing 2.5 μg OPS_ECO1_), C-OPS_ECO2_ (containing 2.5 μg OPS_ECO2_), C-OPS_ECO6_ (containing 2.5 μg OPS_ECO6_), or C-OPS_ECO25_ (containing 5.0 μg OPS_ECO25_); (3) PBS. Blood samples were collected via retro-orbital plexus on day 35. Serum samples, obtained by centrifuging clotted blood at 6000 rpm for 40 min (4 °C), were stored at −80 °C.

### 2.9. Enzyme-Linked Immunosorbent Assay (ELISA)

A 96-well plate was precoated with 100 µg/mL of the corresponding serotype LPS solution (100 μL/well) and incubated overnight at 4 °C. Three automatic washes with PBST were performed using a microplate washer (BioTek, Shoreline, WA, USA). Subsequently, blocking solution (200 μL/well) was added, and blocked for 2 h at 37 °C, followed by washing as before. The serum samples were diluted in a series with incubation buffer, starting with an initial dilution of 1:20 (mixing 10 μL of serum with 190 μL of 0.5% skim milk powder), followed by two-fold serial dilutions. They were incubated with the primary antibody for 1 h at 37 °C, then the plate was washed three times with PBST. Horseradish peroxidase-labeled secondary antibody was added (100 μL/well) and incubated for 1 h at 37 °C. After the final wash, TMB substrate was added (100 μL/well) and the reaction was allowed to proceed in the dark for 5 to 10 min. The reaction was terminated by adding Elisa stop solution. Optical density was measured at 450 nm using a microplate reader (BioTek, USA).

### 2.10. Lethal Dose Challenge of E. coli O1, E. coli O2, E. coli O6, or E. coli O25 Infection in BALB/c Mouse Model

Batch cultivation of *E. coli* serotypes O1, O2, O6, and O25 was performed in 5.0 mL LB broth. Cultures were incubated at 37 °C under aerobic conditions until reaching late-exponential growth phase. Bacterial harvesting was conducted through centrifugation at 6000 rpm for 3 min. The washed pellets were resuspended in sterile PBS and subsequently serially diluted (10-fold gradient series). Bacterial enumeration was performed via standardized plate counting methodology, wherein 100 μL aliquots of appropriate dilutions were spread onto LB agar plates followed by 12 h aerobic incubation at 37 °C for colony enumeration. On day 14 post-immunization, immunized mice were injected intraperitoneally with approximately 9.0 × 10^7^ CFU of *E. coli* O1, 2.3 × 10^7^ CFU of *E. coli* O2, 1.2 × 10^8^ CFU of *E. coli* O6, or 2.4 × 10^8^ CFU of *E. coli* O25 per mouse. Survival status was checked at 12 h intervals for 48 h post-challenge.

### 2.11. Sublethal Dose of E. coli O1, E. coli O2, E. coli O6, or E. coli O25 Infection in BALB/c Mouse Model

Bacterial culture and dilution methods followed Section 2.10 protocols. On day 14 post-immunization, immunized mice were injected intraperitoneally with approximately 5.0 × 10^6^ CFU of *E. coli* O1, 2.6 × 10^6^ CFU of *E. coli* O2, 1.3 × 10^7^ CFU of *E. coli* O6, or 3.6 × 10^7^ CFU of *E. coli* O25 per mouse.

Blood samples were collected from the tail vein at 0 h, 6 h, 12 h, 1 day, 3 days, and 5 days post-infection. Serum cytokine (TNF-α and IL-6) levels were determined using pre-coated ELISA kits (TNF-α ELISA Kit, Dakewe, Shenzhen, China; IL-6 ELISA Kit, Dakewe, Shenzhen, China) following serum separation. Body weights were monitored daily for five days post-infection. On day 1 post-infection, mice that aimed to detect bacterial loads in major organs were euthanized via cervical dislocation under aseptic conditions. Major organs (spleens and kidneys) were excised and homogenized in 1 mL of sterile PBS using a tissue homogenizer, followed by filtration through 70-µm sieves. The resulting homogenates were serially diluted in physiological saline and plated on LB agar plates. Following overnight incubation at 37 °C, bacterial loads were determined by enumerating CFU per gram of tissue.

### 2.12. Statistical Analysis

Antibody titers and bacterial loads were subjected to log10 transformation. S Statistical analysis was performed using GraphPad Prism version 8.0 (GraphPad, La Jolla, CA, USA). Data were analyzed using one-way ANOVA followed by Dunn’s multiple comparison test. All the data were expressed as means ± SD (standard deviation). A *p*-value less than 0.05 was considered statistically significant (**** *p* < 0.0001, *** *p* < 0.001, ** *p* < 0.01, and * *p* < 0.05).

## 3. Results

### 3.1. The Construction of the OPS_ECO1_, OPS_ECO2_, OPS_ECO6_, and OPS_ECO25_ Synthesis Plasmid and Its Functional Validation in E. coli

**Figure 1:**
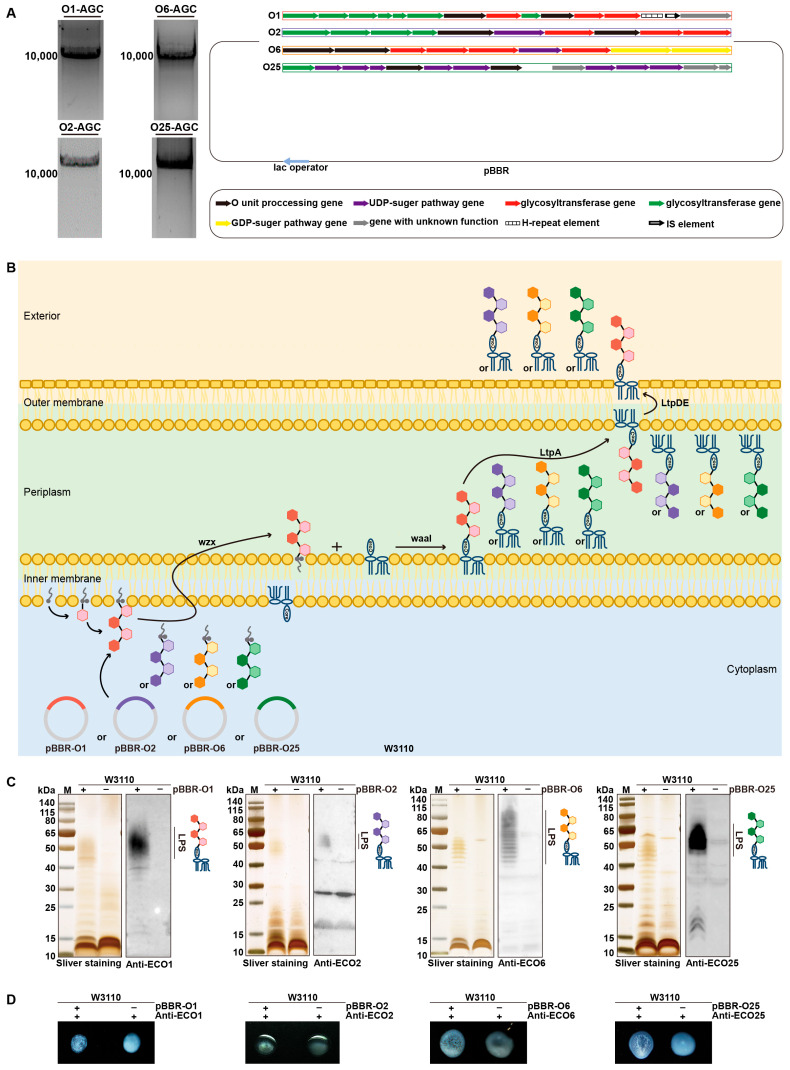
Synthesis of *Escherichia coli* O1, O2, O6, and O25 OPS in *E. coli* W3110. (**A**) Gel images of PCR products of *E. coli* O1, O2, O6, and O25 O antigen gene cluster (O-AGC). (**B**) Schematic diagram of *E. coli* O1, O2, O6, and O25 LPS synthesis in *E. coli* W3110. (**C**) Sliver staining and Western blot of W3110/pBBR-O1, W3110/pBBR-O2, W3110/pBBR-O6, W3110/pBBR-O25. (**D**) Agglutination Assay of W3110/pBBR-O1, W3110/pBBR-O2, W3110/pBBR-O6, W3110/pBBR-O25.

Through the construction of customized plasmids, we successfully synthesized the OPS of *E. coli* O1, O2, O6, and O25 serotypes. Gene clusters governing OPS biosynthesis in these serotypes measured 10,324 bp (O1), 13,232 bp (O2), 11,249 bp (O6), and 15,009 bp (O25), containing respectively 15, 10, 9, and 14 functional genes (Figure 1A and Figure S1).

To verify OPS synthesis, pBBR-O1, pBBR-O2, pBBR-O6, and pBBR-O25 were transformed into *E. coli* W3110, with loss of LPS due to its deficiency in the endogenous OPS biosynthesis (Figure 1B). Following IPTG induction and overnight culture, whole cell lysates were subjected to SDS-PAGE analysis, with subsequent silver stain and Western blot using serotype-specific anti-OPS sera. Western blot demonstrated that the synthesized LPS, which was recovered by the heterologous OPS synthetic gene cluster, specifically reacted with respective serotype-specific antibodies (Figure 1C). Furthermore, in agglutination assays, when overnight cultures were incubated with their respective serotype-specific antibodies, *E. coli* W3110 strains harboring pBBR-O1 or pBBR-O2 or pBBR-O6 or pBBR-O25 exhibited evident agglutination. In contrast, no agglutination was observed in the wild-type *E. coli* W3110 strain (Figure 1D). These results collectively demonstrate the successful construction of OPS-biosynthesis plasmid vectors (pBBR-O1, pBBR-O2, pBBR-O6, and pBBR-O25) and verify their ability to direct OPS biosynthesis by heterologous LPS production in *E. coli* W3110.

### 3.2. Synthesis of Candidate Bioconjugate Vaccines in Engineered E. coli

Following confirmation of successful heterologous expression of *E. coli* O1, O2, O6, and O25 LPS in *E. coli* W3110, we separately introduced pBBR-O1, pBBR-O2, pBBR-O6, and pBBR-O25 into the engineered strain *E. coli* WdlO-d01, optimized for recombinant polysaccharide production. Concomitantly, the plasmid pET32a-*pglL*-CTB4573, which encodes the OST PglL and the carrier protein cholera toxin B subunit (CTB) tagged with glycosylation sequence 4573 was co-introduced to enable OPS–protein bioconjugation.

**Figure 2:**
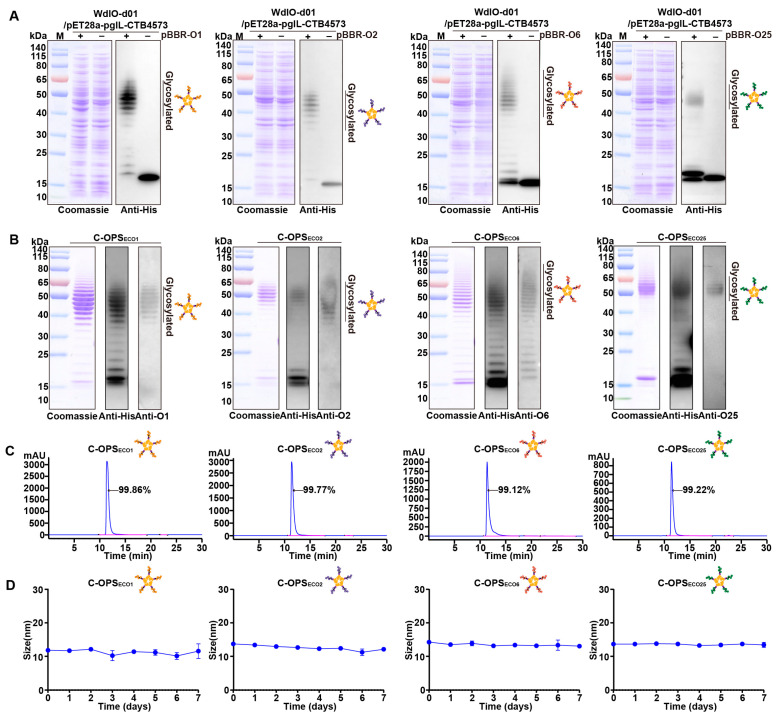
Expression and verification of the glycoprotein. (**A**) Glycoproteins were detected in strains WdlO-d01. (**B**) Purified C-OPS_ECO1_, C-OPS_ECO2_, C-OPS_ECO6_ and C-OPS_ECO25_ samples were separated by SDS-PAGE and analyzed via Coomassie blue staining, Western blot using antibody against 6 × His Tag and serum against *E. coli* O1, *E. coli* O2, *E. coli* O6 and *E. coli* O25. (**C**) HPLC analysis of C-OPS_ECO1_, C-OPS_ECO2_, C-OPS_ECO6_, C-OPS_ECO25_. (**D**) The size of C-OPS_ECO1_, C-OPS_ECO2_, C-OPS_ECO6_ and C-OPS_ECO25_ at different times.

Following IPTG induction, whole-cell lysates were analyzed by SDS-PAGE and Western blot using an anti-6 × His antibody. Ladder bands were observed in strains co-expressing the O-AGC with CTB, whereas parental CTB bands remained unmodified in OPS-deficient controls (Figure 2A). This glycosylation signature conclusively demonstrates that OPS was successfully conjugated to CTB via PglL, confirming covalent attachment of polysaccharide–protein complexes.

The four glycoconjugates were purified sequentially by affinity chromatography and size-exclusion chromatography. Coomassie blue stain and Western blot with anti-6 × His Tag antibodies and serotype-specific anti-OPS sera consistently revealed target bands corresponding to the expected molecular weights (Figure 2B). High-performance liquid chromatography further confirmed monodisperse elution peaks with purity exceeding 99% (Figure 2C). Endotoxin levels quantified via the limulus amebocyte lysate assay (Table S3) confirmed that stringent vaccine safety standards had been followed for the above protein preparations. Stability analysis of C-OPS_ECO1_, C-OPS_ECO2_, C-OPS_ECO6_, and C-OPS_ECO25_ under 37 °C revealed no significant changes in size over 7 days (Figure 2D) and the size of the four glycoconjugates is about 13 nm (Figure S2), indicating the four glycoconjugates could be kept stable after being stored for a long time.

### 3.3. Safety Evaluation of Bioconjugate Vaccine

**Figure 3:**
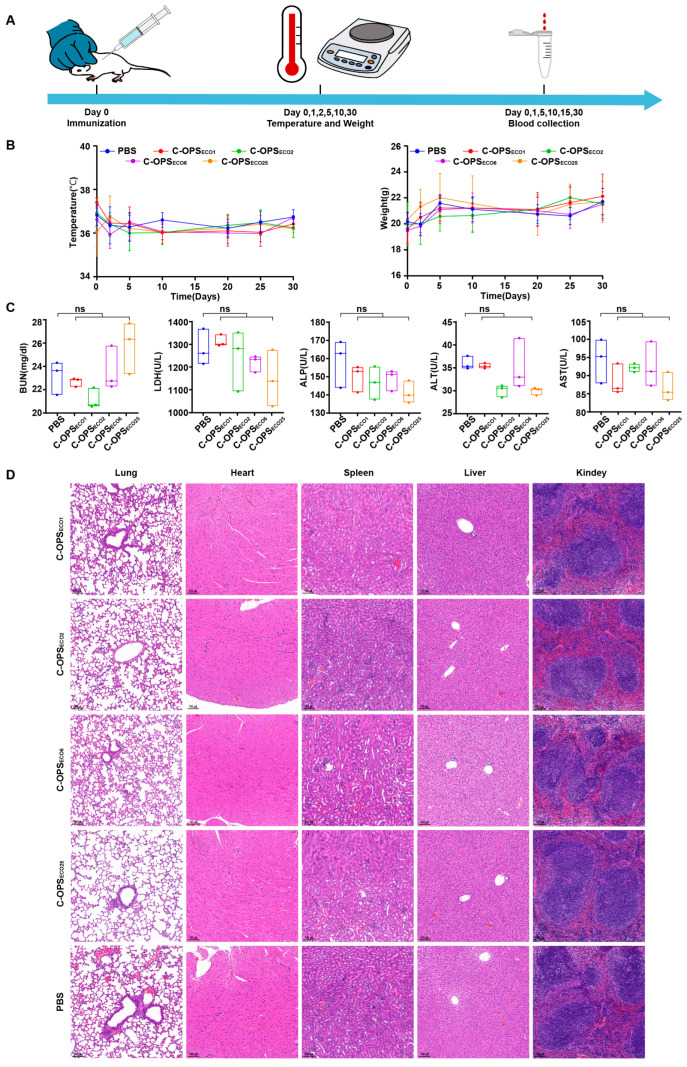
Safety evaluation of candidate bioconjugate vaccines. (**A**) The overall process for evaluating vaccine safety. (**B**) Changes in body temperature and weight of mice during the observation period (n = 5). (**C**) Biochemical indicators (including ALT, AST, BUN, LDH, and ALP) in serum 30 days after immunization (n = 3). (**D**) HE staining analysis of the lungs, heart, spleen, liver, and kidneys of mice.

Before evaluating vaccine protective efficacy, we systematically assessed the safety of the vaccine using the BALB/c mouse model. BALB/c mice received 5-fold standard doses: C-OPS_ECO1_, C-OPS_ECO2_, and C-OPS_ECO6_ groups (12.5 µg polysaccharide), and the C-OPS_ECO25_ group (25.0 µg polysaccharide), with PBS controls. Post-immunization evaluations included continuous monitoring of body temperature and weight variations, alongside quantitative analysis of serum inflammatory cytokines (IL-1β, IL-6) and biochemical indicators reflecting systemic toxicity (Figure 3A). Longitudinal monitoring of body temperature and weight changes (post-immunization days 0, 2, 5, 10, 20, 25, 30) revealed parallel trends between treated and control mice (Figure 3B), demonstrating immunological safety. Serum biochemical analysis (BUN, LDH, ALP, ALT, AST) confirmed all parameters remained within normal physiological ranges (Figure 3C), corroborating initial observations. Further, histopathological evaluation of major organs (lung, heart, spleen, liver, and kidney) showed comparable tissue architecture between treated and control groups, with no detectable pathological lesions (Figure 3D). Finally, serum cytokine (TNF-α and IL-6) levels were assessed at specified time points in all experimental groups. Notably, both cytokines remained at minimal concentrations throughout the observation period (Figure S3). The collective evidence from physiological monitoring, tissue histology, and blood biochemistry conclusively establishes the safety of this vaccine formulation at 5-fold overdosage, supporting its progression to efficacy evaluation.

### 3.4. Evaluation of Specific Antibodies After Immunization with C-OPS_ECO1_, C-OPS_ECO2_, C-OPS_ECO6_, or C-OPS_ECO25_

**Figure 4:**
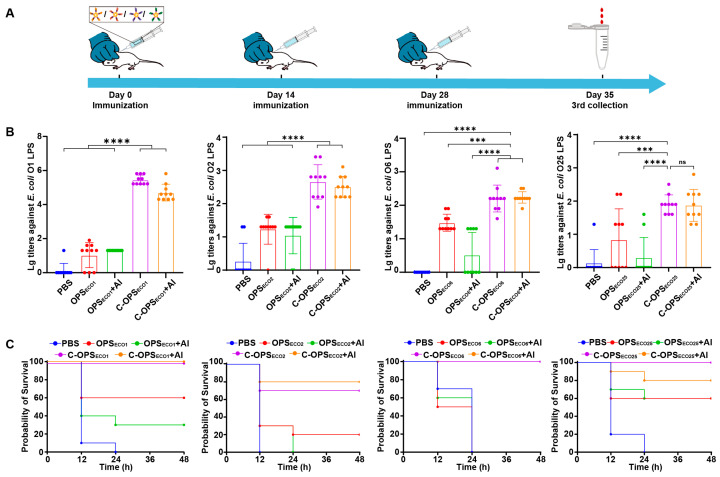
C-OPS_ECO1_, C-OPS_ECO2_, C-OPS_ECO6_, and C-OPS_ECO25_ elicit robust prophylactic effects against bacterial infection (n = 10). (**A**) Immunization schedule for further evaluation. (**B**) The IgG titers against the LPS of *E. coli* O1, *E. coli* O2, *E. coli* O6, and *E. coli* O25. (**C**) Mice were infected with lethal dose of *E. coli* O1, *E. coli* O2, *E. coli* O6, or *E. coli* O25 cells per mouse and their survival rates were measured. Each group was compared with C-OPSs and C-OPSs+Al by one-way analysis of variance (ANOVA) with Dunn’s multiple comparison test: **** *p* < 0.0001, *** *p* < 0.001.

To validate the serotype-specific antibody induction capacity of four glycoprotein formulations, BALB/c mice were subcutaneously immunized on days 0, 14, and 28 with either C-OPS or OPS formulations, both with or without 10% aluminum hydroxide adjuvant (Figure 4A). The immunization protocol comprised the following: O1, O2, and O6 groups received 2.5 µg polysaccharide per mouse; the O25 group received 5.0 µg polysaccharide and PBS control group. In line with the vaccine developed by Janssen company, the immunization dosage for the O25 group was doubled compared to other serotypes in this experimental design, since our data also showed that 2.5 μg polysaccharide in the O25 group did not elicit effective antibody responses (Figure S4). ELISA quantification of LPS-specific IgG antibodies revealed that C-OPS formulations elicited significantly higher antibody titers compared to their aluminum-adjuvanted counterparts (C-OPS + adjuvant), with both groups surpassing the immunogenicity of OPS formulations (adjuvanted/non-adjuvanted), and PBS controls (Figure 4B).

Then, we injected a lethal dose of *E. coli* O1, *E. coli* O2, *E. coli* O6, or *E. coli* O25 intraperitoneally into mice to assess the protective effect of C-OPS on mice (14 days post-immunization). At 48 h after infection, we observed the survival curves of the mice in each group. Notably, monovalent vaccine groups exhibited robust protection rates of 100% (O1, O6, and O25) and 80% (O2), respectively (Figure 4C). Thus, C-OPS_ECO1_, C-OPS_ECO2_, C-OPS_ECO6_, or C-OPS_ECO25_ significantly enhanced immune responses and effectively protected mice against ExPEC infection.

### 3.5. Evaluation of Specific Antibodies After Immunization with ExPEC 4V

**Figure 5:**
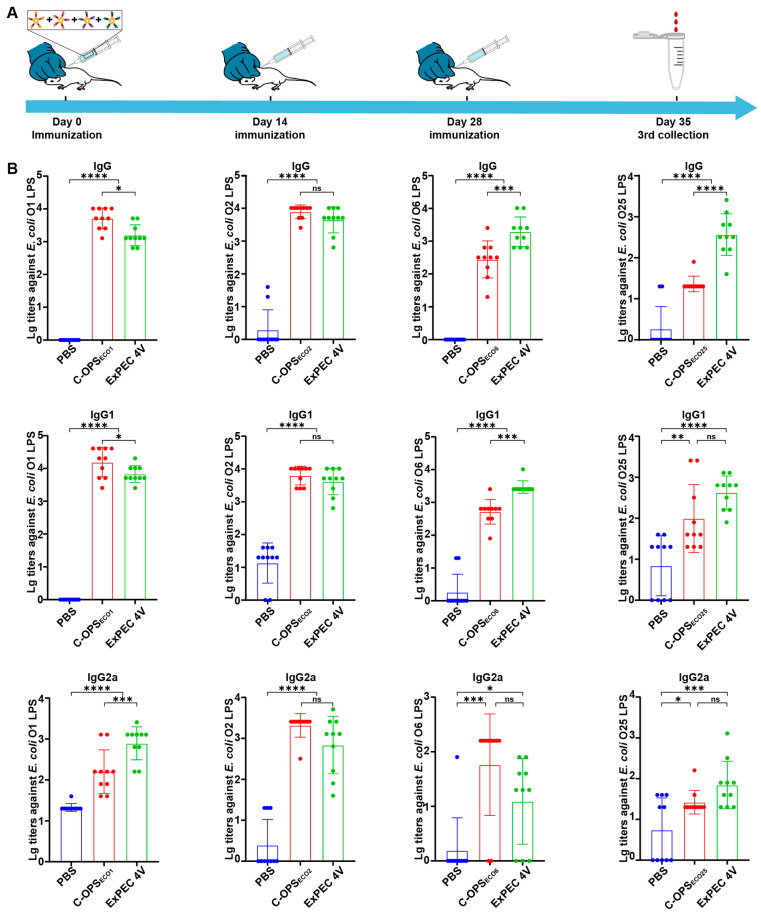
IgG responses of ExPEC 4V (C-OPS_ECO1_:C-OPS_ECO2_:C-OPS_ECO6_:C-OPS_ECO25_ = 2.5:2.5:2.5:5.0 µg) against the LPS of *E. coli* O1, *E. coli* O2, *E. coli* O6, and *E. coli* O25 (n = 10). (**A**) Immunization schedule for further evaluation. (**B**) IgG titers against the LPS of *E. coli* O1, *E. coli* O2, *E. coli* O6, and *E. coli* O25. Each group was compared with C-OPSs and ExPEC 4V by one-way analysis of variance (ANOVA) with Dunn’s multiple comparison test: **** *p* < 0.0001, *** *p* < 0.001, ** *p* < 0.01, and * *p* < 0.05.

After assessing the safety of ExPEC 4V using BALB/c mouse models (Figure S5), we conducted comparative immunization in BALB/c mice using the tetravalent formulation (C-OPS_ECO1_:C-OPS_ECO2_:C-OPS_ECO6_:C-OPS_ECO25_ = 2.5:2.5:2.5:5.0 µg) with PBS serving as negative control. Following the immunization regimen as previously described (days 0, 14, and 28) (Figure 5A), the ELISA results demonstrated that the O1 and O2 groups showed moderate decreases in LPS-specific IgG titers relative to monovalent controls, the O6 and O25 groups conversely demonstrated elevated antibody responses, and all maintained significantly higher antibody concentrations than PBS controls (Figure 5B). Further, we evaluated IgG subtypes (including IgG1 and IgG2a) post-immunization. Both tetravalent and monovalent vaccines exhibited significantly higher antibody titers than the PBS control group (Figure 5), which aligns with findings from prior immunization studies.

### 3.6. Evaluation of Bioconjugate Vaccine-Induced Protection After Infection with Varying Doses in Mice

**Figure 6:**
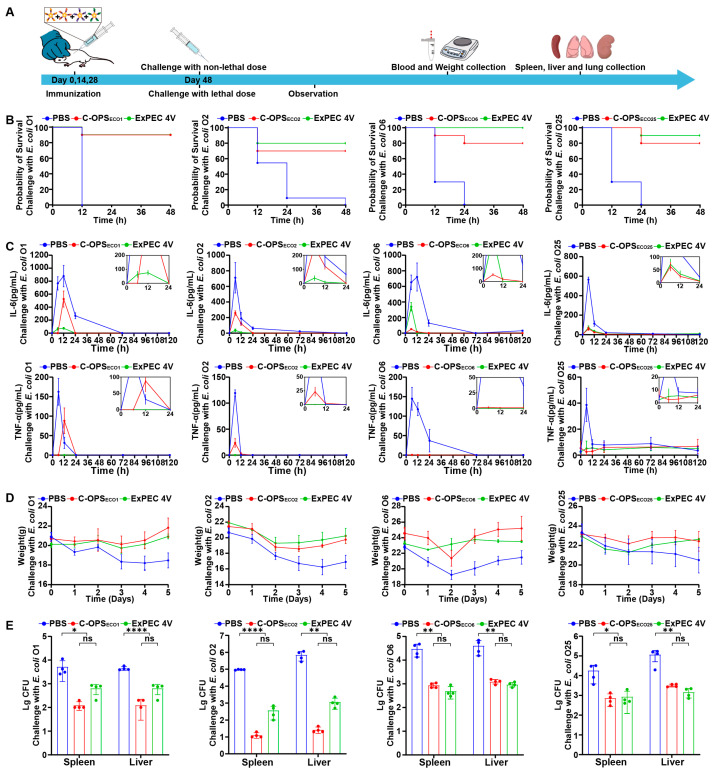
Evaluation of ExPEC 4V-mediated protection against different doses of *E. coli* O1, *E. coli* O2, *E. coli* O6, or *E. coli* O25. (**A**) Schematic diagram of the establishment of the infection model and subsequent evaluations. (**B**) Mice were infected with a lethal dose of *E. coli* O1, *E. coli* O2, *E. coli* O6, or *E. coli* O25 cells per mouse and their survival rates were measured (n = 10). (**C**) After infection with a sublethal dose of *E. coli* O1, *E. coli* O2, *E. coli* O6, or *E. coli* O25, venous blood was collected at 0 h, 6 h, 12 h, 1 day, 3 days, and 5 days, and the levels of TNF-α and IL-6 in the sera were measured by ELISA (n = 3). (**D**,**E**) Mice were challenged with a sublethal dose of *E. coli* O1, *E. coli* O2, *E. coli* O6, or *E. coli* O25. We determined changes in weight (n = 3) and counted bacterial loads in blood and two organs (spleen and liver) 24 h post-challenge (n = 4). Each group was compared with C-OPSs and ExPEC 4V by one-way analysis of variance (ANOVA) with Dunn’s multiple comparison test: **** *p* < 0.0001, ** *p* < 0.01, and * *p* < 0.05.

To comprehensively evaluate the protective efficacy of the tetravalent vaccine, we employed both lethal and sublethal challenge models (Figure 6A). For lethal dose evaluation, we injected a lethal dose of *E. coli* O1, *E. coli* O2, *E. coli* O6 or *E. coli* O25 intraperitoneally into mice to assess the protective effect of ExPEC 4V on mice (14 days post-immunization). At 48 h after infection, we observed the survival curves of the mice in each group. The tetravalent formulation demonstrated serotype-specific protection rates of 90% (O1), 80% (O2), 100% (O6), and 90% (O25) (Figure 6B).

Subsequent challenge with a sublethal dose of *E. coli* O1, *E. coli* O2, *E. coli* O6, or *E. coli* O25 revealed significant bacterial clearance enhancement in vaccinated mice, with 1–3 log reductions in bacterial loads from spleen and liver tissues compared to PBS controls (Figure 6C). Longitudinal monitoring over 5 days post-sublethal challenge showed less body weight fluctuations in vaccinated groups (Figure 6D), accompanied by suppressed systemic inflammation as evidenced by significantly lower serum levels of IL-6 and TNF-α versus control groups (Figure 6E). These coordinated findings across survival outcomes, bacterial clearance, physiological stability, and inflammatory modulation collectively demonstrate the multi-faceted protective capacity of the tetravalent vaccine against ExPEC infections.

## 4. Discussion

The growing clinical incidence of ExPEC infections across both healthcare and community settings, coupled with expanding antimicrobial resistance patterns in this pathogen, has intensified demand for effective preventive vaccines. The development of ExPEC vaccines has progressed through three principal strategies [31]. Whole-cell vaccines (e.g., Uro-Vaxom [32] and Solco-Urovac [33]) utilize whole-cell preparations in live-attenuated or inactivated forms to induce broad-spectrum immune protection. Protein subunit vaccines focus on conserved virulence determinants including FimCH [34] and iron acquisition systems (IutA/IroN) [35,36], directing adaptive immunity toward ExPEC-specific immunodominant antigens. Notably, the FimCH candidate vaccine has progressed to phase II clinical trials [34]. The third strategy, implemented in this study, employs engineered *E. coli* to biosynthesize polysaccharide-protein conjugates targeting the OPS of predominant ExPEC serotypes, thereby eliciting pathogen-specific IgG responses against ExPEC.

This study presents a bioconjugate vaccine candidate targeting four predominant ExPEC serotypes (O1, O2, O6, and O25) using PGCT. The key technical step of this strategy is obtaining the O antigen synthesis gene cluster, commonly as large DNA fragments that are difficult to clone accurately due to the spontaneous mutations that occur during PCR. Although long-range PCR enabled successful amplification of the target gene cluster in this study, minor random mutations were still observed in amplified sequences. Fortunately, these sequences showed no impact on protein functionality according to the results of Western blot analysis and agglutination assay. This technical limitation highlights the potential value of developing next-generation cloning strategies, particularly large-fragment capture systems analogous to the RecET system [37]. Such technological advances could provide transformative tools for precise manipulation of biosynthetic pathways, dramatically accelerating rational design of recombinant vaccines.

Then, we introduced OPS-biosynthesis plasmid into engineered strain *E. coli* WdlO-d01 for heterologous glycoprotein biosynthesis. The purified proteins met the vaccine quality standards through comprehensive characterization including stability testing, HPLC analysis, and endotoxin quantification. Animal experimental results demonstrated that C-OPS_ECO1_, C-OPS_ECO2_, C-OPS_ECO6_, and C-OPS_ECO25_ effectively elicited robust antibody responses against *E. coli* O1, *E. coli* O2, *E. coli* O6, or *E. coli* O25. Notably, these antigens conferred over 80% protection against lethal challenges of four *E. coli* serotypes. C-OPS_ECO1_, C-OPS_ECO2_, C-OPS_ECO6_, and C-OPS_ECO25_ were also tested in combination with adjuvant (alum), but the adjuvant effect did not increase the magnitude of the antibody responses, which was also observed in the vaccine developed by the Janssen company.

This informed our decision to develop an adjuvant-free tetravalent formulation. The final ExPEC 4V vaccine combines four antigens at an optimized ratio (C-OPS_ECO1_:C-OPS_ECO2_:C-OPS_ECO6_:C-OPS_ECO25_ = 2.5:2.5:2.5:5.0 µg). Elisa results showed no significant interference in vaccine-specific titers upon antigen combination, with enhanced immunogenicity observed for the O6 and O25 groups. Subsequent challenge experiments using both lethal and sublethal doses demonstrated that ExPEC 4V maintained significant protective efficacy against ExPEC infections. The lethal challenge model, widely adopted in sepsis research, has been extensively applied in vaccine studies to evaluate protection efficacy against diverse pathogens. The integration of two infection models provides more comprehensive and intuitive assessment of vaccine efficacy. Notably, both experimental models demonstrated that the engineered *E. coli*-derived bioconjugate vaccines conferred effective protection against bacterial infections, indicating promising clinical translational potential.

## 5. Conclusions

Our study successfully validates the technical feasibility of PglL-mediated OPS-protein conjugation. Consistent with the similar vaccine developed by Janssen company [38], our candidate vaccine employs OPS from the same four serotypes as antigens. This selection rationale is supported by epidemiological evidence confirming the clinical relevance of this serotype combination to urinary tract infections and bacteremia [39]. Regarding carrier protein selection, CTB presents distinct molecular advantages over the traditional recombinant exoprotein A. In nature, CTB can form a stable pentameric ring, which enhances its structural complexity and is beneficial for activating immune responses. Additionally, due to CTB’s ability to bind to GM1 on the cell surface, vaccines based on CTB exhibit receptor effects that facilitate efficient antigen delivery and presentation. The difference in antibody response between the two carriers has been detected in our previous research. The pentameric structure of CTB (~8 nm) facilitates targeted delivery to antigen-presenting cells (macrophages, dendritic cells, etc.) through its GM1 receptor-binding mechanism [40]. This targeting capability stems from the ubiquitous expression of GM1 receptors across innate immune system components. Notably, the CTB pentamer facilitates clustered presentation of antigenic polysaccharides, thereby substantially enhancing recognition efficiency by APCs through multivalent binding interactions. However, direct comparative analysis between our candidate and the vaccine developed by the Janssen company remains technically unfeasible at this stage, owing to the absence of standardized reference sera. For the comparison of the two candidate vaccines, different preparation systems, quality control standards, and experimental procedures have potential impacts on the results. While the use of standardized reference serum, such as the international standard COVID-19 serum established by the WHO [41], is essential for evaluating the performance of various vaccines, there is currently no corresponding standard serum available for ExPECs. Therefore, direct comparative analysis between our candidate and the vaccine developed by the Janssen company remains technically unfeasible at this stage.

## Data Availability

The original contributions presented in this study are included in the article/Supplementary Materials, and further inquiries can be directed to the corresponding author.

## Supplementary Materials

The following supporting information can be downloaded at: https://www.mdpi.com/article/10.3390/vaccines13040362/s1, Table S1: Bacterial strains and plasmids used in this study; Table S2: All primers used to construct and confirm gene mutants in this study; Table S3: The endotoxin content of C-OPS_ECO1_, C-OPS_ECO2_, C-OPS_ECO6_, and C-OPSE_CO25_; Figure S1: *E. coli* O1, *E. coli* O2, *E. coli* O6, and *E. coli* O25 O-polysaccharide gene cluster and functional region; Figure S2: Dynamic light scattering analysis of C-OPS_ECO1,_ C-OPS_ECO2,_ C-OPS_ECO6_ and C-OPS_ECO25_; Figure S3: Changes in serum TNF-α and IL-6 levels in mice (n = 3) following administration of 5 × the candidate bioconjugate vaccine during the safety evaluation observation period; Figure S4: On the 7th day post-immunization with C-OPS_ECO25_ (2.5 µg OPS_ECO25_ per mouse), the IgG titer against the LPS of *E. coli* O25 was measured in serum; Figure S5: Safety evaluation of ExPEC 4V (n = 3); Figure S6: The whole blot (uncropped blots) of Figure 1C; Figure S7: The whole blot (uncropped blots) of Figure 2A; Figure S8: The whole blot (uncropped blots) of Figure 2B; Figure S9: Glycoproteins were detected in strains WdlO-d01 via Western blot using antibody against serum against *E. coli* O1, *E. coli* O2, *E. coli* O6 and *E. coli* O25.

## Abbreviations

The following abbreviations are used in this manuscript:

ExPEC	Extra-intestinal pathogenic *Escherichia coli*
MDR	Multidrug-resistant
PGCT	Protein glycan coupling technology
O-AGC	O-antigen gene cluster
OPS	O-antigen polysaccharide
LPS	Lipopolysaccharide
OST	Oligosaccharyltransferase
IPTG	Isopropyl-β-D-thiogalactopyranoside
ELISA	Enzyme-linked immunosorbent assay
CTB	Cholera toxin B subunit
