# Supplementary material for: A Bioconjugate Vaccine Against Extra-Intestinal Pathogenic Escherichia coli (ExPEC)
Source: Vaccines (Basel). 2025 Mar 28;13(4):362. doi: 10.3390/vaccines13040362 (PMC12031530; doi:10.3390/vaccines13040362)
Supplement: Supplementary file 1 [file vaccines-13-00362-s001.zip › vaccines-3520996-supplementary.pdf]

Supplementary Materials

A Bioconjugate Vaccine Against Extra-Intestinal Pathogenic *Escherichia coli* (ExPEC)

Linhui Hao [1,†], Wenhua Huang [2,†], Yan Guo [1], Xiankai Liu [1], Jun Wu [1], Li Zhu [1], Chao Pan [1,*] and Hengliang Wang [2,*]

1 Laboratory of Advanced Biotechnology, Beijing Institute of Biotechnology, Beijing 100071, China

2 State Key Laboratory of Pathogen and Biosecurity, Academy of Military Medical Sciences, Beijing 100071, China

* Correspondence: panchao@bmi.ac.cn (C.P.); wanghl@bmi.ac.cn (H.W.)

† These authors contributed equally to this work.

Table S1. Bacterial strains and plasmids used in this study

Bacterial Strains and Plasmids	Characteristic	Source
E. coli W3110	Gene *wbbL* was inactivated	Laboratory stock
E. coli WdlO-d01	Absence of *waaL* and *wbbH-L* genes	Laboratory stock
E. coli O1	*E. coli* O1 serotype	Provided by Professor Yunsong Yu
E. coli O2	*E. coli* O2 serotype	Provided by Professor Yunsong Yu
E. coli O6	*E. coli* O6 serotype	Provided by Professor Yunsong Yu
E. coli O25	*E. coli* O25 serotype	Provided by Professor Yunsong Yu
E. coli W3110/pBBR-O1	Heterologous synthesis of *E. coli* O1 OPS	This study
E. coli W3110/pBBR-O2	Heterologous synthesis of *E. coli* O2 OPS	This study
E. coli W3110/pBBR-O6	Heterologous synthesis of *E. coli* O6 OPS	This study
E. coli W3110/pBBR-O25	Heterologous synthesis of *E. coli* O25 OPS	This study
E. coli WdlO-d01/pET32a-pglL-CTB4573/ pBBR-O1	Expression of glycoprotein C-OPS_{ECO1}	This study
E. coli WdlO-d01/ pET32a-pglL-CTB4573/ pBBR-O2	Expression of glycoprotein C-OPS_{ECO2}	This study
E. coli WdlO-d01/ pET32a-pglL-CTB4573/ pBBR-O6	Expression of glycoprotein C-OPS_{ECO6}	This study
E. coli WdlO-d01/ pET32a-pglL-CTB4573/ pBBR-O25	Expression of glycoprotein C-OPS_{ECO25}	This study
pBBR-O1	Encodes OPS of *E. coli* O1, Kanr	This study
pBBR-O2	Encodes OPS of *E. coli* O 2, Kanr	This study
pBBR-O6	Encodes OPS of *E. coli* O6, Kanr	This study
pBBR-O25	Encodes OPS of *E. coli* O25, Kanr	This study
pET32a-pglL-CTB4573	Encodes PglL and 6×His-tagged CTB fused DsbA signal peptide at N-terminus and glycosylation sequence (4573) fragment at C-terminus, Ampr	This study

Table S2. All primers used to construct and confirm gene mutants in this study

Primer	Sequence
pBBRO1-F	ATGGTGAAGATACTTGTTACTGGGGGCGCAGGA
pBBRO1-R	CGTATAGTAAGGAACCAGTTTCTTGCCTG
pBBRO2-F	GCAGGATTTATTGGTTCTGCTG
pBBRO2-R	TGATTTGATGACCATAACCGCAT
pBBRO6-F	AATAGGATACAACGTGTGCATAATTATTTAAGGC
pBBRO6-R	GAATGGTGTCCTGGAAGAAGGTGTTACCA
pBBRO25-F	CACAGGAAACAGCTATGGTGAAGATAC
pBBRO25-R	CGCTTTATAACCACTTAGAAAAAGCC
pBBRO1-PF	GTAACAAGTATCTTCACCATAGCTGTTTCCTGTGTGAAATTGTTA
pBBRO1-PR	AAACTGGTTCCTTACTATACGGAGGTCGACGGTATCGATAAGC
pBBRO2-PF	TATGCGGTTATGGTCATCAAATCAGAGGTCGACGGTATCG
pBBRO2-PR	CAGCAGAACCAATAAATCCTGCCATAGCTGTTTCCTGTGTGAAA TTG
pBBRO6-PF	TGCACACGTTGTATCCTATTCATAGCTGTTTCCTGTGTGAAATTGT TA
pBBRO6-PR	CTTCTTCCAGGACACCATTCGAGGTCGACGGTATCGATAAGC
pBBRO25-PF	CTTTTTCTAAGTGGTTATAAAGCGATATACCACCGTTTATTCTTC
pBBRO25-PR	CCAGTAACAAGTATCTTCACCATAGCTGTTTCCTGTGTGAAATTG
pBBRO1-YF	TTTCTAATGAATCTGACCTCGCA
pBBRO1-YR	GATGAGCCGTGTTTTCTGGAC
pBBRO2-YF	AAAGGTTGGGCTTCGGAATCG
pBBRO2-YR	CATCACTGCATCCGGCTGAT
pBBRO6-YF	TGACCGCTGTTTCCTGTTTG
pBBRO6-YR	CGGTAAACCAGCAATAGACATAAG
pBBRO25-YF	CTGGCGATTCAGGTTCATCA
pBBRO25-YR	GCCCTCATAGTTCTGTTCAATCC

Table S3 The endotoxin content of C-OPS$_{ECO1}$, C-OPS$_{ECO2}$, C-OPS$_{ECO6}$, and C-OPSE$_{CO25}$

Conjugate	C-OPS$_{ECO1}$	C-OPS$_{ECO2}$	C-OPS$_{ECO6}$	C-OPS$_{ECO25}$
Endotoxin (EU/mouse)	0.15 ± 0.02	0.35 ± 0.19	0.28 ± 0.81	0.08 ± 0.09

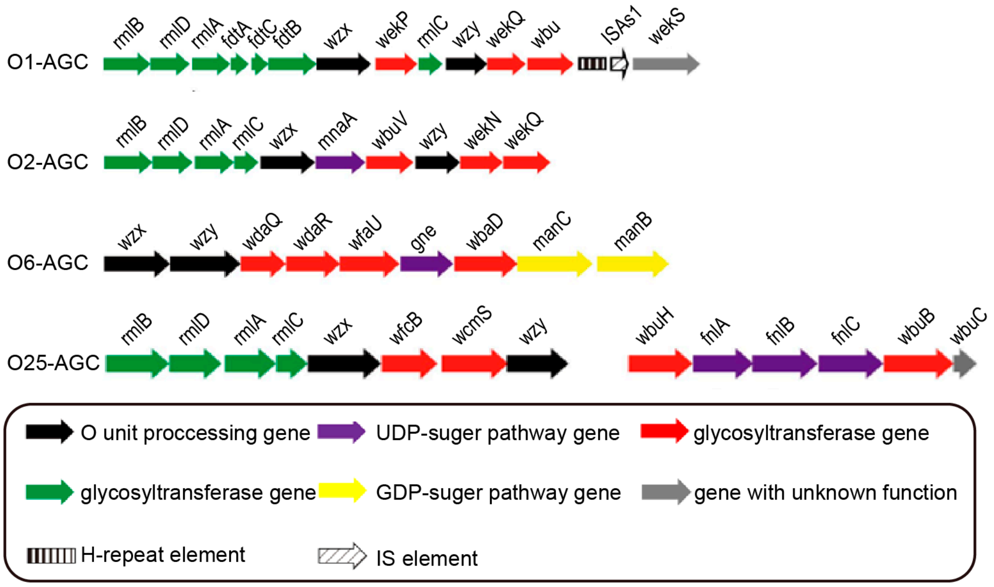

Figure S1. *E. coli* O1, *E. coli* O2, *E. coli* O6, and *E. coli* O25 O-polysaccharide gene cluster and functional region.

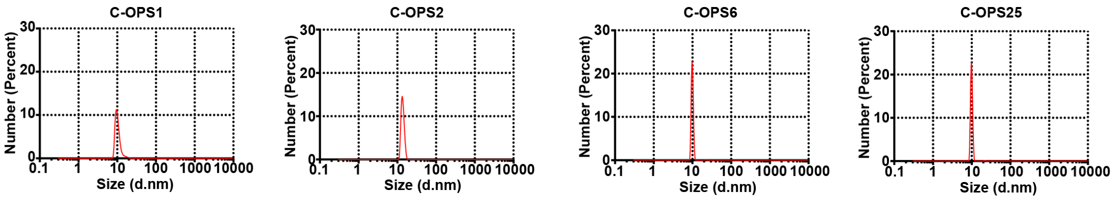

Figure S2. Dynamic light scattering analysis of C-OPS$_{ECO1}$, C-OPS$_{ECO2}$, C-OPS$_{ECO6}$ and C-OPS$_{ECO25}$

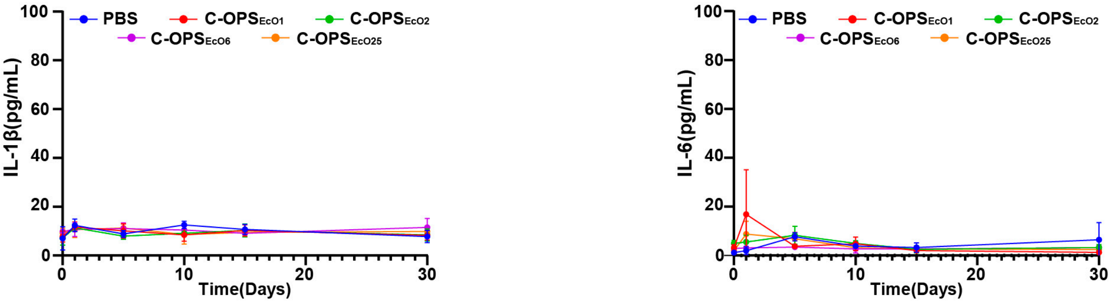

Figure S3. Changes in serum TNF-α and IL-6 levels in mice (n = 3) following administration of 5×the candidate bioconjugate vaccine during the safety evaluation observation period.

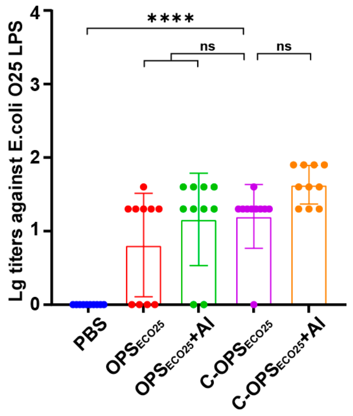

Figure S4. On the 7th day after post-immunization with C-OPS$_{ECO25}$(2.5 μg OPS$_{ECO25}$ per mouse), the IgG titer against the LPS of *E. coli* O25 was measured in serum.

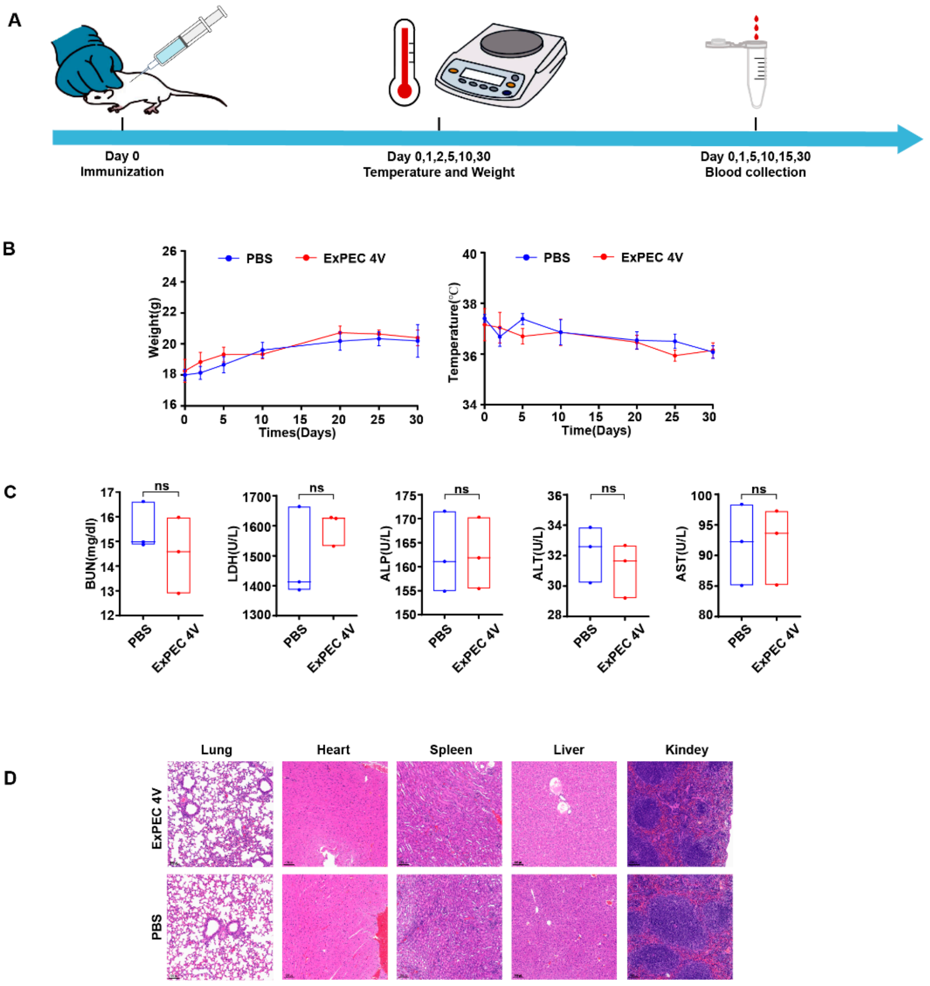

Figure S5. Safety evaluation ExPEC 4V (n = 3). (A) Overall procedure of vaccine safety evaluation. (B) Body temperature and body weight changes of mice during the

observation period. (C) Biochemical indices (including ALT, AST, BUN, LDH, and ALP) in the serum 30 days after immunization. (D) HE staining analysis of mouse lung, heart, spleen, liver and kindey.

Figure S6 The whole blot (uncropped blots) of Figure 1C.

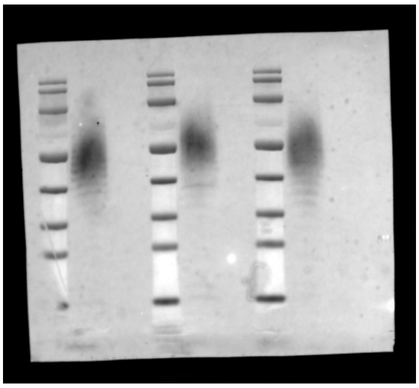

Analysis of the whole cell lysates of W3110/pBBR-O1 by anti-*E. coli* O1 antibody. From left to right, lane 4 is a protein ladder - from top to bottom - 140, 115, 80, 65, 50, 40, 30, 25, 15, 10 kDa. Lane 5 is W3110 containing the plasmid pBBR-O1 with IPTG induction, and the integrated density of bands was 43,919.110. Lane 6 is W3110 WT, and the integrated density of bands was 30,226.583. The additional lanes represent experimental replicates that were not utilized in the article.

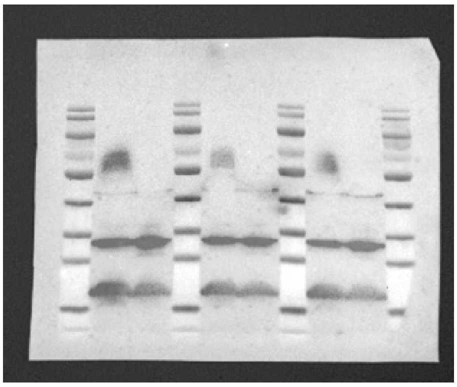

Analysis of the whole cell lysates of W3110/pBBR-O2 by anti-*E. coli* O2 antibody. From left to right, lane 7 is a protein ladder - from top to bottom - 140, 115, 80, 65, 50, 40, 30, 25, 15, 10 kDa. Lane 8 is W3110 containing the plasmid pBBR-O2 with IPTG induction, and the integrated density of bands was 23,529.860. Lane 9 is W3110 WT, and the integrated density of bands was 21,8652.535. The additional lanes represent experimental replicates that were not utilized in the article.

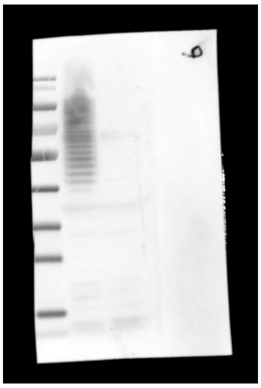

Analysis of the whole cell lysates of W3110/pBBR-O6 by anti-*E. coli* O6 antibody. From left to right, lane 1 is a protein ladder - from top to bottom - 140, 115, 80, 65, 50, 40, 30, 25, 15, 10 kDa. Lane 2 is W3110 containing the plasmid pBBR-O6 with IPTG induction, and the integrated density of bands was 10,6425.151. Lane 3 is W3110 WT, and the integrated density of bands was 66,730.678.

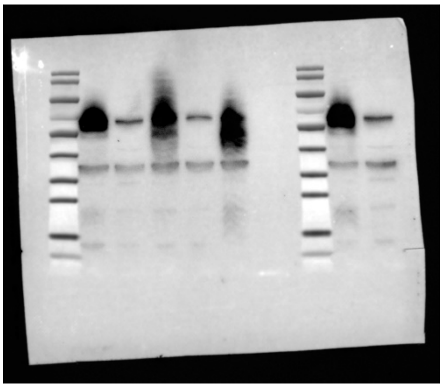

Analysis of the whole cell lysates of W3110/pBBR-O25 by anti-E. coli O25 antibody. From left to right, lane 1 is a protein ladder - from top to bottom - 140, 115, 80, 65, 50, 40, 30, 25, 15, 10 kDa. Lane 2 is W3110 containing the plasmid pBBR-O25 with IPTG induction, and the integrated density of bands was 69,553.591. Lane 3 is W3110 WT, and the integrated density of bands was 20,639.492. Other lanes were not relevant to this study.

Figure S7 The whole blot (uncropped blots) of Figure 2A.

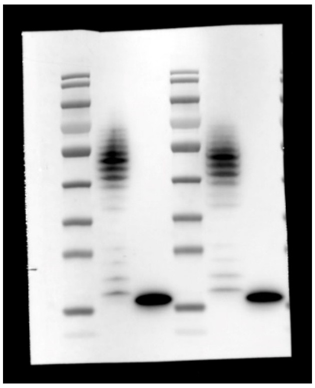

Analysis of the whole cell lysates of WdlO-d01/pET32a-*pglL*-CTB4573/pBBR-O1 by HRP-conjugated anti-6×His tag antibodies. From left to right, lane 1 is a protein ladder - from top to bottom - 140, 115, 80, 65, 50, 40, 30, 25, 15, 10 kDa. Lane 2 is WdlO-d01 containing the plasmid pET32a-*pglL*-CTB4573 and pBBR-O1 with IPTG induction, and the integrated density of bands was 48,000.663. Lane 3 is WdlO-d01 containing the plasmid pET32a-*pglL*-CTB4573, and the integrated density of bands was 21,733.773. The additional lanes represent experimental replicates that were not utilized in the article.

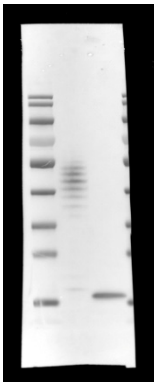

Analysis of the whole cell lysates of WdlO-d01/pET32a-*pglL*-CTB4573/pBBR-O2 by HRP-conjugated anti-6×His tag antibodies. From left to right, lane 1 is a protein ladder - from top to bottom - 140, 115, 80, 65, 50, 40, 30, 25, 15, 10 kDa. Lane 2 is WdlO-d01 containing the plasmid pET32a-*pglL*-CTB4573 and pBBR-O2 with IPTG induction, and the integrated density of bands was 43,191.683. Lane 3 is WdlO-d01 containing the plasmid pET32a-*pglL*-CTB4573, and the integrated density of bands was 12,178.397.

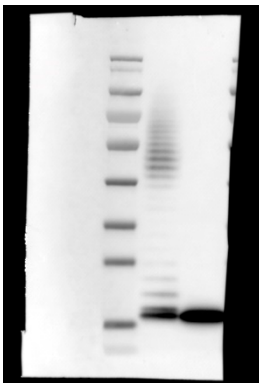

Analysis of the whole cell lysates of WdlO-d01/pET32a-*pglL*-CTB4573/pBBR-O6 by HRP-conjugated anti-6×His tag antibodies. From left to right, lane 1 is a protein ladder - from top to bottom - 140, 115, 80, 65, 50, 40, 30, 25, 15, 10 kDa. Lane 2 is WdlO-d01 containing the plasmid pET32a-*pglL*-CTB4573 and pBBR-O6 with IPTG induction, and the integrated density of bands was 27,093.408. Lane 3 is WdlO-d01 containing the plasmid pET32a-*pglL*-CTB4573, and the integrated density of bands was 14,402.225.

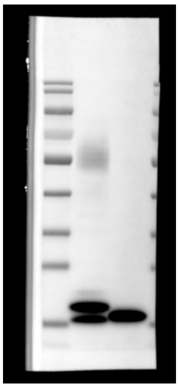

Analysis of the whole cell lysates of WdlO-d01/pET32a-*pglL*-CTB4573/pBBR-O25 by HRP-conjugated anti-6×His tag antibodies. From left to right, lane 1 is a protein ladder - from top to bottom - 140, 115, 80, 65, 50, 40, 30, 25, 15, 10 kDa. Lane 2 is WdlO-d01 containing the plasmid pET32a-*pglL*-CTB4573 and pBBR-O25 with IPTG induction, and the integrated density of bands was 32,362.889. Lane 3 is WdlO-d01 containing the plasmid pET32a-*pglL*-CTB4573, and the integrated density of bands was 20,510.894.

Figure S8 The whole blot (uncropped blots) of Figure 2B.

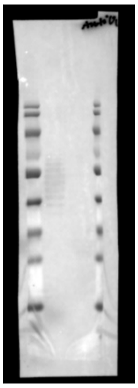

Analysis of C-OPS$_{ECO1}$ by anti-*E. coli* O1 antibody. From left to right, lane 1 is a protein ladder - from top to bottom - 140, 115, 80, 65, 50, 40, 30, 25, 15, 10 kDa. Lane 2 is C-OPS$_{ECO1}$, and the integrated density of bands was 73,633.799.

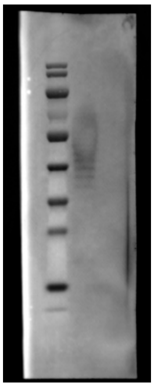

Analysis of C-OPS$_{ECO2}$ by anti-*E. coli* O2 antibody. From left to right, lane 1 is a protein ladder - from top to bottom - 140, 115, 80, 65, 50, 40, 30, 25, 15, 10 kDa. Lane 2 is C-OPS$_{ECO2}$, and the integrated density of bands was 62,415.725.

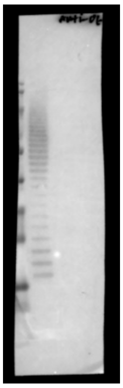

Analysis of C-OPS$_{ECO6}$ by anti-*E. coli* O6 antibody. From left to right, lane 1 is a protein ladder - from top to bottom - 140, 115, 80, 65, 50, 40, 30, 25, 15, 10 kDa. Lane 2 is C-OPS$_{ECO6}$, and the integrated density of bands was 78,075.929.

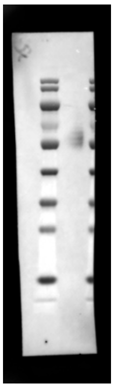

Analysis of C-OPS$_{ECO25}$ by anti-*E. coli* O25. From left to right, lane 1 is a protein ladder - from top to bottom - 140, 115, 80, 65, 50, 40, 30, 25, 15, 10 kDa. Lane 2 is C-OPS$_{ECO25}$, and the integrated density of bands was 127,274.930.

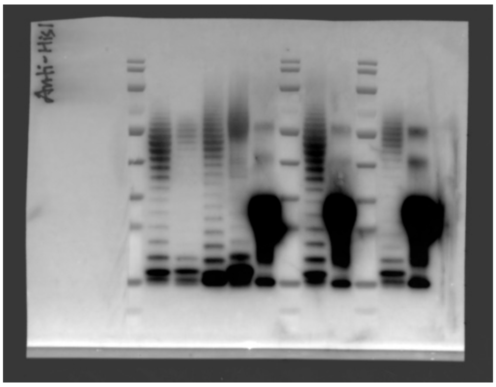

Analysis of C-OPS$_{ECO1}$, C-OPS$_{ECO2}$, C-OPS$_{ECO6}$, C-OPS$_{ECO25}$ by HRP-conjugated anti-6×His tag antibodies. From left to right, lane 1 is a protein ladder - from top to bottom - 140, 115, 80, 65, 50, 40, 30, 25, 15, 10 kDa. Lane 2 is C-OPS$_{ECO1}$, and the integrated density of bands was 50,425.666. Lane 3 is C-OPS$_{ECO2}$, and the integrated density of bands was 21,275.132. Lane 4 is C-OPS$_{ECO6}$, and the integrated density of bands was 42,504.946. Lane 5 is C-OPS$_{ECO25}$, and the integrated density of bands was 55,956.654. Other lanes were not relevant to this study.

Figure S9 Glycoproteins were detected in strains WdlO-d01 via Western blot using antibody against serum against *E. coli* O1, *E. coli* O2, *E. coli* O6 and *E. coli* O25

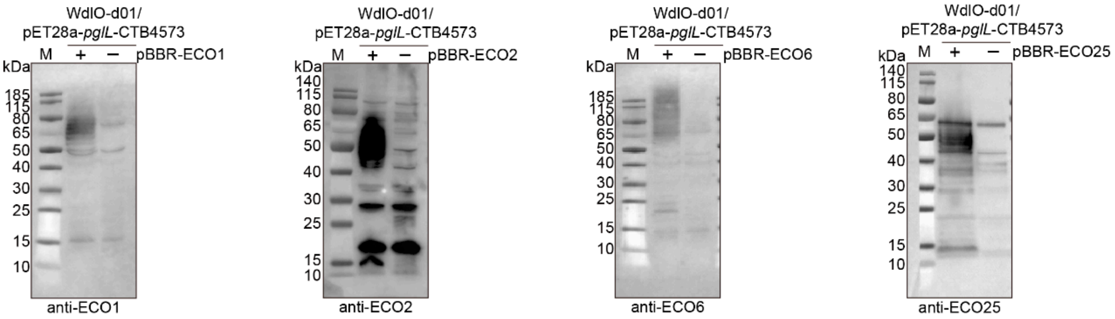

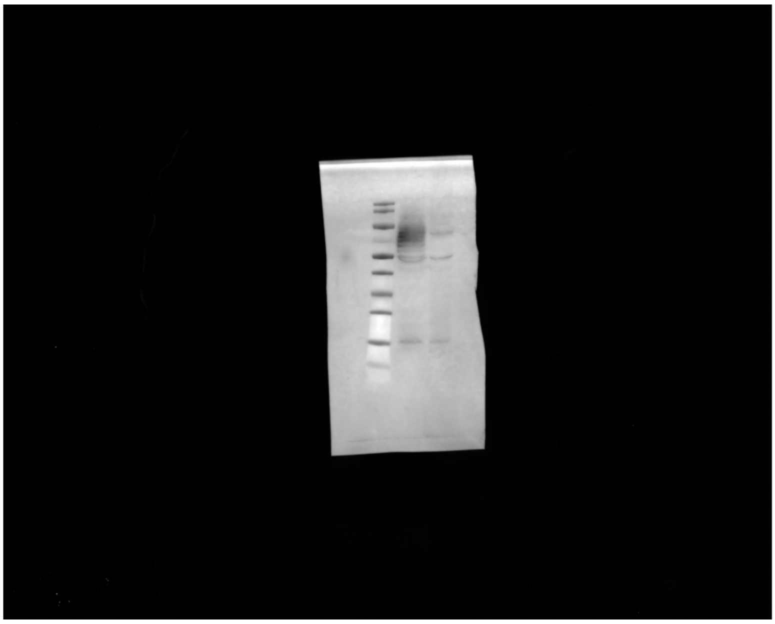

Analysis of C-OPS$_{ECO1}$ by anti-*E. coli* O1 antibody. From left to right, lane 1 is a protein ladder. Lane 2 is WdlO-d01/pET28a-*pglL*-CTB4573/pBBR-ECO1, and the integrated density of bands was 31592.141. Lane 3 is WdlO-d01/pET28a-*pglL*-CTB4573, and the integrated density of bands was 18913.137。

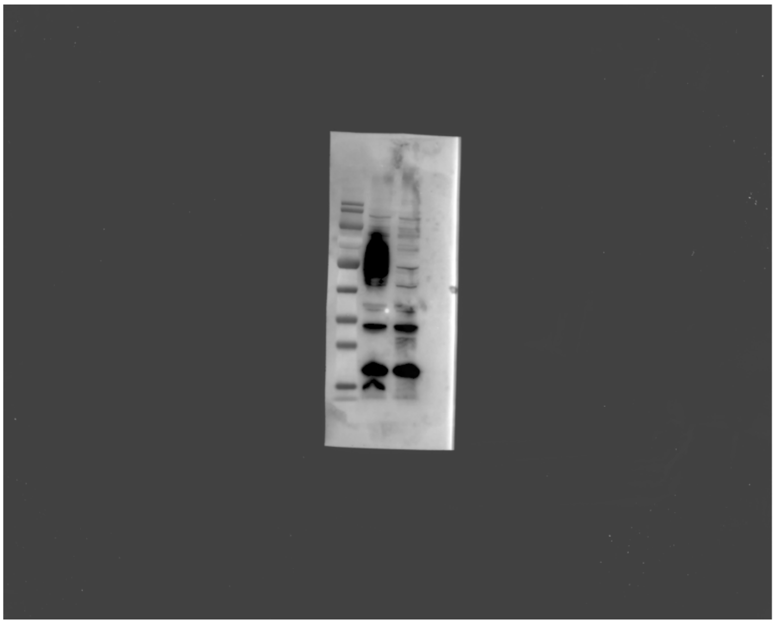

Analysis of C-OPS$_{ECO2}$ by anti-*E. coli* O2 antibody. From left to right, lane 1 is a protein ladder. Lane 2 is WdlO-d01/pET28a-*pglL*-CTB4573/pBBR-ECO2, and the integrated density of bands was 59227.872. Lane 3 is WdlO-d01/pET28a-*pglL*-CTB4573, and the integrated density of bands was 40522.479.

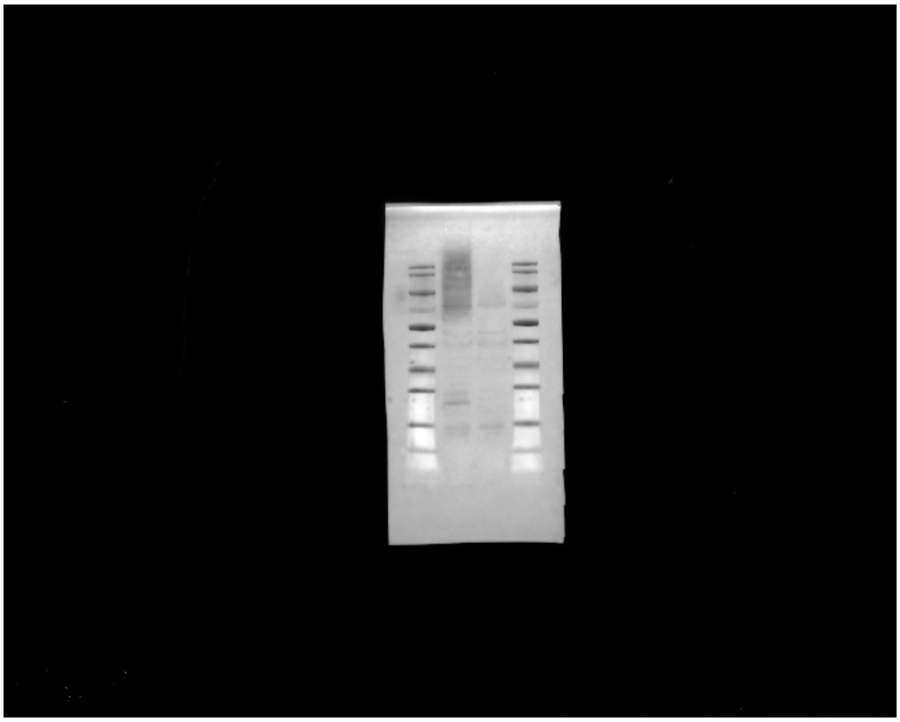

Analysis of C-OPS$_{ECO6}$ by anti-*E. coli* O6 antibody. From left to right, lane 1 is a protein ladder. Lane 2 is WdlO-d01/pET28a-*pglL*-CTB4573/pBBR-ECO6, and the integrated density of bands was 221380.374. Lane 3 is WdlO-d01/pET28a-*pglL*-CTB4573, and the integrated density of bands was 221226.859。

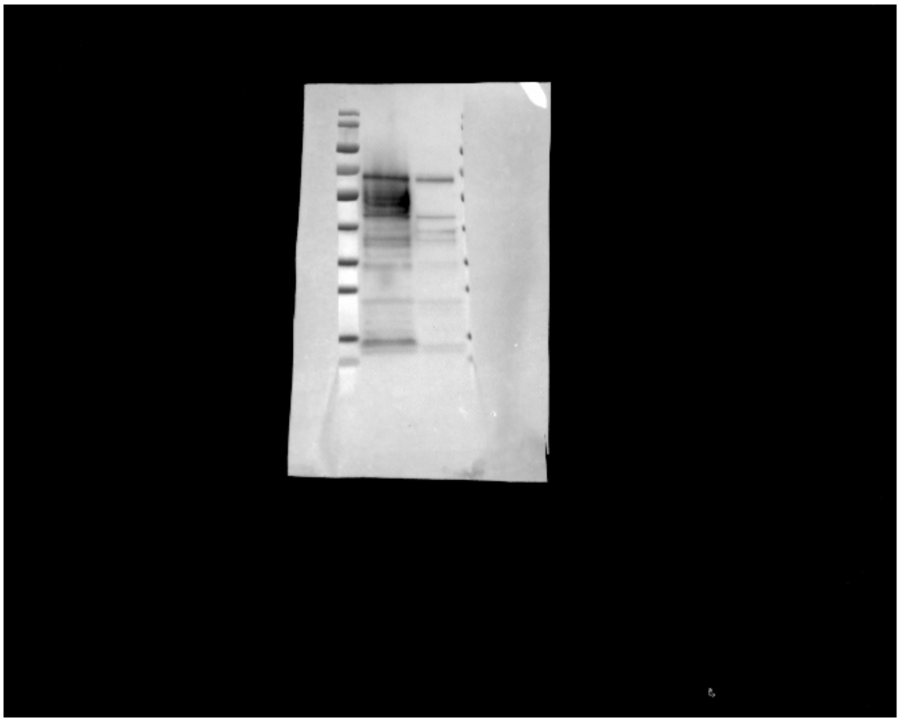

Analysis of C-OPS$_{ECO25}$ by anti-*E. coli* O25 antibody. From left to right, lane 1 is a protein ladder. Lane 2 is WdlO-d01/pET28a-*pglL*-CTB4573/pBBR-ECO25, and the integrated density of bands was 221379.864. Lane 3 is WdlO-d01/pET28a-*pglL*-CTB4573, and the integrated density of bands was 221239.710.

the sequences of pBBR

ctcgggccgtctcttgggcttgatcggccttcttgcgcatctcacgcgctcctgcggcggcctgtagggcaggctcataccc
ctgccgaaccgcttttgtcagccggtcggccacggcttccggcgtctcaacgcgctttgagattcccagcttttcggccaatc
cctgcggtgcataggcgcgtggctcgaccgcttgcgggctgatggtgacgtggcccactggtggccgctccagggcctc
gtagaacgcctgaatgcgcgtgtgacgtgccttgctgccctcgatgccccgttgcagccctagatcggccacagcggccg
caaacgtggtctggtcgcgggtcatctgcgctttgttgccgatgaactccttggccgacagcctgccgtcctgcgtcagcgg
caccacgaacgcggtcatgtgcgggctggtttcgtcacggtggatgctggccgtcacgatgcgatccgccccgtacttgtc
cgccagccacttgtgcgccttctcgaagaacgccgcctgctgttcttggctggccgacttccaccattccgggctggccgtc
atgacgtactcgaccgccaacacagcgtccttgcgccgcttctctggcagcaactcgcgcagtcggcccatcgcttcatcg
gtgctgctggccgcccagtgctcgttctctggcgtcctgctggcgtcagcgttgggcgtctcgcgctcgcggtaggcgtgc
ttgagactggccgccacgttgcccattttcgccagcttcttgcatcgcatgatcgcgtatgccgccatgcctgcccctcccttt
ggtgtccaaccggctcgacgggggcagcgcaaggcggtgcctccggcgggccactcaatgcttgagtatactcactaga
ctttgcttcgcaaagtcgtgaccgcctacggcggctgcggcgccctacgggcttgctctccgggcttcgccctgcgcggtc
gctgcgctcccttgccagcccgtggatatgtggacgatggccgcgagcggccaccggctggctcgcttcgctcggcccgt
ggacaaccctgctggacaagctgatggacaggctgcgcctgcccacgagcttgaccacagggattgcccaccggctacc
cagccttcgaccacatacccaccggctccaactgcgcggcctgcggccttgccccatcaatttttttaatttctctggggaaa
agcctccggcctgcggcctgcgcgcttcgcttgccggttggacaccaagtggaaggcgggtcaaggctcgcgcagcga
ccgcgcagcggcttggccttgacgcgcctggaacgacccaagcctatgcgagtgggggcagtcgaagggcgaagccc
gcccgcctgcccccccgagcctcacggcggcgagtgcgggggttccaagggggcagcgccaccttgggcaaggccga
aggccgcgcagtcgatcaacaagccccggaggggccactttttgccggagggggagccgcgccgaaggcgtggggg
aaccccgcaggggtgcccttctttgggcaccaaagaactagatatagggcgaaatgcgaaagacttaaaaatcaacaactt
aaaaaagggggggtacgcaacagctcattgcggcaccccccgcaatagctcattgcgtaggttaaagaaaatctgtaattga
ctgccacttttacgcaacgcataattgttgtcgcgctgccgaaaagttgcagctgattgcgcatggtgccgcaaccgtgcgg
cacccctaccgcatggagataagcatggccacgcagtccagagaaatcggcattcaagccaagaacaagcccggtcact
gggtgcaaacggaacgcaaagcgcatgaggcgtgggccgggcttattgcgaggaaacccacggcggcaatgctgctg
catcacctcgtggcgcagatgggccaccagaacgccgtggtggtcagccagaagacactttccaagctcatcggacgttc
tttgcggacggtccaatacgcagtcaaggacttggtggccgagcgctggatctccgtcgtgaagctcaacggccccggca
ccgtgtcggcctacgtggtcaatgaccgcgtggcgtggggccagccccgcgaccagttgcgcctgtcggtgttcagtgcc
gccgtggtggttgatcacgacgaccaggacgaatcgctgttggggcatggcgacctgcgccgcatcccgaccctgtatcc
gggcgagcagcaactaccgaccggccccggcgaggagccgcccagccagcccggcattccgggcatggaaccagac
ctgccagccttgaccgaaacggaggaatgggaacggcgcgggcagcagcgcctgccgatgcccgatgagccgtgtttt
ctggacgatggcgagccgttggagccgccgacacgggtcacgctgccgcgccggtagcacttgggttgcgcagcaacc
cgtaagtgcgctgttccagactatcggctgtagccgcctcgccgccctataccttgtctgcctccccgcgttgcgtcgcggt
gcatggagccgggccacctcgacctgaatggaagccggcggcacctcgctaacggattcaccgtttttatcaggctctgg
gaggcagaataaatgatcatatcgtcaattattacctccacggggagagcctgagcaaactggcctcaggcatttgagaag
cacacggtcacactgcttccggtagtcaataaaccggtaaaccagcaatagacataagcggctatttaacgaccctgccct

gaaccgacgaccgggtcgaatttgctttcgaatttctgccattcatccgcttattatcacttattcaggcgtagcaaccaggcgt
ttaagggcaccaataactgccttaaaaaaattacgccccgccctgccactcatcgcagtacggcctattggttaaaaaatga
gctgatttaacaaaaatttaacgcgaattttaacaaaatattaacgcttacaatttccattcgccattcaggctgcgcaactgttg
ggaagggcgatcggtgcgggcctcttcgctattacgccagctggcgaaagggggatgtgctgcaaggcgattaagttgg
gtaacgccagggttttcccagtcacgacgttgtaaaacgacggccagtgagcgcgcgtaatacgactcactatagggcga
attggagctccaccgcggtggcggccgctctagaactagtggatcccccgggctgcaggaattcgatatcaagcttatcga
taccgtcgacctcgagggggggcccggtacccagcttttgttccctttagtgagggttaattgcgcgcttggcgtaatcatgg
tcatagctgtttcctgtgtgaaattgttatccgctcacaattccacacaacatacgagccggaagcataaagtgtaaagcctgg
ggtgcctaatgagtgagctaactcacattaattgcgttgcgctcactgcccgctttccagtcgggaaacctgtcgtgccagct
gcattaatgaatcggccaacgcgcggggagaggcggtttgcgtattgggcgcatgcataaaaactgttgtaattcattaagc
attctgccgacatggaagccatcacaaacggcatgatgaacctgaatcgccagcggcatcagcaccttgtcgccttgcgta
taatatttgcccatgggggtgggcgaagaactccagcatgagatccccgcgctggaggatcatccagccggcgtcccgg
aaaacgattccgaagcccaacctttcatagaaggcggcggtggaatcgaaatctcgtgatggcaggttgggcgtcgcttgg
tcggtcatttcgaaccccagagtcccgctcagaagaactcgtcaagaaggcgatagaaggcgatgcgctgcgaatcggg
agcggcgataccgtaaagcacgaggaagcggtcagcccattcgccgccaagctcttcagcaatatcacgggtagccaac
gctatgtcctgatagcggtccgccacacccagccggccacagtcgatgaatccagaaaagcggccattttccaccatgata
ttcggcaagcaggcatcgccatgggtcacgacgagatcctcgccgtcgggcatgcgcgccttgagcctggcgaacagtt
cggctggcgcgagcccctgatgctcttcgtccagatcatcctgatcgacaagaccggcttccatccgagtacgtgctcgctc
gatgcgatgtttcgcttggtggtcgaatgggcaggtagccggatcaagcgtatgcagccgccgcattgcatcagccatgat
ggatactttctcggcaggagcaaggtgagatgacaggagatcctgccccggcacttcgcccaatagcagccagtcccttc
ccgcttcagtgacaacgtcgagcacagctgcgcaaggaacgcccgtcgtggccagccacgatagccgcgctgcctcgtc
ctgcagttcattcagggcaccggacaggtcggtcttgacaaaaagaaccgggcgcccctgcgctgacagccggaacacg
gcggcatcagagcagccgattgtctgttgtgcccagtcatagccgaatagcctctccacccaagcggccggagaacctgc
gtgcaatccatcttgttcaatcatgcgaaacgatcctcatcctgtctcttgatcagatcttgatcccctgcgccatcagatccttg
gcggcaagaaagccatccagtttactttgcagggcttcccaaccttaccagagggcgccccagctggcaattccggttcgc
ttgctgtccataaaaccgcccagtctagctatcgccatgtaagcccactgcaagctacctgctttctctttgcgcttgcgttttcc
cttgtccagatagcccagtagctgacattcatcccaggtggcacttttcggggaaatgtgcgcgcccgcgttcctgctggcg
ctgggcctgtttctggcgctggacttcccgctgttccgtcagcagcttttcgcccacggccttgatgatcgcggcggccttgg
cctgcatatcccgattcaacggccccagggcgtccagaacgggcttcaggcgctcccgaaggt

pBBR-O1:

accttcgggagcgcctgaagcccgttctggacgccctggggccgttgaatcgggatatgcaggccaaggccgccgcgat
catcaaggccgtgggcgaaaagctgctgacggaacagcgggaagtccagcgccagaaacaggcccagcgccagcag
gaacgcgggcgcgcacatttccccgaaaagtgccacctgggatgaatgtcagctactgggctatctggacaagggaaaa
cgcaagcgcaaagagaaagcaggtagcttgcagtgggcttacatggcgatagctagactgggcggttttatggacagcaa
gcgaaccggaattgccagctggggcgccctctggtaaggttgggaagccctgcaaagtaaactggatggctttcttgccg
ccaaggatctgatggcgcaggggatcaagatctgatcaagagacaggatgaggatcgtttcgcatgattgaacaagatgg
attgcacgcaggttctccggccgcttgggtggagaggctattcggctatgactgggcacaacagacaatcggctgctctga
tgccgccgtgttccggctgtcagcgcaggggcgcccggttctttttgtcaagaccgacctgtccggtgccctgaatgaactg
caggacgaggcagcgcggctatcgtggctggccacgacgggcgttccttgcgcagctgtgctcgacgttgtcactgaag
cgggaagggactggctgctattgggcgaagtgccggggcaggatctcctgtcatctcaccttgctcctgccgagaaagtat
ccatcatggctgatgcaatgcggcggctgcatacgcttgatccggctacctgcccattcgaccaccaagcgaaacatcgca
tcgagcgagcacgtactcggatggaagccggtcttgtcgatcaggatgatctggacgaagagcatcaggggctcgcgcc
agccgaactgttcgccaggctcaaggcgcgcatgcccgacggcgaggatctcgtcgtgacccatggcgatgcctgcttg
ccgaatatcatggtggaaaatggccgcttttctggattcatcgactgtggccggctgggtgtggcggaccgctatcaggaca

tagcgttggctacccgtgatattgctgaagagcttggcggcgaatgggctgaccgcttcctcgtgctttacggtatcgccgct
cccgattcgcagcgcatcgccttctatcgccttcttgacgagttcttctgagcgggactctggggttcgaaatgaccgaccaa
gcgacgcccaacctgccatcacgagatttcgattccaccgccgccttctatgaaaggttgggcttcggaatcgttttccggg
acgccggctggatgatcctccagcgcggggatctcatgctggagttcttcgcccaccccc atgggcaaatattatacgcaa
ggcgacaaggtgctgatgccgctggcgattcaggttcatcatgccgtttgtgatggcttccatgtcggcagaatgcttaatga
attacaacagtttttatgcatgcgcccaatacgcaaaccgcctctccccgcgcgttggccgattcattaatgcagctggcacg
acaggtttcccgactggaaagcgggcagtgagcgcaacgcaattaatgtgagttagctcactcattaggcaccccaggctt
tacactttatgcttccggctcgtatgttgtgtggaattgtgagcggataacaatttcacacaggaaacagctatggtgaagata
cttgttactgggggcgcaggatttattggttctgctgtagttcgtcacattataaataatacgcaggatagtgttgttaatgtcgat
aaattaacgtacgccggaaacctggaatcacttgctgatgtttctgactctgaacgctatgtttttgaacatgcggatatttgcg
atgctgctgcaatggcgcggatttttgctcagcatcagccggatgcagtgatgcacctggctgctgaaagccatgtggatcg
ttcaattacaggccctgcggcatttattgaaaccaatattgttggtacttatgtccttttggaagcggctcgcaattactggtctg
ctcttgatggcgacaagaaaaatagcttccgttttcatcatatttctactgacgaagtctatggtgatttgcctcatcctgacgaa
gtaaataataaagaacaattacccctctttactgagacgacagcttacgcgcctagtagtccttattccgcatcaaaagcatcc
agcgatcatttagtccgcgcgtggaaacgtacctatggtttaccgactattgtgactaactgttcgaataactacggtccttatc
actttccggaaaaattgattccactagtaattcttaatgctctggaaggtaaggcattacctatttatggcaaaggggatcaaat
tcgtgactggctgtatgttgaagatcatgcgcgtgcgttatataccgtagttactgaaggtcaagcgggtgaaacctataacat
tggcggacacaacgaaaagaaaaacatcgatgttgtgctgactatttgtgatttgttggacgagatagtcccgaaagagaaa
tcttatcgtgagcaaattacttatgttgctgatcgcccagggcatgatcgccgttatgcgattgatgctgagaagattggtcgc
gaattgggatggaaaccacaggaaacgtttgagagtgggattcgtaaaacggtggaatggtatttggctaatgcaaaatgg
gttgataatgtgaaaagtggtgcctatcaatcgtggattgaacagaactatgagggccgccagtaatgaatatcctcctttttg
gcaaaacagggcaggtaggttgggaactacagcgtgctctggcacctctgggtaatttgattgctcttgatgttcactccact
gattactgtggtgattttagtaaccctgaaggtgtggctgaaacagtcaaaagaattcgacctgatgttattgttaatgctgcgg
ctcacaccgcagtagataaggctgagtcagaacccgaatttgcacaattactcaatgcgactagcgttgaatcaattgcaaa
agcggcaaatgaagttggggcttgggtaattcattactcaactgactacgtattccctggaaatggcgacacgccatggctg
gagatggatgcaaccgcaccgctaaatgtttacggtgaaaccaagttagctggagaaaaagcattacaagagcattgtgcg
aagcacctaattttccgtaccagctgggtctatgcaggtaaaggaaataatttcgccaaaacgatgttgcgtctggcaaaaga
gcgtgaagaactagccgttattaatgatcagtttggtgcgccaacaggtgctgaactgctggctgattgtacggcacatgcc
attcgtgtcgcactgaataaaccggatgtcgcaggcttgtaccatttggtagccagtggtaccacaacctggtacgattatgc
tgcgctggtttttgaagaggcgcgcaatgcaggcattcctcttgcactcaacaagctcaacgcagtaccaacaactgcctat
cctacaccagctcgtcgtccacataactctcgccttaatacagaaaaatttcagcagaattttgcgcttgtattgcctgactggc
aggttggtgtgaaacgcatgctcaacgaattatttacgactacagcaatttaatagtttttgcatcttgttcgtgatggtggagca
agatgaattaaaaggaatgatgaaatgaaaacgcgtaaaggtattattttagcgggtggttctggtactcgtctttatcctgtga
ctatggtcgtcagtaaacagctattacctatatatgataaaccgatgatctattatccgctttctacactgatgttagcgggtattc
gcgatattctgattattagtacgccacaggatactcctcgttttcaacaactgctgggtgacggtagccagtggggcctgaat
cttcagtacaaagtgcaaccgagtccggatggtcttgcgcaggcatttattatcggtgaagagtttattggtggtgatgattgt
gctttggtacttggtgataatatcttctacggtcacgacctgcctaagttaatggatgccgctgttaacaaagaaagtggtgca
acggtatttgcctatcacgttaatgatcctgaacgctatggtgtcgttgagtttgataaaaacggtacggcgatcagcctggaa
gaaaaaccgctacaaccaaaaagtaattatgcggtaaccgggctttatttttatgataacgacgttgtcgaaatggcgaaaaa
tcttaagccttctgcccgcggtgaactggaaattaccgatattaaccgtatctatatggaacaagggcgtttatctgttgccatg
atggggcgtggttatgcgtggttagacacggggacacatcagagcctgattgaggcaagcaactttattgcaacaattgaa
gagcgtcaggggctgaaagtttcctgcccggaagaaattgcttaccgtaaagggtttgttgatgctgagcaggtgaaagtat
tagctgaacctctgaaaaaaaatgcttatggtcagtatctgctgaaaatgattaaaggttattaataaaatgaacgtaattaaaa
cagaaattcctgatgtactgatttttgaaccgaaagtttttggtgatgagcgtggtttctttttgagagctttaaccagaaggtttt

tgaggaagctgtaggccgcaaagttgaatttgttcaggataaccattcgaagtctagtaaaggtgttttacgcgggctgcatt
atcagttggaaccttatgcacaaggaaaattggtgcgttgcgttgtcggtgaagtttttgacgtagctgttgatattcgtaaatc
gtcatcgacttttggcaaatgggttggggtgaatttatctgctgagaataagcggcaattgtggattcctgagggatttgcaca
tggtttttagtgctgagtgagacggcggagtttttgtataagacgacaaattattatcatcctcagagtgatagaggaataaaa
tgggatgatccaagtatcaatatttcatggccagtcgattcacaagtgctgctatcagctaaagataataagcatcctccattaa
caaagattgaaatgtatagttaagatcacgataaatcttggaagggttgcaaaattgaataaaatagtgagcaaaagtgaaat
aaggaacgtaatccacaatgctggctatatgatgattactcagatagctttatatgttgcaccattatttatactgagttatctgtta
aaaacactgggggttgcacagtttggtaattatgccttaatactatcaatcgttgcatatttacagattataacggattatggttttt
cttttagtgcaagtcgtgcgatctcacagaatagagaggacaaagaatatatatcaaaaatttatctgtcaactatgactatcaa
gttggcgatatgcgctttcttattcttattgctcatgctatttttaaatcttttgcctgtgcaagctgaattaaaacaaggaatattat
atggatatcttcttgtaataggaaatactttccaaccacaatggtttttccaaggtatcgaaaaattaaaaatcatagccctttcta
atgttatatcaagatgcgccgcgtgtttacttgtatttatctatgtgaggaatagcgaggatttacaaaaagcacttttagtacag
tcacttccattagtaatttctgcgattggattaaatatatttatattgaaatatatcaatattatttttccggaaaaaaaattatttaag
gtaattttaaaagaaggtaaggatttttttcttgcatcactttattctgttattctcaataatagtggcatttttctattagggatttttac
taatcctgttattgttggtgtatatgccgccgctgaaaagatagtcaaggccgtattgtcgctatttacaccactgacgcaagct
atatatccttataattgtcgtaagttttcactatccgtatttgacggcattgaggcagcaaaaaaaactggtataccaattataatt
ttagcatttatagctgctgttatcgttgcaattaccttacctgttgcaatcgactatcttaattttccaaaagaaacaatttttgtagg
tcaaatattaagtgcatggatctttttttggtgttcttaataatgtattcggcattcagatattgagtgcatcaggaagaagtaaaat
atatagtaggatggtattcgtatcagcgcttataacattacttttgattactctattattgcagttttgtaacgccactggagtggca
tgtgcaatattattgggtgaaatgttcttatcaatattgttacttaagcgatataaaaaaataatttaaggaatagttatgaagaag
ttattattagtgttcggtactaggcctgaagcaataaagatggcctctatcattgaattattaaaaaaagattgtagattcgaatat
aaaatatgtgtgacaggccaacataaagagatgcttgatcaagttatgcaagtatttgatgttaaacctgattataatttacgga
ttatgcagcctgggcaaacattagtatctgtagcaacaaatatactctcacggttaagtgaagttttaattatagaaaagccag
atattatacttgtgcatggggatacaacgactacccttgctgctactttagctgggtattaccaccaaataaaagtttgtcatgtg
gaagcaggattaagaacaggggatatttactctccttggcctgaagagggcaatcgtaaagttacaggggcattagcatgta
ttcatttcgccccaacagagagatcaaaagataatctcctgagggaggggtcaaagtaaataatatatttgtaacgggtaata
ccgtcatcgactctttatttattgcaaaagatatcatagataatgaccctaatataaagaacgctttacataataaatttaattttctt
gataaaagccgacgagtagtacttataacaggtcatcgaagagaaaatttcgggaaaggttttgaagatatatgctttgcaat
aaaggaattagctttcatttatcctaatgtagattttatttatccggtgcatcttaatcccaatgtaatggaaccagtacatcgtata
ttagataatatatgtaatatttaccttattgagcccttggattatttgccttttgtttatttaatgaatgagtcatatttaatattgactga
ttcaggggggatacaagaagaagcgccttcgttaggtaaaccggttttggttatgcgtgatactactgaacgccctgaggcgg
ttgaggctggtactgttgtattagtggggacttctaagataaaaatagtaaataaagtaacggagctattaaacaatgctgatat
ctacaatgctatgtctctgttacataatccatatggcgatggaacagctgctcaaaaaattcttaatgtgctcgcccaagagcta
atttaatttaagctaaaaatatgttattaattattgctgattatccaaacgaaatgaatatgcgcgagggagctatgcaacgaata
gatgcgatagactctctcattcgagatcgcaagcgagtgtatttgaatatttcattcaaaaagcatctagttcgctcaaatagttc
ctttaataatgttatagttgaaaatctaaatgcaattattcacagaaacatcataaaacagtacatgcaaaaatcaacaactatat
atgttcattctgtttataatttattaaaggttataacgctcattgatctaaaaaaaacaattcttgatatacatggtgttgtaccggaa
gaacttttggcagataataaaaaattacttagtaaagtatataacatggtggaaaaaaaaggtgtccttggatgcaaaaaatta
atacacgtcagtacagaaatgcaaaaacactatgaagcaaaatatggagtaaacttggctgaaaggtcaatagtgctcccg
atttttgaatataaaaatataacccaatcgcaaaacaaatggacagaaaataaaatacgaagtatctatcttggaggattacaa
acatggcaaaatattgataaaatgattcaaatttgtgatgacacagtgataaacaatgaagcaggtaagtatgaattcaactttt
tcatcccacagagtaacttggaagggtttatagataaatattcgttaaaattacataatatcaatgctaatgcatctacgctatca
cgtgatgaagtaattccctttctaaaagaatgtcatattggttttgtattgcgcgatgatataatagtaaacagagttgcgtgccc
tacaaaattggttgaatatttagagtgtggtgtcgttccagttgtgctctccccacttataggtgatttttattcgatgggatatcaa

tacattactacagaggaaatggctaacagaagtataagtttgttggatcttgaaaaaatggctgcacataatttacaaattttga
cttcttatcagaagagaacctacaaggcacagaaagaacttattgctcaactgtgctgaatttttacatatataaaattatgtaa
gcatatcgctggtcaggtaattgtatgcgtatcaaatataaagataacggttatatattatgttttctattatgtttcattttgagcta
cttagttttactcaaatctgactactttcctgctgattttctgccatatacagaaatatacgatgggacatacggagaaatcaata
atattgagcctgccttttatatttaacacggttgtttcattatttaaatttcccctatatatttttgcaatgttagtttgtgccttatgttt
aagttggaaaataaaatatgcaagaaaaataattaaagatagttatatatatttattcttgtatgtatatgtatcattttatgtgttttt
gcatgaaatgactcaattgcgcatagcaattgcagtcactatgtgctatgtgtcggtttattattacttttataaaaattgtattaaa
catgcactgccatggatggtgttggctattttgtttcattacagcgccttgcttttatttatgtcattatttatatacagttataggag
gttattaatagtaattatagggtttgtaatatgtatgagctttttaaacgtgtatgcagatacaattgcactatatttgccaaatgaa
aaaatagtaaattatttatatagtatttcatcatcattagacaatagaaatgatttggcaatattcaacctgaataatataatattttta
tcaatatttattttgatcttttatcttagccgatatataaaattaaatgataatgaggcgaagtttattaagtatgtgcaatgttcagg
aatattagccttttgtattttctttctggctagtggagtcccggtcattgcttatcgaactgcagagttgctgcgaatattttatccg
atggctttagtattaatcctttcgcatataaaaaataataatatgcgttattttattgcagtcattatagttatcctttcaggcttaatgt
tgtttataacactaagggctgtatcaatagttggtcaaggattataaaatgaatgttgctattttgttgtctacgtataatggcgaa
aaatatttagaggaacaactggattcattgctgcttcaaagttatcaggattttgtagtgtatatccgtgatgacggatcatctga
tagaactgtaaatataataaaccaatacgtaatgaaagataacagatttattaacgtgggtaattcagaaaatcttggttgtgct
gcttcgtttattaatttattaagaaatgcttcagccgatatttatatgttttgtgaccaagatgattattggcttccgaataaattaca
gcgtgctgtggattatttttcggctattgatcctttacaacctaccttgtatcattgcgatctaagcgttgttgatgaaaaacttaat
attatacaaaattcatttttgcagcatcagaaaatgtcagcgtatgattcaatgagaaaaaaataatcttttcatacaaaattttgttg
ttggttgttcatgtgctgttaatgcttcacttgcggaatttgttctttcgcgaattggagagcagcatgtaaaaatgatagctatgc
atgactggtggttagccgtgactgcaaaactttttggtcgaatccattttgataatactcaaacgattctttatcgacaacatcag
ggcaatgtattaggtgcaaaatcatcaggtatgatgcgttttattcgattaggattaaatgggcaagggatttcgcgagtagtat
cttttagaaaaaaagtttgtgcgcaaaataagcttcttttagatgtctatgataaagagttaaatcttgagcaaaaaaaaatctatc
aggcttgtaattgagggccttaaagagaactcttcaattgctgaccttttaaaatgtttctatcatggtagctatatgcaaggtttt
aaacgtaatcttgccttaatatattcagttctttacacaaaaaaaaagaagatagtgtatccttatgaaaaaaattgctattatcggt
actgttggcataccagcatcatatggcggatttgaaacattagttgaaaatttaacaagatacaattcctcgggagttgaatata
atgtttttgttcatcgtttcactacaaatcccaccaaaaaaaacataatggggcccgtttaatttatattccgcttaaagccaatg
gatggcagagcattgcgtatgacataatttcgttagcatattctatttttttgaagcctgatgtgattctgattttaggggtttctggt
tgttcatttttgcctttcttcaaactcttaacacgcgctaagtttattactaatattgatggcttggaatggcgaagagataaatgg
aattcaaaagtgaaacgtttcttaaaattttcagaaaaaatcgcagttcaatattcggatgtcgttattacggataatgaggcaat
ttctgagtacgtttttaacgagtataataaagatagccgagttattgcctatggaggggatcatgcatggttaaatactgaggat
gtatttacaacaagaaattataaaagcgattactacctttctgtatgtcgtatcgaacccgaaaacaatgtagaattaattttaaa
aacattttcaaagctaaaatataaaataaaatttattggaaattggaatggcagcgagtttggaaagaaacttaggctgcattat
tctaactatccaaatattgaaatgattgatccgatttatgatcttcaacaattatttcacttacgaaataattgcataggatatatac
atggtcattcggctggaggaacaaacccttctttagtcgaggcaatgcattttagtaaacctatatttgcatatgattgtaagttt
aataggtacactactgaaaatgaagcatgttatttttctaatgaatctgacctcgcagagaaaatcataatgcattgtgagctat
cattaggtgtctctggcacgaaaatgaaagaaattgctaaccagaaatacacttggagacgaatagcagaaatgtatgagg
attgctattaactctgttaaacttcaaatcttttacaatatatggcatgactataagcgcattaattgtttttcaagccgctctcgcg
gtgaccacccccctgacaggagtaaacaatgtcaaagcaacagatcggcgtcgtcggtatggcagtgatggggcgcaacc
ttgcgctcaacatcgaaagccgtggttataccgtctctattttcaaccgttcccgtgaaaagacggaagaagtgattgccgaa
aatccaggcaagaaactggttccttactatacggaggtcgacggtatcgataagcttgatatcgaattcctgcagcccgggg
gatccactagttctagagcggccgccaccgcggtggagctccaattcgccctatagtgagtcgtattacgcgcgctcactg
gccgtcgttttacaacgtcgtgactgggaaaaccctggcgttacccaacttaatcgccttgcagcacatccccctttcgccag
ctggcgtaatagcgaagaggcccgcaccgatcgcccttcccaacagttgcgcagcctgaatggcgaatggaaattgtaag

cgttaatattttgttaaaattcgcgttaaatttttgttaaatcagctcatttttaaccaataggccgtactgcgatgagtggcaggg
cggggcgtaatttttttaaggcagttattggtgcccttaaacgcctggttgctacgcctgaataagtgataataagcggatgaa
tggcagaaattcgaaagcaaattcgacccggtcgtcggttcagggcagggtcgttaaatagccgcttatgtctattgctggtt
taccggtttattgactaccggaagcagtgtgaccgtgtgcttctcaaatgcctgaggccagtttgctcaggctctccccgtgg
aggtaataattgacgatatgatcatttattctgcctcccagagcctgataaaaacggtgaatccgttagcgaggtgccgccgg
cttccattcaggtcgaggtggcccggctccatgcaccgcgacgcaacgcggggaggcagacaaggtatagggcggcga
ggcggctacagccgatagtctggaacagcgcacttacgggttgctgcgcaacccaagtgctaccggcgcggcagcgtga
cccgtgtcggcggctccaacggctcgccatcgtccagaaaacacggctcatcgggcatcggcaggcgctgctgcccgc
gccgttcccattcctccgtttcggtcaaggctggcaggtctggttccatgcccggaatgccgggctggctgggcggctcctc
gccggggccggtcggtagttgctgctcgcccggatacagggtcgggatgcggcgcaggtcgccatgccccaacagcga
ttcgtcctggtcgtcgtgatcaaccaccacggcggcactgaacaccgacaggcgcaactggtcgcggggctggccccac
gccacgcggtcattgaccacgtaggccgacacggtgccggggccgttgagcttcacgacggagatccagcgctcggcc
accaagtccttgactgcgtattggaccgtccgcaaagaacgtccgatgagcttggaaagtgtcttctggctgaccaccacg
gcgttctggtggcccatctgcgccacgaggtgatgcagcagcattgccgccgtgggtttcctcgcaataagcccggccca
cgcctcatgcgctttgcgttccgtttgcacccagtgaccgggcttgttcttggcttgaatgccgatttctctggactgcgtggcc
atgcttatctccatgcggtaggggtgccgcacggttgcggcaccatgcgcaatcagctgcaacttttcggcagcgcgacaa
caattatgcgttgcgtaaaagtggcagtcaattacagattttctttaacctacgcaatgagctattgcggggggtgccgcaatg
agctgttgcgtacccccctttttttaagttgttgatttttaagtctttcgcatttcgccctatatctagttctttggtgcccaaagaagg
gcacccctgcggggttcccccacgccttcggcgcggctcccctccggcaaaaagtggcccctccggggcttgttgatcg
actgcgcggccttcggccttgcccaaggtggcgctgcccccttggaacccccgcactcgccgccgtgaggctcggggg
gcaggcgggcgggcttcgcccttcgactgcccccactcgcataggcttgggtcgttccaggcgcgtcaaggccaagccg
ctgcgcggtcgctgcgcgagccttgacccgccttccacttggtgtccaaccggcaagcgaagcgcgcaggccgcaggc
cggaggcttttccccagagaaaattaaaaaaattgatggggcaaggccgcaggccgcgcagttggagccggtgggtatgt
ggtcgaaggctgggtagccggtgggcaatccctgtggtcaagctcgtgggcaggcgcagcctgtccatcagcttgtccag
cagggttgtccacgggccgagcgaagcgagccagccggtggccgctcgcggccatcgtccacatatccacgggctggc
aagggagcgcagcgaccgcgcagggcgaagcccggagagcaagcccgtagggcgccgcagccgccgtaggcggtc
acgactttgcgaagcaaagtctagtgagtatactcaagcattgagtggcccgccggaggcaccgccttgcgctgcccccgt
cgagccggttggacaccaaaagggaggggcaggcatggcggcatacgcgatcatgcgatgcaagaagctggcgaaaa
tgggcaacgtggcggccagtctcaagcacgcctaccgcgagcgcgagacgcccaacgctgacgccagcaggacgcca
gagaacgagcactgggcggccagcagcaccgatgaagcgatgggccgactgcgcgagttgctgccagagaagcggc
gcaaggacgctgtgttggcggtcgagtacgtcatgacggccagcccggaatggtggaagtcggccagccaagaacagc
aggcggcgttcttcgagaaggcgcacaagtggctggcggacaagtacggggcggatcgcatcgtgacggccagcatcc
accgtgacgaaaccagcccgcacatgaccgcgttcgtggtgccgctgacgcaggacggcaggctgtcggccaaggagt
tcatcggcaacaaagcgcagatgacccgcgaccagaccacgtttgcggccgctgtggccgatctagggctgcaacggg
gcatcgagggcagcaaggcacgtcacacgcgcattcaggcgttctacgaggccctggagcggccaccagtgggccac
gtcaccatcagcccgcaagcggtcgagccacgcgcctatgcaccgcagggattggccgaaaagctgggaatctcaaag
cgcgttgagacgccggaagccgtggccgaccggctgacaaaagcggttcggcaggggtatgagcctgccctacaggcc
gccgcaggagcgcgtgagatgcgcaagaaggccgatcaagcccaagagacggcccgag
pBBR-O2:
accttcgggagcgcctgaagcccgttctggacgccctggggccgttgaatcgggatatgcaggccaaggccgccgcgat
catcaaggccgtgggcgaaaagctgctgacggaacagcgggaagtccagcgccagaaacaggcccagcgccagcag
gaacgcgggcgcgcacatttccccgaaaagtgccacctgggatgaatgtcagctactgggctatctggacaagggaaaa
cgcaagcgcaaagagaaagcaggtagcttgcagtgggcttacatggcgatagctagactgggcggttttatggacagcaa
gcgaaccggaattgccagctggggcgccctctggtaaggttgggaagccctgcaaagtaaactggatggctttcttgccg

ccaaggatctgatggcgcaggggatcaagatctgatcaagagacaggatgaggatcgtttcgcatgattgaacaagatgg
attgcacgcaggttctccggccgcttgggtggagaggctattcggctatgactgggcacaacagacaatcggctgctctga
tgccgccgtgttccggctgtcagcgcaggggcgcccggttctttttgtcaagaccgacctgtccggtgccctgaatgaactg
caggacgaggcagcgcggctatcgtggctggccacgacgggcgttccttgcgcagctgtgctcgacgttgtcactgaag
cgggaagggactggctgctattgggcgaagtgccggggcaggatctcctgtcatctcaccttgctcctgccgagaaagtat
ccatcatggctgatgcaatgcggcggctgcatacgcttgatccggctacctgcccattcgaccaccaagcgaaacatcgca
tcgagcgagcacgtactcggatggaagccggtcttgtcgatcaggatgatctggacgaagagcatcaggggctcgcgcc
agccgaactgttcgccaggctcaaggcgcgcatgcccgacggcgaggatctcgtcgtgacccatggcgatgcctgcttg
ccgaatatcatggtggaaaatggccgcttttctggattcatcgactgtggccggctgggtgtggcggaccgctatcaggaca
tagcgttggctacccgtgatattgctgaagagcttggcggcgaatgggctgaccgcttcctcgtgctttacggtatcgccgct
cccgattcgcagcgcatcgccttctatcgccttcttgacgagttcttctgagcgggactctggggttcgaaatgaccgaccaa
gcgacgcccaacctgccatcacgagatttcgattccaccgccgccttctatgaaaggttgggcttcggaatcgttttccggg
acgccggctggatgatcctccagcgcggggatctcatgctggagttcttcgcccaccccccatgggcaaatattatacgcaa
ggcgacaaggtgctgatgccgctggcgattcaggttcatcatgccgtttgtgatggcttccatgtcggcagaatgcttaatga
attacaacagtttttatgcatgcgcccaatacgcaaaccgcctctccccgcgcgttggccgattcattaatgcagctggcacg
acaggtttcccgactggaaagcgggcagtgagcgcaacgcaattaatgtgagttagctcactcattaggcaccccaggctt
tacactttatgcttccggctcgtatgttgtgtggaattgtgagcggataacaatttcacacaggaaacagctatggtgaaaata
cttgttactggtggcgcaggatttattggttctgctgtagttcgtcacattataaataatacgcaggatagtgttgttaatgtcgat
aaattaacgtacgccggaaacctggaatcacttgcagatgcttctgattctgaacgctatgtttttgaacatgcggatatttgtg
atgcagctgcaatggcacggatttttgctcagcatcagccggatgcagtgatgcacctggcggctgagagccacgttgacc
gctcaataactggcccggcggcattcattgaaactaatattgtaggtacttacgtacttttagaagcagcgcgcaattattggt
ctggtctggatgatgaaaagaaaaaaaacttccgctttcatcatatttctactgatgaagtgtatggtgacttaccccatccgga
tgaagtaaatagcaatgaaacgttgccgctatttacggaaacgacagcatacgcgccaagtagtccatattctgcttctaaag
cgtcaagcgatcatttggttcgcgcatggaaacgtacttatggtttaccgacaattgtgactaattgctcgaacaactatggtc
cttatcatttcccggaaaagcttattccactggttattcttaatgcactggaaggtaaggcattacctatttatggtaaaggggat
cagatccgcgactggttgtatgtagaggatcatgctcgtgcgttatataccgtagttactgaaggtcaagcgggtgaaaccta
taacattggcggacacaacgaaaagaaaaacatcgatgttgtgctgactatttgtgatttgttggacgagatagtcccgaaag
agaaatcttatcgtgagcaaattacttatgttgctgatcgcccagggcatgatcgccgttatgcgatcgatgccgataaaatta
gccgtgaattgggctggaaaccgcaagaaacgtttgagagcgggattcgaaagacggtggaatggtacctgtccaataca
aaatgggttgataatgtgaaaagtggcgcctatcaatcgtggattgaacagaactatgaggaccgccagtaatgaatatcct
cctattcggcaaaacagggcaggtaggttgggaactacagcgtgctctggcaccgctgggtaatctgattgctcttgatgttc
actccactgattattgtggtgattttagtaatcccgaaggtgtagctgaaactgtcaaaaaaattcgccctgatgttattgttaat
gcggctgctcacactgcagtagacaaagcggaatcagaaccggaatttgcacaattacttaacgcgacaagtgtcgaagc
gattgcaaaagcagccaatgaagtcggcgcttgggttattcactactctactgactacgtatttccgggaaccggtgaaatac
cgtggcaggagacggatgctacagcacctttgaatgtttatggtgaaactaaactagccggagaaaaagcgttacagaaac
attgcgcgaagcatattattttccgtaccagctgggtctatgcaggtaaaggaaataacttcgccaaaacaatgttgcgtctgg
caaaagagcgcgaagaactggctgtgataaacgatcagtttggcgcaccaacaggtgccgaattgctggctgattgcacc
gctcatgccattcgcgtagcagtagacaaaccagaagttgctggtttgtaccatctggttgctggcggtacaacaacctggc
acgattatgccgctctggtatttgaagaagcgcgcaaagcagggattaaccttgctcttaacaaacttaacgccgtgccaac
aacggcctatcccacaccagcccgtcgaccccataactctcgcctcaatacagaaaagtttcagcagaactttgcgcttgtct
tgcctgactggcaggtgggcgtgaaacgaatgctcaacgaattatttacgactacggcaatttaacaaatttttgcatctcgct
catgatgccagagcgggatgaattaaaaggaatggtgaaatgaaaacgcgtaaaggtattattctggctggtggttccggc
actcgtctttatcctgtgacgatggcagtaagtaaacaactgctgccgatttatgataaacctatgatctattacccgctctcaa
cactgatgctggcgggtattcgcgatattcttattatcagtacgccacaggatacaccgcgtttccaacaattgttggggac

gggagccagtgggggcttaatctacagtataaagtacaaccgagtccggatggcctggcacaagcgtttatcattggtgaa
gactttattagtggcgatgattgtgcactcgtgcttggagataatatcttctatggacacgacttgccaaaattaatggacgctg
cggttaacaaagaaagcggtgcaacggtatttgcttatcacgttaatgatcctgaacgctatggtgtcgtggagtttgataata
acggtacggcaattagcctggaagaaaaaccgctggaaccaaaaagtaactatgcggttactgggctttatttctatgacaat
gacgtcgtggaaatggcgaaaaaccttaagccttctgcccgtggcgaactggaaattaccgatattaaccgtatttatatgga
acaaggacgtttgtctgtcgctatgatggggcgtggttatgcatggctggatacagggacgcatcaaagccttattgaagca
agtaacttcattgccaccattgaagagcgtcagggattaaaagtatcttgcccggaagagattgcttatcgtaaagggtttatt
gatgctgagcaggtaaaagtattagccgaaccgcttatcaagaatcaatatggtcaatatttgctgaaaatgatcagcgaata
gtatgtgggaactcaatgatggatattaaattaatctctttgcaaaaacatggggatgagcgtggtgcattaattgctcttgaag
agcaacgaaatatacctttcgaagtcaaaagaatatattacatactcgagactcttaagggagtaagacgcggatttcatgcg
cacaaggttactcgtcagttagctattgtagtcaagggagcttgtaaatttcatctggataatggtaaagaaacaaagcaggt
ggaacttaatgatccaacaattgcgttgctgatagaaccctatatatggcatgaaatgtatgattttagtgatgattgtgtgctgc
ttgtaattgcggatgatttctataaagagtctgattatatacgcaattatgatgattttattagaagagtaaattcaattgagaattc
ataagctaagtgacgtccagacaacatcaattggtgatggaacaactatctggcagtttgttgtgatactaaaaggtgctgtaa
ttggtaataattgcaatatctgtgcaaataccttgattgaaaataacgttgtaattggtaacaatgtcacagtcaaaagcggtgt
gtatatttgggatggcgttaaaatagaggataatgtttttattggtccttgtgtagcatttacaaatgataagtatcctcgctctaa
agtctatcctgatgaatttttgcacacaataatacgcaaaggagcatcaataggtgctaatgcaaccatcctgccaggaattg
aaattggtgaaaaagcaatcgttggtgcggggagtgtcgtaaccaaaaatgtaccgccatgcgcaatagtagtaggtaatc
cagctcgatttattaaatgggtagaggataatgaataaaattgatttttagatctttttgcaattaaccagcgacagcacaaaga
attagtttctgcgtttagtagggtgctagattctggttggtatatcatgggcgaagaacttgagcagttcgagaaagagttcgc
agaatactgtggagttaagtattgcattggtgtagcaaatggccttgatgcgttgatactagtattgagggcatggaaagaact
cggctatctagaggacggtgacgaggtattagtaccggcaaatacatatattgcttctattcttgctataacagagaacaaact
tgttcctgttcttgttgaaccagatatagaaacttataatattaatcctgctttaattgaaaattacattacggaaaaaaccaaagc
aatattaccggttcacttatatggtctattgtgcaatatgccagaaattagtgcaatcgcaagaaaatataatctgttgattcttga
agattgtgcacaagcacatggtgcaatacgtgatggtcgcaaagctggagcttgggggggatgctgcaggatttagtttttatc
caggaaaaaaccttggagctttgggggatgcgggagctgttactacaaataatgcagaattatcctcaactataaaagctttg
cgaaattatgggtcacataagaaatatgaaaatatttatcagggattgaatagtcgattggatgaactgcaagcagccttattg
cgtgtaaaaatccatacattaccggaagatactgcgattcggcaaaggattgctgaaaaatatattcgtgaaataaaaaaccc
tgcgattacgttaccagtgtacgaaggccaaggtgcgcacgtttggcatttatttgtagtaagagtcgctaatcgtgaaaaatt
ccagtcatacttattagagaagggtatccaaaccttaattcactatccattaccaccccataagcagcaagcatatcaaaatat
gtctagtcttagccttccaattactgagcaaattcatgatgaagtcatttctttacctataagtccggtaatgagtgaagatgatgt
caattatgtaatcaaagtggccaatgattacaagtaatgaaaaaatttcttcaggtaactatattatccgctatctatacattcatt
aaaatgattgcgggttttatcatcggtaaggtagtagcaatttatacagggccatcaggggtagcaatgcttggccaagtgca
aagtttaatcacaatagttgcaggtactacctctgcacctgtaagcactggtcttgttcgatatactgcggaaaattggcaaga
aggacaggaagcatgcgcgccatggtggcgcgcatgcttaagggttactctgtttttattcttgcttattattcccgttgttatttt
attgtcgaaaaatattagcgagttactttttagcgatggacaatacacatggttaatcattttcgcatgttgtatattgccattctcc
attttaaatacattgatcgcttcagttttaaatggtcaacaattctataagcaatatatattggttgggatgttttctgtattcatttcta
ctatgtttatgattttgttgattgtagcttataatcttaaaggtgcattgattgccacggctataaatagtgctattgctggtcttgtat
tagttttattttgtctcaataaatcttggtttagatttaaatattggtggggtaaaacggataaagacaaaattataaaaattattcat
tatactctgatggctttggtttctgttatctccatgcctacagcattgatgtgtattagaaaaatattgattgctaaaactggttggg
aggatgcagggcaatggcaggccgtatggaagatatctgaggtctatcttggtgttgtgacaattgctttgtcaacatatttctt
accaagattgacaattataaaaacaagtttccttataaaaaaagaggtaaatagtactatattatacatagtatctattacttcatt
catggcgttgagtatctatttattccgcgatttggtaataacagttttatttactgaacagtttcgctcagctcgtgaattatttttatt
acaactcgtaggggatgtaataaaaattgctgggtttctttatgcataccctcttcaaagtcaggggcatactaaactattcatc

agttcagaagtgatttttctatgctctttatcgttaccacctatattttgttataaattatggagtacatggtgctaacataagttat
gtcattacatatagtttatattttgtgtttgcatttgtgtttactaattttattaatgtcagaagaaataattaaaaacagaggttgaaa
tttgaaaataattatacctgtcttaggatttggcagggctggtggtgaaagagttctttctaagctggcaactgaattgatgaatt
atggacatgatgtaagttttgttgttccagataatagaactaatccatattatgctaccacagcaaaaatcgtcacgagtaaatc
tagtcaaaaccgtgtaaaaatatggagaatcattaaaaattactataatctgtggcgtaaatgcatagaattaaatcctgatgct
gtagttgctagttttcatttgactgcctatcttgtcgcattattaccaatcacccgtcgtaagaaatattattatattcaggcgtatg
aagttaatttttttgataatataatatggaaattaatagcgggtttaacatattatttaccgcttaaaaaaatactaaatagtcctaat
ttgcttcctcataaacatgatgattttataggagtagttcctgcaggagtagatttaaacgttttctatccgaaaccatcaaatag
gttattaaatggtcacacatcaatagggattattggtagaaaagagaagcacaaaggaactagcgaaattatttcagtattgtg
ttcactggaaaataaagttggaattataattaatattgcgatctatcttgaagaagttgataaggagcgtttaattgctgccgggt
ttcaggttaattttttttccgattacttctgatttagaattggcatccttttatcgaagcaatgacatcatgattgcagctgggttaatt
gaagatggcgctttccattatccttgtgctgaatcaatggcttgtggttgtcttgttatttcaaattatgcgccacttactgaaacta
acagtgtacttaaattagtcaagtttgatgcttgcaaacttggtgaagcaattaatctttgtctcaatcttgacctagaagaaaaa
agcaaagaaatccaatctaatatttctgtgttgaataaatatgactggaaaattgttggtgaaactttcaatagtttattgttagat
gcaaataaatagtatacgttgatggggaaaatatgaatattgttaaaactgatattccagatctgatcgttcttgaaccaaaagt
gtttagtgatgaacgcggctttttttatggagagttataatcagattgaatttgagaagacaataggaaggcacgtaaattttgttc
aggataatcattcaaaatctagtaaaggcgtactacgtgggttacattatcaattagcaccgtatgcacaggctaaattagttc
gatgtgttgtaggtcaggtatttgatgttgctgttgatcttagaaaaaattcaccaacgttcaaaaaatggtttggaataaccctttt
ccgcagaaaataaacgacaattatggatacctgaaggatttgctcatggtttcttggtgaccagtgatgaagctgagttcattt
ataaaacaacgaactactatgctcctggtcatcagcaagcaatcatttacaatgatcctattttaaacatcgattggcctttctgc
agtagtgctctgtcattatcgcaaaaagatcaagaagcaaaattattttcagaattattggacagtgaactgttctaataaagtgt
gccaccttatccgtctgaaggataggtggttgcttatatttttttgagtatgtttgtataatgacagaaaatagtccgaaatataaa
cacgataaaagcttaataagttttatctacttatttttttatatttacacttattgtaggcttcattatcgcaaatacccagtatttggga
cgaagtagagactatgataattatatacagatcttttctggtgaagaagggggggggttcttgaattattttatcgcggattgat
gttaataacgaccagctatgaaactatcatttttataattttaacatgttcttttttttataaaggcaaggtttcttgctaactattcgcg
taatttttcaggcttgaccttattctttgtttattatgcaagcgttgcactttgggttttagattatactcaattcagaaatggtctatgt
atttccattttaatgttttccgtatactatttatttataaataaaccgacttatttttatttctcgctattatgtgcaattgcaactcattgg
tctgctttgcctttttttgcttttatatccttttgtctattcaaaaaaaaataagacaccttggttatttttgtttcagtattcttgttttgattg
cgatctcaggagaaggaaaagagatcatttcttttataagaaattttggagtgggacaaaaaaataggaaacgaagctggtgt
aaatttaataaattcattatcccttaccgctattttctggtttattattagttacatatcaagcattggaaatgaaaggagaaatttaa
gacttttcttttgttatggtgtcatgcaatacgtgacttttagccttttctctctacctgttatggctttccgtattttggaaatgtattttt
tccttatgttaaccatcggggtgtttattaagcaaaaaaagaattattattttgtttttttgcaaagtgttaattttattgtatctaacata
ctattatcatatggtttttggagtgattaatgtgtaaggctaaggtgttggctataattgttacttacaacccggaaattattcgttt
gacggaatgtattaactctttagccccacaagttgagagagtaattcttgtagataatggctcaaataatagtcatttgataaaa
aatatcattattaataaccttgaaattattttgctttcggaaaacaaaggcattgcatttgctcagaaccatggtgttaagaaggg
cctggaagcaaaagagtttgactatttatttttctcagatcaggatacttgctttcctagcgatgttattgaaaaacttaagagtac
atttacgaaaaataataaaaaaggtaaaaatgttgcttgtgcttctcctttttttaaagaccatcgttcaaattatatgcatccgtca
gtctgcctaaatatttttacgagtacaaaagttatatgtagtgaagtagacgatgatctttatccctcgcatgttattgcttctggg
atgttaatgtctcgtgaggcatggcgcgtcgtcggaccattttgtgaaaaactctttatagactgggttgatacagaatggtgtt
ggcgtgcattagctaataatatgattattgttcagacaccatcagtcatcatttctcatgaacttgggtatgggcagaaaattttt
gctggtcgatctgttacaatacataattctttcagaaatttttataaaatacgtaatgcaatatacttaatgctgcattcaaattata
gctttaagtatcgttatcatgctttttttcatgcgacaaagaatattgtatttgaaattttatattcgaaagaaaaattaaattcactg
aaggtttgttttaaagctgtacgtgatggtatgttcaataatttttaatacaaaaatagttaggctcaaggtgtttaaatggaagaa
aataatatgaagacggtcgctgtagttggcacagtgggtgttcctgcttgttatggtgggtttgaatcacttgttcagaatctaat

tgattatcaatctgatggtatacaatatcagatattttgctcttcaaaaaaatataataaaaaattaaaaaattataaaaatgcaga
attaatctatttgccgataaatgccaatggcgtctctagcataatttatgacattatgtgtttaattatttgtttattcaaaaggccag
atgttgttttaatattgggggtatctggttgtttatttctaccaatttataaactattttcaaaatcaaagatcattgtcaacatcgatg
ggcttgaatggcgtagaaataaatggggaacgtttgctaagaaatttctcaaaatatctgaggcgatatctattagaatagctg
atattatcatttcagataatcaagcaatagctgattatgtggaaaataagtacaataaaaaaagtgtagttatagcttatggggg
agatcatgctactaatcttagtacaccgatagacgatgatcaaaaaaaagatggttattatttggggctttgtaggatagagcc
tgagaataatatagaaatgattctgaatgccttcattaatacagataaaaaaattaaatttatgggtaattgggataatagcgagt
atgggcgccagctaaaaaaatattattcaaactatccaaatatcacactactagaacctaactataatatcgaagagctttataa
actaagaaaaaattgccttgcatacattcatggacactcggctggtggaacaaacccttctttagttgaagcgatgcattttaat
attcctatttttgctttcgattgtgactttaatcgttacacaactaataatttagctcattactttaatgattctgaacaactgacctcat
tagcagacagtttgtcttttggaaatcttaaatgtcgagtattagatttaaaaaattatgctgaagatatgtataactggaggcat
atagctgctatgtatgaatctatttattaaacgcattaacaataatataattgaccttatatagctatcagagtaaaaatgtaacgc
tgattttttgttaaatgatgcgcattatatattcaatgattaaaacactcttgatcaaataaaactatctaattaataatgtaaaattt
cattatgctatttaagatgtgaaaaataacttaaataagggatgggatatatttatattatcccggttatagacagggaaagatc
atcagaagtacgctttttgtactaaataattcgcattttatgtttaaaaattgagatattcctcattacctgaagctgtttttttattactt
atacatgatcaaatactccttacataattaaggagagcaaaatgggacttaaaaaattgatggaacatatttctattattcccgat
tacagacaagcctggaaagtggaacataaattatcggatattctactgttgactatttgtgccgttatttctggtgcagaaggat
gggaagatatagaggattttggggaaacacatctcgatttttgaagcaatatggtgattttgaaaatggtattcctgttcacgat
accattgccagagttatatcctgtatcagtcctgcaaaatttcacgagtgctttattaactggatgcgtgactgccattcatcaaa
tgataaagacgtcattgcaattgatggaaaaacgctccggcattcttatgacaagagtcgccgcaagggagcgattcatgtc
attagtgcgttctcaacaatgcacagtctggtcatcggacagattaagacagatgagaaatctaatgagatcacagctatccc
tgaacttcttaacatgttggatattaaaggaaaaattatcacaactgatgcgatgggttgccagaaagatattgcagagaagat
acaaaaacagggaggtgattacttattcgctgtaaaaggaaatcagcgtcggcttaataaagcttttgaagaaaaattcccac
tgaaagaattaaataatccagagcatgacagttatgcaattagtgaaaagagtcacagcagagaagaaatccgtcttcatatt
gtttgcgatgaccctgatgaacttattgatttcacgtttgaatggaaaggactgaaaaaattatgcgtggcagtctcctttcgct
caataatagcagagcaaaagaaagagcccgaaatgatggtcagatattatatcagttctgctgatttaaccgctgagaagttt
gcgacagcgattcgaaatcactggcatgtggagaataagctgcactggcgtctggacgtagtaatgaatgaagacgattgt
aaaataagaagaggaaatgcagcagaattattttcagggatacggcacattgctattaatattttgacgaatgataaggtattc
aaggcaggattaagacgtaagatgcgaaaagcagccatggacagaaactatctcgcgtcagtccttgcggggagcgggc
tttactagtccttccttactcagtgacagaatattgaatgaatagcattaaaattattaaataatatggtttaattcgacatgaacttt
aagaatataattagaataatactaccttttttgtattttcttgagtattttactttactcaggaagggcagatagtgttttgttatttacta
tcgaatacacacttgcaagtatggcaatctttctgataaaaagaaagtttataagcaatatcttgtgggggatttatctctcaattt
taggtgtacaggcagcatcgctatttagttcaggacaatatttgatgcctcttgccttgtcgaatgctgcagaagttgaatcgct
aggcattggcgaggtaatcaaggtttttctatttttattttatttgttatagtttcattcgttacgatgccttctggtgctctggtcgg
ccgtattaaatattataaatttagtttttctgtatagtattattgcttgtgtgttctttcataagcacgggaccggtatatgctttttatg
aaacagcaaaattgtactatttacagcaaacctttaagcctagtgagaagatcggtgatgccgggaaatctttttaaaaacaa
gtgtttttattacccctctacagaataacaacaacttcaaagggcgtaatgttattgtaatctttgctgagggattttcatcggaga
ttattggtaattcaaaaatggcgcattttccaattacgcctaatttagataagttatctactgaagcactaagttttaaaaattattat
aaccatacagcagcaacctttcgaggtttaagaggtcagttaacttctggctatcaatttcgtgatgggttaactgatgctggta
ctggtattgcacaattatctaacgtagaaattaataatatttatcttcatcgtcaaacatctttgccggatatattgaaagagtatg
gctataaaagttattttatagcgtctacagaaaaaatagcccactcaacactatgttgaaaacgcttaattttgataaagttcttg
gtatgggagattttattggttatcaacgcgacagaatgactgataagcaaacatttaatgcactgcaatatttttttacgttctcaa
gaaaataaaaacgaacgctttttttattggtgtgtacccatctggtactcatcatggtcaagatagtccaaatgaaaaatattttga
tggtagcaatcctctctacaataaattctataactatgattttcagttggggaagtttgttgatttttttcggcgcagttcattttatg

gcaatacgttgttgataattacatcggatcattctacatttccttcgacagaatataagaaatcatttaacagcgattctcgttattt
tgtggacaaaataccatttctgatcattggaaaaaatattacgcctgaagttttagatgcaggcggtgaaaattctttaagtttcg
cgccaacaatattgcacatgttaggaattcagtattcaatgaattatttttggggtgttctcttcttgataagacgtgcacatcac
cattctcacatctttctgctattggtagtgattatttcaatataggcaaagaaaatcatgttgaactaattcaaaataatagtggta
atgaattaataaaattagccgaacgtttttataacattagtggctaaatataaaaatgtacaagttacaagcaatttcctaattag
gaaatatatgactgtaaaagaagatataaattaagctattctttatcttcaataagccgcaaccccgcggtgatcacccctgac
aggagtaaacaatgtcaaagcaacagatcggcgtcgtcggtatggcagttatgggacgcaaccttgcgctcaacatcgaa
agtcgtggttataccgtctctattttcaaccgctcccgcgaaaagacggaagaagtgattgctgaaaatccaggcaagaaac
tggctccttactatacggtgaaagagtttgttgaatctctggaaacgcctcgtcgcatcctgttaatggtgaaagcaggtgcag
gcacggatgctgctattgattccctcaagccatacctcgataaaggtgacatcatcattgatggtggtaataccttcttccagg
acactattcgtcgtaaccgtgagctttctgcagaaggctttaacttcattggtaccggtgtttccggcggtgaagaaggtgcg
ctgaaaggtccttccattatgcctggcggacagaaagaagcctatgaactggttgcgccgatcctgactaaaatcgccgca
gtggctgaagacggtgagccatgcgttacctatattggtgccgatggtgcaggtcactatgtgaagatggttcacaacggta
ttgaatacggcgatatgcagctgattgctgaagcctattctctgcttaaaggtggcctgaatctcaccaacgaagaattggcg
cagacctttaccgagtggaataacggtgaactgagcagctaccttatcgacatcaccaaagatatcttcaccaaaaaagatg
aagacggtaactatctggttgatgtgatcctggatgaagcggctaacaaaggtaccggtaaatggactagccagagcgcg
ctggatctcggcgaaccgctgtcgctgattaccgagtctgtgtttgcacgttatatctcttctctgaaagatcagcgtgttgccg
catctaaagttctctctggcccgcaagcacagccagcaggcgacaaggctgagttcatcgaaaaagttcgccgtgcgctgt
atctgggcaaaattgtttcttatgctcagggcttctctcagctacgcgccgcgtctgaagagtaccactgggatctgaactac
ggcgaaatcgcgaagattttccgtgctggctgcatcatccgtgcgcagttcctgcagaaaatcaccgatgcatacgccgaa
aatccgcagatcgctaacctgctgctggctccgtacttcaagcaaattgccgatgactaccagcaggcgctgcgcgatgtc
gtcgcttatgcagtacagaacggtatcccggttccgaccttcgccgctgcggttgcctattatgacagctaccgtgccgctgt
tctgcctgcgaacctgatccaggcacagcgtgactatttcggagcacatacttataagcgtattgataaagaaggtgtgttcc
ataccgaatggctggattaatctgatttaatcaacaataaaattgaggcccggcgtatattgtgccgggtttttttgccaattat
cttatagactaaatttcactgcttaatattaacttaataaatatcagctattcttgtaaagaaattctgaattgtttttcgttgcgttga
ccatctgacaacgtagcgttaaaacttttaactcttatcaggatgctaaaaacatcatgattcacagttaagttaattctgagagc
atgaaatgaaaatcaccatttccggtactggctatgtaggcttgtcaaacgggcttctaatcgcacaaaatcatgaggttgtgg
cattagatattttaccgtcacgcgttgctatgctgaatgatcggatatctcctattgttgataaggaaattcagcagtttttgcaat
cagataaaatacactttaatgccacattagataaaaatgaagcctaccgggatgctgattacgtcatcatcgccactccaacc
gactatgatcctaaaactaattatttcaatacatccagtgtagaatcagtaattaaagacgtagttgagataaatccttatgtggtt
atggtcatcaaatcagaggtcgacggtatcgataagcttgatatcgaattcctgcagcccgggggatccactagttctagag
cggccgccaccgcggtggagctccaattcgccctatagtgagtcgtattacgcgcgctcactggccgtcgttttacaacgtc
gtgactgggaaaaccctggcgttacccaacttaatcgccttgcagcacatccccctttcgccagctggcgtaatagcgaag
aggcccgcaccgatcgcccttcccaacagttgcgcagcctgaatggcgaatggaaattgtaagcgttaatattttgttaaaat
tcgcgttaaatttttgttaaatcagctcattttttaaccaataggccgtactgcgatgagtggcagggcggggcgtaattttttta
aggcagttattggtgcccttaaacgcctggttgctacgcctgaataagtgataataagcggatgaatggcagaaattcgaaa
gcaaattcgacccggtcgtcggttcagggcagggtcgttaaatagccgcttatgtctattgctggtttaccggtttattgactac
cggaagcagtgtgaccgtgtgcttctcaaatgcctgaggccagtttgctcaggctctccccgtggaggtaataattgacgat
atgatcatttattctgcctcccagagcctgataaaaacggtgaatccgttagcgaggtgccgccggcttccattcaggtcgag
gtggcccggctccatgcaccgcgacgcaacgcggggaggcagacaaggtatagggcggcgaggcggctacagccga
tagtctggaacagcgcacttacgggttgctgcgcaacccaagtgctaccggcgcggcagcgtgacccgtgtcggcggct
ccaacggctcgccatcgtccagaaaacacggctcatcgggcatcggcaggcgctgctgcccgcgccgttcccattcctcc
gtttcggtcaaggctggcaggtctggttccatgcccggaatgccgggctggctgggcggctcctcgccggggccggtcg
gtagttgctgctcgcccggatacagggtcgggatgcggcgcaggtcgccatgccccaacagcgattcgtcctggtcgtcg

tgatcaaccaccacggcggcactgaacaccgacaggcgcaactggtcgcggggctggccccacgccacgcggtcattg
accacgtaggccgacacggtgccggggccgttgagcttcacgacggagatccagcgctcggccaccaagtccttgactg
cgtattggaccgtccgcaaagaacgtccgatgagcttggaaagtgtcttctggctgaccaccacggcgttctggtggcccat
ctgcgccacgaggtgatgcagcagcattgccgccgtgggtttcctcgcaataagcccggcccacgcctcatgcgctttgc
gttccgtttgcacccagtgaccgggcttgttcttggcttgaatgccgatttctctggactgcgtggccatgcttatctccatgcg
gtaggggtgccgcacggttgcggcaccatgcgcaatcagctgcaacttttcggcagcgcgacaacaattatgcgttgcgta
aaagtggcagtcaattacagattttctttaacctacgcaatgagctattgcgggggggtgccgcaatgagctgttgcgtacccc
cctttttttaagttgttgatttttaagtctttcgcatttcgccctatatctagttctttggtgcccaaagaagggcacccctgcggggt
tcccccacgccttcggcgcggctccccctccggcaaaaagtggcccctccggggcttgttgatcgactgcgcggccttcg
gccttgcccaaggtggcgctgcccccttggaacccccgcactcgccgccgtgaggctcgggggggcaggcgggcgggc
ttcgcccttcgactgcccccactcgcataggcttgggtcgttccaggcgcgtcaaggccaagccgctgcgcggtcgctgc
gcgagccttgacccgccttccacttggtgtccaaccggcaagcgaagcgcgcaggccgcaggccggaggcttttcccca
gagaaaattaaaaaaattgatggggcaaggccgcaggccgcgcagttggagccggtgggtatgtggtcgaaggctgggt
agccggtgggcaatccctgtggtcaagctcgtgggcaggcgcagcctgtccatcagcttgtccagcagggttgtccacgg
gccgagcgaagcgagccagccggtggccgctcgcggccatcgtccacatatccacgggctggcaagggagcgcagc
gaccgcgcagggcgaagcccggagagcaagcccgtagggcgccgcagccgccgtaggcggtcacgactttgcgaag
caaagtctagtgagtatactcaagcattgagtggcccgccggaggcaccgccttgcgctgcccccgtcgagccggttgga
caccaaaagggaggggcaggcatggcggcatacgcgatcatgcgatgcaagaagctggcgaaaatgggcaacgtggc
ggccagtctcaagcacgcctaccgcgagcgcgagacgcccaacgctgacgccagcaggacgccagagaacgagcac
tgggcggccagcagcaccgatgaagcgatgggccgactgcgcgagttgctgccagagaagcggcgcaaggacgctgt
gttggcggtcgagtacgtcatgacggccagcccggaatggtggaagtcggccagccaagaacagcaggcggcgttcttc
gagaaggcgcacaagtggctggcggacaagtacggggcggatcgcatcgtgacggccagcatccaccgtgacgaaac
cagcccgcacatgaccgcgttcgtggtgccgctgacgcaggacggcaggctgtcggccaaggagttcatcggcaacaa
agcgcagatgacccgcgaccagaccacgtttgcggccgctgtggccgatctagggctgcaacggggcatcgagggcag
caaggcacgtcacacgcgcattcaggcgttctacgaggccctggagcggccaccagtgggccacgtcaccatcagccc
gcaagcggtcgagccacgcgcctatgcaccgcagggattggccgaaaagctgggaatctcaaagcgcgttgagacgcc
ggaagccgtggccgaccggctgacaaaagcggttcggcaggggtatgagcctgccctacaggccgccgcaggagcgc
gtgagatgcgcaagaaggccgatcaagcccaagagacggcccgag

pBBR-O6:

accttcgggagcgcctgaagcccgttctggacgccctggggccgttgaatcgggatatgcaggccaaggccgccgcgat
catcaaggccgtgggcgaaaagctgctgacggaacagcgggaagtccagcgccagaaacaggcccagcgccagcag
gaacgcgggcgcgcacatttccccgaaaagtgccacctgggatgaatgtcagctactgggctatctggacaagggaaaa
cgcaagcgcaaagagaaagcaggtagcttgcagtgggcttacatggcgatagctagactgggcggttttatggacagcaa
gcgaaccggaattgccagctggggcgccctctggtaaggttgggaagccctgcaaagtaaactggatggctttcttgccg
ccaaggatctgatggcgcaggggatcaagatctgatcaagagacaggatgaggatcgtttcgcatgattgaacaagatgg
attgcacgcaggttctccggccgcttgggtggagaggctattcggctatgactgggcacaacagacaatcggctgctctga
tgccgccgtgttccggctgtcagcgcaggggcgcccggttctttttgtcaagaccgacctgtccggtgccctgaatgaactg
caggacgaggcagcgcggctatcgtggctggccacgacgggcgttccttgcgcagctgtgctcgacgttgtcactgaag
cgggaagggactggctgctattgggcgaagtgccggggcaggatctcctgtcatctcaccttgctcctgccgagaaagtat
ccatcatggctgatgcaatgcggcggctgcatacgcttgatccggctacctgcccattcgaccaccaagcgaaacatcgca
tcgagcgagcacgtactcggatggaagccggtcttgtcgatcaggatgatctggacgaagagcatcaggggctcgcgcc
agccgaactgttcgccaggctcaaggcgcgcatgcccgacggcgaggatctcgtcgtgacccatggcgatgcctgcttg
ccgaatatcatggtggaaaatggccgcttttctggattcatcgactgtggccggctgggtgtggcggaccgctatcaggaca
tagcgttggctacccgtgatattgctgaagagcttggcggcgaatgggctgaccgcttcctcgtgctttacggtatcgccgct

cccgattcgcagcgcatcgccttctatcgccttcttgacgagttcttctgagcgggactctggggttcgaaatgaccgaccaa
gcgacgcccaacctgccatcacgagatttcgattccaccgccgccttctatgaaaggttgggcttcggaatcgttttccggg
acgccggctggatgatcctccagcgcggggatctcatgctggagttcttcgcccaccccatgggcaaatattatacgcaa
ggcgacaaggtgctgatgccgctggcgattcaggttcatcatgccgtttgtgatggcttccatgtcggcagaatgcttaatga
attacaacagtttttatgcatgcgcccaatacgcaaaccgcctctccccgcgcgttggccgattcattaatgcagctggcacg
acaggtttcccgactggaaagcgggcagtgagcgcaacgcaattaatgtgagttagctcactcattaggcaccccaggctt
tacactttatgcttccggctcgtatgttgtgtggaattgtgagcggataacaatttcacacaggaaacagctatgaataggata
caacgtgtgcataattatttaaggcttaaagataaaataaaaaacgtatttttagggttatatatattgcagttatttaattatatcgc
gccattggtaattatccctatcctgataaaatatattgggttgggggaatatggggaattagtctatattacatctatttatcaaat
agtggctttgtttattgattttggctttacttacacaggacctgtggttgctgcgagacataaatgtgagatccaaaatttacagc
gctattactcaatagttgtttttttaaaatcattgctttttataattgcattaacatgtgtatttttattgtgcagattaaatatagtccact
tgtcatttttggggtttttgtcaatttttctatgctctattggtaatatagtatcgcccaattggtttttgcaggggattggtgattttaa
aaaactttcatactctcaagtaatagtgagaataacattgtttatcatgcttcttgtttatgtctgtagtggcggagataatgtttta
tcttaagtttttacaaaatgcaacattactcatatgctgtatatacttatggccaaatattcatattagccatgttgttcatcttaaac
ctaatgaatgcattgtggaatttaagaaggcaggaaatgtttttattggcgtaataggtacgattggttacaatggtctaattcct
gtgttaattggaaacctttgcggtaatacgagtcttggtgtttttcaatcgttcaaaaaatgacaacagcatgtcaaagcctaat
taatccaatatcacagtatatgttatcacaagtttcagaaattaaacctcaagatgaactgttttattatagaattaaaaaaagtttt
tttgtgcatttaacaattagcataattgcatgtttatgttatatggggttagggcaatatgtggcgacttttataggtaaagttgac
gtttcatttgttattattttatatgcgtcagtaattacaatttttcatctttaaataatgtccttggtatacagtttcttataccgacagat
aatgtaaaaatactgcgaagtataaatgttatagcgggaattattgttgttagtttgtcctggctgttaatatcacgctttgacattc
tgggggggggttttattaaatctaattggtgagtttcttgtattcagtatgctagcttttattgcccatcgtaagtggggagcgaga
gtataatgaaagtgaaggcggttcctgctattacattctatttaagtttaatgctgacaattttagtgttactgtttggtaatgaacc
aaataaatcacaatatattcttgttatagcaacgataacagtttttatatcgcgtatatcactaataaaataacttctccggccag
ccttctcgttatatcatcttttgtgtttttaggttgtcgacctttattatctttgtttgcaaactatgattacaggattgccgattggttta
ttgaaggatatatggatgacgatgtgattatggctaactatgctataacattaatgtattatggttatacattgggactaattctat
gcagaaatactgaaaaaatttatccgcatggaccatatccagagaaacaattgctaaaaataaagtttcttttgactttattttttc
tgggttcgataggtatggttgtgaaagggatattctttttaactttatagaatctaatagttatgttgatatatatcaatcaaatata
acaacgccaataggttatgattttctgtcttatttgttttattgttctttttccttatatgtgcgtttcatatacagttcagaacaaataa
aaaatttctttttattgcgatatgcattgctgcatttagcaccttgaagggtagtcgtagtgaagctataacgtttcttttaacggtt
acatgtatatattttaatgaagtaaagacaagaaacttacgtttgctgattacaatgatttttgtttttagcgtcatttttgtgattagt
gaatttatctcaatgtggcgcactggagggagttttattcaattaatgcagggtaataatcctgttataaactttgtatacggcat
gggagtatcatatctttccatttatcaatcagtaaaactacaactattgtcagggggggtataatgttacctatctattcagccagtt
aataataacttgctcgtcaatatttaatgttaaattgagcttgccggaaataagctatagccatttggcctcatacacagcaaac
ccagaactatataatcttgggttcggacttgggggaagttatttagcagaatcgtttttagcatttggtcttattgggtgtttcata
atacccttttttacttttacttaatttaaatgtattggaaaaatacacaaaaaacaaaccaatcatatattttgtttattatagtgtgttg
ccacctatattattcacaccaagagagactttgttctatttcttcccctatcttgtcaaaagtatatttgttgctttttttagttacattat
acatccagtataaaaaggattgacgaaaatgtcagaaaaaaatgtcagcataataatcccaagttataacagggctcatattc
ttaaggaggtcataccaagttattttcaggatgagactttagaggtaatagttatcaatgatggatcaacagataatacaaatag
tgtattagctgaactgaaggaaaaatattctcaattagttattttagaaaatgatacgaacaaaaaacagatgtattctaaaaac
cgagggattgaaatagccaaagggaaatatatttttttggtgatgatgactcttacctcttacccggtgttatatctcggttattg
gctacaaaatatgagacaggcgctgatgtaatcggcgcaagaatactctatatgaataataacgagaaaacaattgaagatt
gcataaatcgacataaaacagaggggcgttttgttagtgatctaaatagattggattttagttatacatgtgatttggaccatcc
gattgaatgtttttatgcacagtctattattctagctgaaagggaactaatatcgaaatatcgatttgatatatcttatacgggaaa
ctgctatcgtgaggaaactgatttcatgttatctctttttataaaaaataaaaaatttatatatgattcaaaggctctgttaataaattt

acctccacgaaaagcgacgggaggggcaagaacagctaatcgattaaaatatcattatgaaagttgcataaataattataga
tttttaaaaaaatataatgataatttgaatcttctttcaggacaaaagcatgctatattttaccgacagtgtcaattcgttctgctaa
aaattaagtcgtttatcgggaagtttttaaaatgattgtatatattgccgcgtataatggttcaggagggcaaggtggggtgga
aagggttgttgcccaacaatgtaacattcttaaaaatttgggggttaaagtcattatacttgataaagcatacttcaaaatttctaa
caaaattcgtaacaaaaaaatacaagtagcactttatccaatattagtttctctttatttaaccttacaaaaattacgtggcgtgac
gtttaaagttattgcgcatggctattgttctcctttttataggaatgacatcttaatagctcatggcaatatgaaatgttattttcaaa
cagtcatgaaaaaaaaaacctaatcgattgtccggcagtggtcttttatctttctatgagcgtttggctggatcattctcaaaaaat
atctgggctgtttcaaataaggttaaaagtgaatggaatgagctttacaatattaattcacataaaattaaagttgttcgaaatttt
ataaatcttgaacaatttgattgcactgatgttaatgaagcacaatatgtgacatttgtcgggcgattggaaaaaggaaaagga
atagatgatctgtattacatatgtaaaaatctgccagatacttccttccatttagtttcaagtattcccgccccacaaaattttgctt
cgctaaataatgttctgaccagcattgctgtcccctatgcgaaaatgccagaaatatttaagaaatccagagtacttattttacc
gtcctattatgaaggatatgagctggttactattgaagcgctatgctgtggttgccctgtgataggctataacgttggtgcaatt
agagagttgtatgctgaaagttttcctggcgtgtttattgccaataataaagaagatttagcacaagtagcctacgaactaatta
gtcttgataatgaaaaatattatcatttgagacaaactatttatagcaagcgtgagctttttttctgaagagagatatgcggaaattt
taacggcggcatttaatgaaaaaaaaataagaaactatgtctcatttcaattaactcatataatgaacttaccggaggaggagta
tatttacgtacgcttgttagttttctacaaaaacagaatgttaatttaacacttattgataaaaaaatcttcaggtaaactattcgaag
acaatacttttcaacatatatcatttattaaaggtaaacgtcaggatattatatccaggctttttttataccatcattttatgtcccctta
tattttctcaataattaaaattttacggaagcaagatattctagcttttcacaactctcgacttggattgttatgtctgctttttagaat
actcatgccccataaaaagatcatattgtttacggataacttcgaatatgacttaataagacaaaaagataaaaacataactact
tttgttgaaaaaataattgtttatctcaatgaatttatcgggcttaagaattcacatttagttagctatattacccggcaagataaaa
atgcaatggataaattttatggggttaaaaaaagcagaaatttaattctccctgtgatatttagtagagaaaagccaactgatgt
attatcagctcactttattaatgagtataatcgattgaataatgataataggaaaaaagtagtatttactgcatctttgattttttcc
aaatatagatgctgccaactatgttttaaatgcagcaaagtcgaataatgattattgctatattttggcaggtaggaaaagtact
actttgaatcttcctgatttggataatttattttttttcgataatctatctaatagtgaaatgtcatatttattatctgcttgtgatatttttt
attctcctatagttttaggaagtggaatgaaaacaaaaattgcagaagcgctatcatatggattatatatttatgcgacagagca
ttccttaatcggctatgatgaaattataaacaacaaggagtgtgttaaaaaaatctcacatttggatgaggaatttcctaaagatt
tcaaggtgaaaagtatcaataaacagctaataatgtcttatcagcaaaaatattattcacattatcggtttaatggccatgaactt
gacatgataaatattgacgattagttagtggagatataatatgaacatattagtaactggtggtgctggatatatcggatctcat
acggctattgaattactaaatgcaggtcatgagattatcgttctggacaatttcagtaatgcttcatacaagtgtatcgaaaaaat
aaaagaaattactcgacgtgattttataacaattacaggagatgctgggtgtaggaagacactctccgctattttcgagaaac
acgccatagatatagttattcattttgctggctttaaatctgtttcagagtctaaaagtgaacccttaaagtattaccaaaataatg
ttggagtgaccattactttattacaggtaatggaagagtacaaaattaaaaaatttatctttagttcatctgcgacagtctatggtg
aaccagagataattccaatttcagaaacagctaaaattggaggaactacgaatccatatggcacatcgaaatattttgttgaaa
aaattctagaggatgttagttccacgggaaaactggatataatttgcttgagatattttaatcctgtcggtgctcattctagtggta
aaataggtgaagctccatctggtatccctaataatcttgtcccttatttattggatgttgcaagtggtaaacgtgataaattatttat
ttatggcaatgattatcctactaatgatggaacaggtgtaagagattttattcatgttgttgacttagcgaaaggtcatttggctg
caatgaattatttaagtatcaattcgggatataatatctttaatcttggtacaggaaaaggttattcagtgcttgaattaatcactac
atttgaaaaattaacaaacattaaggtcaataaatcttttatagagagagaagggcaggggatgttgcgtcttgttgggctgatgc
agataaagctaattctttattggactggaaagccgaacaaactctagaacagatgttattggactcgtggcgttggaaaaaaa
attatccagacggattctgaataaaaaaggtttcagttttatgaatcaatcagagcagagaaaaaaaaatactggttcttacacct
cgctttccctaccctgtcattggaggggatagattaagagtctatatgttatgtaaagaactttccaaaaaaatatgatcttattctt
ctgagcttatgtgatcaaccactagaacttgaaataaatataaatgactcggtcttcaaagaaattcatcgtgtctatctaccaa
aatataaatcatattataatgtatttaaagctttggttacgcaaaaaccgttgcaaattgcttattatcaatcggacacgtttaaga
ataaatacaataaattaattaaacaatgcgatgcagtattttgtcatctaataagagttgctgattatgttaaggatacagacaag

ttcaaaattcttgatatgacagatgcaatatctttgaattacagtcgcgttaaaaaattagcaagtaaaaaaagtttgcgtgcaat
tatttattctctggaacaaaaaagattagaatcatatgagcgttctgtggcgaatctttttgatttgaccacttttatttcatccgtag
accgtgactatctctaccctaatccgggcagtaatatccatatagtcaataatggggttgatacatcagccttgagatatataaa
aagagaaataaaaatcgataagccggtggaacttatatttatcggaaatatgtattctttacaaaatatggatgctgcaaaaaat
tttgctaagaatattttaccttgcttgcatgatgagtttaatattatttttaaagtgattggtaagatctcagaaactaataaaaatat
attaaattcatttaaaaatacaattgctttaggtactgttgatgatatcaattcttccgcttctacagggcatataggtatatgtcct
gttcgtcttggagcaggcgtacaaaataaaattcttgaatacatggctttaggtttaccatgtattacatctagcattggttatga
aggtattaatgcaaaatctggtagcgaaatttttgttgcagatacagtagagcaatataaaaacgtactaagagaaataattta
cgattataatcgttatactgaagtggctgaaaatgcccgtagttttgtagaaaataatttttcttgggaatcaaaagttgccaattt
aatgaatatattagatgagaaattatatgaacaataataaaattattacacctatcattatggcaggtggttcaggcagtcggtt
gtggcgactatcaagaattctctatccgaaacaatttcttagcctaaacggtagtcataccatgcttcaaacaacggctaatcg
tctggatggtttggattgtaccaacccttatgtcatttgtaatgaacaacaccgctttatagttgctgaacagcttagaaaaatcg
atagattgacttcaaagaatatcatccttgagcctgttgggcgtaacactgcccctgcaattgcattagcggcgttgctgatgt
ctaagtctgataaaagtgcagatgatcttatgctcgtactggctgcagatcacgttatattagatgaagaaaaatttgtaacgc
tgttagatcggcaattccatacgctgctgatgggaaattggtaacatttggtataattccagacaaagcagaaactggttatgg
ttatatacatcgaggacaatatattaatcaggaagattcggatgcatttatagtgtcatcatttgttgaaaagccaaatcatgaga
cagccactaaatatcttgcttccggtgagtattattggaatagcggtatgtttttgtttagtgcaaatcgttatatagaggaactta
aacaatttcggcctgatattttatccgcttgtgaaaaagcaattgcttcagcgaactttgaccttgattttgtgcgtttagatgaaa
gttctttctctaagtgtcctgaagagtcaattgattacgctgtaatggaaaaaacaaaagacgcaattgttattccaatggatgc
tggctggagtgatgtcggttcatggtcttctctttgggaaattaatgataaagactcagacggcaacgtaatagttggggatat
tttctctcatgaaacaaagaattctttcatatatgccgaatcgggaattgttgctacagtgggagtggaaaatttagttgttgtcc
aaacaaaggatgccgttcttgtctcagagagaaataaagttcaggatgtaaagaaaatagtcgaacaaattaaaaattcagg
tcgtagcgagcattatgttcatcgcgaagtatatcgtccttggggtaaatatgattccattgacacaggggagcgttatcaggt
caaacgtataacagtaaatcctggtgaaggactttctttacaaatgcaccatcatagggcagaacattggatcatagtttctgg
aactgcaaaggtgactataggttctgaaactaagattcttagcgaaaatgaatctgtttacatacctcttggtgtaatacactgct
tggaaaatccagggaaaattcctcttgatttaattgaagttcgttctggatcttatttagaagaagacgatgttatccgttttcagg
accgatatggtcgcagctaaatttttgataatgtaacgttagtagaagaacgctaatatttttagttaatctgtaataagtattattt
gtttaaggtatatcatgtcgagtttaacctgctttaaagcctatgatattcgcgggaaattaggcgaagaactgaatgaagatat
cgcctggcgtattgggcgtgcctatggcgaatttctcaaaccgaaaaccattgtgttaggcggtgacgtccgcctcaccagc
gaaaccttaaaactggcgctggcgagaggtttacaggatgcgggcgtcgatgtgctggatatcggcatgtccggcaccga
agagatctatttcgccacgttccatctcggcgtggatggcggcatcgaagttaccgccagccataatccgatggattataac
ggcatgaagctggttcgcgagggggctcgcccgatcagcggagataccggactgcgcgacgtccagcgtctggctgaa
gccaacgactttcctcccgtcgatgaaaccaaacgcggtcgctatcagcaaatcaacctgcgtgacgcttacgttgatcacc
tgttcggttatatcaatgtcaaaaacctcacgccgctcaagctggtgatcaactccgggaacggcgcagcgggtccggtgg
tggacgctatcgaagcccgctttaaagccctcggcgcacctgtggaattgatcaaagtgcacaacacgccggacggcaat
ttccccaacggtattcctaacccgctgctgccggaatgtcgcgacgacacccgcaatgcggtcatcaaacacggcgcgga
tatgggcattgcctttgatggcgattttgaccgctgtttcctgtttgacgaaaaagggcagtttatcgagggctactacattgtc
ggcctgctggcagaagcgttcctcgaaaaaaatcccggcgcgaagatcatccacgatccacgtctctcctggaataccgtt
gatgtggtgaccgccgcgggcggcacaccggtgatgtcgaaaaccggacacgcctttattaaagaacgtatgcgcaagg
aagacgccatctacggtggcgaaatgagcgcccaccactatttccgtgatttcgcttactgcgacagcggcatgatcccgtg
gctgctggtcgccgaactggtgtgtctgaaaggaaaaacgctgggcgaactggtgcgcgaccggatggcagcgtttccg
gcaagcggtgagatcaacagcaaactggcgcaacccgttgaggcgattaatcgcgtcgaacagcattttagccgcgagg
cgctggcggtggatcgcaccgatggcatcagcatgacctttgccgactggcgctttaacctgcgctcctccaacaccgaac
cggtggtgcggttgaatgtggaatcgcgcggtgatgtgccgctgatggaagaaaagacaaaacttatccttgcgttattgaa

caagtaattcagtaatttcatataaataagttttaaaagacggaaaagatgagatattcagtgtggtatagcaaaggtactgcta
ttcaccatctctatgagtgagttaacatctataccacatttaagccgcacactcggcggtgaacaccccctgacaggagtaaa
caatgtcaaagcaacagattggcgtcgtcggtatggcagtgatggggcgcaaccttgcgctcaacatcgaaagccgtggtt
ataccgtctctattttcaaccgttcccgtgaaaagacggaagaagttattgccgaaaatccaggcaagaaactggttccttact
atacggtgaaagagtttgttgaatctctggaaacgcctcgtcgcatcctgttaatggtgaaagcaggtgcaggcacggatgc
tgctattgattccctcaagccatacctcgataaaggtgacatcatcattgatggtggtaacaccttcttccaggacaccattcga
ggtcgacggtatcgataagcttgatatcgaattcctgcagcccgggggatccactagttctagagcggccgccaccgcggt
ggagctccaattcgccctatagtgagtcgtattacgcgcgctcactggccgtcgttttacaacgtcgtgactgggaaaaccct
ggcgttacccaacttaatcgccttgcagcacatccccctttcgccagctggcgtaatagcgaagaggcccgcaccgatcgc
ccttcccaacagttgcgcagcctgaatggcgaatggaaattgtaagcgttaatattttgttaaaattcgcgttaaatttttgttaaa
tcagctcattttttaaccaataggccgtactgcgatgagtggcagggcggggcgtaatttttttaaggcagttattggtgccctt
aaacgcctggttgctacgcctgaataagtgataataagcggatgaatggcagaaattcgaaagcaaattcgacccggtcgt
cggttcagggcagggtcgttaaatagccgcttatgtctattgctggtttaccggtttattgactaccggaagcagtgtgaccgt
gtgcttctcaaatgcctgaggccagtttgctcaggctctccccgtggaggtaataattgacgatatgatcatttattctgcctcc
cagagcctgataaaaacggtgaatccgttagcgaggtgccgccggcttccattcaggtcgaggtggcccggctccatgca
ccgcgacgcaacgcggggaggcagacaaggtatagggcggcgaggcggctacagccgatagtctggaacagcgcac
ttacgggttgctgcgcaacccaagtgctaccggcgcggcagcgtgacccgtgtcggcggctccaacggctcgccatcgt
ccagaaaacacggctcatcgggcatcggcaggcgctgctgcccgcgccgttcccattcctccgtttcggtcaaggctggc
aggtctggttccatgcccggaatgccgggctggctgggcggctcctcgccggggccggtcggtagttgctgctcgcccg
gatacagggtcgggatgcggcgcaggtcgccatgccccaacagcgattcgtcctggtcgtcgtgatcaaccaccacggc
ggcactgaacaccgacaggcgcaactggtcgcggggctggccccacgccacgcggtcattgaccacgtaggccgaca
cggtgccggggccgttgagcttcacgacggagatccagcgctcggccaccaagtccttgactgcgtattggaccgtccgc
aaagaacgtccgatgagcttggaaagtgtcttctggctgaccaccacggcgttctggtggcccatctgcgccacgaggtga
tgcagcagcattgccgccgtgggtttcctcgcaataagcccggcccacgcctcatgcgctttgcgttccgtttgcacccagt
gaccgggcttgttcttggcttgaatgccgatttctctggactgcgtggccatgcttatctccatgcggtaggggtgccgcacg
gttgcggcaccatgcgcaatcagctgcaacttttcggcagcgcgacaacaattatgcgttgcgtaaaagtggcagtcaatta
cagattttctttaacctacgcaatgagctattgcggggggtgccgcaatgagctgttgcgtaccccccttttttaagttgttgatt
tttaagtctttcgcatttcgccctatatctagttctttggtgcccaaagaagggcacccctgcggggttcccccacgccttcgg
cgcggctccccctccggcaaaaagtggcccctccggggcttgttgatcgactgcgcggccttcggccttgcccaaggtgg
cgctgcccccttggaacccccgcactcgccgccgtgaggctcgggggcaggcgggcgggcttcgcccttcgactgcc
cccactcgcataggcttgggtcgttccaggcgcgtcaaggccaagccgctgcgcggtcgctgcgcgagccttgacccgc
cttccacttggtgtccaaccggcaagcgaagcgcgcaggccgcaggccggaggcttttccccagagaaaattaaaaaaat
tgatggggcaaggccgcaggccgcgcagttggagccggtgggtatgtggtcgaaggctgggtagccggtgggcaatcc
ctgtggtcaagctcgtgggcaggcgcagcctgtccatcagcttgtccagcagggttgtccacgggccgagcgaagcgag
ccagccggtggccgctcgcggccatcgtccacatatccacgggctggcaagggagcgcagcgaccgcgcagggcgaa
gcccggagagcaagcccgtagggcgccgcagccgccgtaggcggtcacgactttgcgaagcaaagtctagtgagtata
ctcaagcattgagtggcccgccggaggcaccgccttgcgctgcccccgtcgagccggttggacaccaaaagggagggg
caggcatggcggcatacgcgatcatgcgatgcaagaagctggcgaaaatgggcaacgtggcggccagtctcaagcacg
cctaccgcgagcgcgagacgcccaacgctgacgccagcaggacgccagagaacgagcactgggcggccagcagca
ccgatgaagcgatgggccgactgcgcgagttgctgccagagaagcggcgcaaggacgctgtgttggcggtcgagtacg
tcatgacggccagcccggaatggtggaagtcggccagccaagaacagcaggcggcgttcttcgagaaggcgcacaagt
ggctggcggacaagtacggggcggatcgcatcgtgacggccagcatccaccgtgacgaaaccagcccgcacatgacc
gcgttcgtggtgccgctgacgcaggacggcaggctgtcggccaaggagttcatcggcaacaaagcgcagatgacccgc
gaccagaccacgtttgcggccgctgtggccgatctagggctgcaacggggcatcgagggcagcaaggcacgtcacacg

cgcattcaggcgttctacgaggccctggagcggccaccagtgggccacgtcaccatcagcccgcaagcggtcgagcca
cgcgcctatgcaccgcagggattggccgaaaagctgggaatctcaaagcgcgttgagacgccggaagccgtggccgac
cggctgacaaaagcggttcggcaggggtatgagcctgccctacaggccgccgcaggagcgcgtgagatgcgcaagaa
ggccgatcaagcccaagagacggcccgag

pBBR-O25:

accttcgggagcgcctgaagcccgttctggacgccctggggccgttgaatcgggatatgcaggccaaggccgccgcgat
catcaaggccgtgggcgaaaagctgctgacggaacagcgggaagtccagcgccagaaacaggcccagcgccagcag
gaacgcgggcgcgcacatttccccgaaaagtgccacctgggatgaatgtcagctactgggctatctggacaagggaaaa
cgcaagcgcaaagagaaagcaggtagcttgcagtgggcttacatggcgatagctagactgggcggttttatggacagcaa
gcgaaccggaattgccagctggggcgccctctggtaaggttgggaagccctgcaaagtaaactggatggctttcttgccg
ccaaggatctgatggcgcaggggatcaagatctgatcaagagacaggatgaggatcgtttcgcatgattgaacaagatgg
attgcacgcaggttctccggccgcttgggtggagaggctattcggctatgactgggcacaacagacaatcggctgctctga
tgccgccgtgttccggctgtcagcgcaggggcgcccggttctttttgtcaagaccgacctgtccggtgccctgaatgaactg
caggacgaggcagcgcggctatcgtggctggccacgacgggcgttccttgcgcagctgtgctcgacgttgtcactgaag
cgggaagggactggctgctattgggcgaagtgccggggcaggatctcctgtcatctcaccttgctcctgccgagaaagtat
ccatcatggctgatgcaatgcggcggctgcatacgcttgatccggctacctgcccattcgaccaccaagcgaaacatcgca
tcgagcgagcacgtactcggatggaagccggtcttgtcgatcaggatgatctggacgaagagcatcaggggctcgcgcc
agccgaactgttcgccaggctcaaggcgcgcatgcccgacggcgaggatctcgtcgtgacccatggcgatgcctgcttg
ccgaatatcatggtggaaaatggccgcttttctggattcatcgactgtggccggctgggtgtggcggaccgctatcaggaca
tagcgttggctacccgtgatattgctgaagagcttggcggcgaatgggctgaccgcttcctcgtgctttacggtatcgccgct
cccgattcgcagcgcatcgccttctatcgccttcttgacgagttcttctgagcgggactctggggttcgaaatgaccgaccaa
gcgacgcccaacctgccatcacgagatttcgattccaccgccgccttctatgaaaggttgggcttcggaatcgttttccggg
acgccggctggatgatcctccagcgcggggatctcatgctggagttcttcgcccaccccccatgggcaaatattatacgcaa
ggcgacaaggtgctgatgccgctggcgattcaggttcatcatgccgtttgtgatggcttccatgtcggcagaatgcttaatga
attacaacagtttttatgcatgcgcccaatacgcaaaccgcctctccccgcgcgttggccgattcattaatgcagctggcacg
acaggtttcccgactggaaagcgggcagtgagcgcaacgcaattaatgtgagttagctcactcattaggcaccccaggctt
tacactttatgcttccggctcgtatgttgtgtggaattgtgagcggataacaatttcacacaggaaacagctatggtgaagata
cttgttactggtggcgcaggatttattggttctgctgtagttcgtcacattataaataatacgcaggatagtgttgttaatgtcgat
aaattaacgtacgccggaaacctggaatcacttgctgatgtttctgattctgaacgctatgtttttgaacatgcggatatttgcga
tgctgctgcaatggcgcggatttttgctcagcatcagccggatgcagtgatgcacctggctgctgaaagccatgtggatcgt
tctatcactggccctgcggcatttattgaaaccaatattgttggtacttatgtcctttttagaagccgctcgcaattactggtctgct
cttgatagcgacaagaaaaatagcttccgtttacatcatatttctactgacgaagtctatggtgatttgcctcatcctgacgaagt
aaataataaagaaaaattacccttatttattgagacaacagcttacgcaccaagcagcccttattccgcatccaaagcatcca
gcgatcatttagtccgcgcgtggaaacgtacctatggtttaccgaccattgtgactaactgttcgaataactacggtccttatca
ctttccggaaaaattgattccactagtaattcttaatgctctggaaggtaaggcattacctatttatggcaaaggggatcaaatt
cgtgattggctgtatgttgaagatcatgcgcgtgcattatataccgtcgtaaccgaaggtaaagcgggtgaaacttataacatt
ggtgggcacaacgaaaagaaaaacattgatgtagtgctcactatttgtaatttgctggatgagattgtaccgaaagagaaatc
ttatcgtgagcaaatcacttatgttgccgatcgtccgggacacgatcgccgttatgccattgatgctgagaagattggtcgcg
aattgggatggaaaccacaggaaacgtttgagagcgggattcggaagacagtggaatggtacctgtccaatacaaaatgg
gttgataatgtaaaaagtggtgcctatcaatcgtggattgaacagaactatgagggccgccagtaatgaatatcctcctttttg
gcaaaacagggcaggtaggttgggaactacagcgtgctctggcacctttgggtaatttgattgctcttgatgttcactccact
gattactgtggtgattttagtaatcctgaaggtgtagctgaaaccgtaagaagcattcggcctgatattattgtcaacgcagcc
gctcacaccgcagtagacaaagcagaatcagaaccggagtttgcacaattacttaacgcaacaagtgtcgaagcgattgc
gaaagcagcaaatgaagttggagcttgggttatacactactccaccgattatgttttccctggcagtggtgacaggccatgg

ctggaaacggatgcaacagcgccgctaaatgtttacggtgaaactaagctagctggggaaaaagcgttacaagaacattg
cgcaaagcatcttattttccgtaccagctgggtatacgctggtaaaggaaataattttgccaaaacgatgttgcgtctggcaaa
agaacgcgaagagttggctgtgataaacgatcaatttggcgcaccaacgggtgctgagcttctggccgattgtacagcaca
tgccattcgtgtcgcactgaataaaccggatgtcgcaggcttgtaccatctggtagccagcggtaccacgacctggtacgat
tatgctgcgctggtttttgaagaggcgcgcaaagcagatattcccctcgcactcaacaaactcaatgcggtaccaacgaca
gcctatcctacaccagcttgtcgtccacataactctcgacttaatacagaaaaatttcagcagaattttgcgcttgtcttgcctga
ctggcaggttggtgtgaaacgcatgctcaacgaattatttacgactacagcaatttaatagtttttgcatcttgttcgtgatgatg
gagcaagatgaattaaaaggaatgatgaaatgaaaacgcgtaagggtattattttagcgggtggttctggtactcgtctttatc
ctgtgactatggcagtcagtaaacagctattaccgatttatgataaaccgatgatctattacccgctctctacactgatgttagc
gggtattcgcgatattttgattattagtacgccacaggatactcctcgttttcaacaactgctgggtgacggtagccagtgggg
gctaaatcttcagtacaaagtgcaactgactccagatgggcttgcgcaggcatttattatcggtgaagagtttattggtggtga
tgattgtgctttgattcttggtgataatatcttttacggtcacgatctgccgaggttaatggatgccgctgttaacaaagaaagtg
gtgcaacggtatttgcctatcacgtaaatgatcctgaacgctatggtgtcgttgagtttgataaaaacggtactgcaatcagctt
ggaagaaaaaccgttacaaccaaaaagtaattatgcggtaaccgggctttatttctatggtaacgacgttgtggaaatggcg
aaaaaccttaagccttctgcccgtggtgaactggaaattaccgatattaaccgtgtctatatggaacaggggcgtttatctgtt
gccatgatgggccgtggttatgcatggttagacacggggacacatcaaagtctgattgaagcaagtaacttcattgcaacaa
ttgaagagcgacaaggtttaaaggtatcttgcccggaagaaattgcttatcgtaaaggctttattgacgctgagcaggttaatg
tattagcagaaccactaaagaaaaatgcttatggtcagtatctgctaaaaatgattaaaggttactaaaaatgaatgtaattaaa
actgaaattcctgatgtattaattttggaaccggaagtttttggtgatgagcgcggtttttttatggaaagctttaatcagaaagttt
tcgaagaggctgtagggcgtaaggttgaatttgttcaggataaccattctaaatcaattaagggtgtattacgcggattgcact
atcagctggaaccttatgctcaaggtaaattagttcgttgtgtggtcggtgaggtttttgatgtagcagttgatattcgtaaatcg
tcaggtacatttgggaaatgggttggggtgaatttgtctgctgagaacaagcgtcagttgtggatacctgaaggatttgctcat
ggatttttggtacttagtgatttagcagaagttttatataaaacgaatcaatattatgctccatcacatgaacgaaatattatctgg
aatgaccccttgcttaatattaaatggccaaggaaagcactgattactctgtctgataaggatgcaaatggggaaaaattaga
actaagtgagtattgaaatgtctctcttaaaacatagtatatggaatgttgcgggctactttataccaacattaattgcaatccca
gcgtttggattaattgcaagggaaattggtgtagaactatttggtttgtatactttatcaatgatttttatagggtatgctagtatatt
tgatgctgggttaacaagagctgtagtacgcgaaatagcattactaaaaaacagactggacgattgtaatacgataatagtaa
cttctattattgctgtggtatttttagggggtatcggaggcgggggagtgtttctgcttaaagaccatattattgaactgttaaata
tgtcaccaatatactacgtcgatgcgataaagtctctaatattattatcatctctgatacctgtattcttagtcacgcaaatactatt
agcagagcttgaggggcgggaatattttggaatcctaaatatacaaaaaagtttagggaattctttaattgcagggttgcctgc
attatttgttttaattaatcaaacactttttctgcaattattggtgtagctattgcaagagttatatgcttgtggttaagctacattatg
agcaggaaaagaataactatcgatatttcatttttttcaataactgttttaaaacggttatttagatatggcgggtgggtaactata
agtaacataatatctcctatattagcgagtatggatagatttattctatctcatatccagggagcatcaaaaatatcattctataca
gtccctaatgagctggtaactaggcttggaatagttccaggctctcttgggaaagctgtttttccaaaattaagccatgcaagt
agttttacagcgtcatatgcagaacaaaaaaaaagcgtatatattaatgactgtcattgtattgcctttggttttatttatatattacta
cgcaaagtttattttaacattgtggatgggggctgagtatgcagggatttcagtcgaaatattacggattatgcttatagggtat
atctttaactgttattcacaaatctcttttgccaatatacaggcatttggaaaagcaaaatacactgcatacatccatatgatgga
atttattccttatttgataatgttatatataatttcaaaggaatgtggggttattggtgtggcgtggttatggacaattcgagtgata
attgatttttttgatgctttttttatatgagttatcgttgtaataatcttatgaaaaaagggtagcctgatgatatatattgtagtgttaaat
tggaatggggctatagataccattaattgtgttaaaagtttaatgaatttaaattatgatgattataaaattatcgttgttgataact
gttctacggataactcatatgattctataaaagaaaatcttaatgcattatatattactggtaaaagtttcattgaggtgaagtatg
aggatagaagtaaatatcaaacatttgaaaacgataaaatcatattaatacaatctccgaaaaataatgggtacgcaagtggt
aataacattggaatagagctcgcacttaatcaggaggatatgaaatacgtctgggttctgaataatgatactgaagtggataa
agaggctttaactcatttaattagtaaatgtgattcagataaaaatatagggatttgcggttctcgtttagtctattttaccgataga

gagatgcagcaaggactgggtggggtacataacagatggttatgcactacaaaaaattatgagatgggaagattagtttcca
aaaaatatgatgatgaagtcattagcaataatatagattatataattggcgcatcaatgtttttctctagagaatgtttggaaaca
gttggattgatgaatgaagaatatttttttatactatgaagagttagatatttgcctcagagcaaaagcaaagaactttaaattagg
tatttgctcagatagtttggtttatcataagataggtgcaagtactgatgggggaaagagtatgatagctgacctttgttcaataa
aaaataggctggtcattacagaaaagttttatccacaatattattggacggtatggttgtcactctttgttgtagcatttaaccgtg
ctagaagaggtgagtttaataagatgaaaagatgtttgaatgttatgtttaacttcaaacgaaataaaggtagcaaatgccatta
gaatatgcatttaatgatggtgttattaaataaatgtatagtttgatatgttattaaagggtatttaatgaaagtggctttttttatctgc
ttatgatccactatctacatccagttggtcgggcacaccttattatatgctaaaggcattatcgaagagaaatatttccattgaaa
tattaggaccggtaaatagctatatggtatacatgttaaaagcatataaattaatattaaggtgtttcggaaaggaatatgattat
agtcgttcgaagttgctttccaagtattacggtagaatatttgagagaaaattaaaaaaaattgatggtttggattttattattgca
cctgcaggttcttcacaaattgctttttttagaaacaaatataccaataatatatctatcggatacaacatatgatcaattaaaaaac
tattatccgaatttaaataaaaaaacaattataaatgatgaggatgcaagtttaatcgaacgcaaggctattgaaaaagcaaca
gtcgtatctttcccatctaaatgggcaatggatttttgcaaggactattacagattggattctgataaattagttgaaataccatg
gggggctaatttatttgaagatattcattttgctaataaaactataattcaaaagattagttatacttgtcttttcttgggggtcgatt
gggaaagaaaaggtggtaaaacagcattgaaagcaattgaatatgtaagagagttatatgggatcgatgttagactaaaaat
ttgtggatgtactccgaatcaagagattttacctgcttgggttgaattaattgataaaataaataaaaataacgttgaagaatatc
agaaattcatcgatgtgttatctaacgctgatatacttcttttaccaaccattgctgaatgttacggaatggtattttgtgaagctg
cagcttatggattgccagttgtcgccacagatacaggtggaattagttctatagttatcaacgaaaggacggggatattaatta
aagactcgtcagactataagcactttggaaatgcaattcataaaataattagctccgtagagacttatcaaaactactcccaaa
acgcaagagttagatataataaaatattgcattgggacaattgggcaaaaaagataattgagattatgtatgagcataagaat
agaagaaccaaatagcacaaaaagaattatatttttatttttacttttccttgtttttcctgattttttgttttatacattagggattgata
attttagcatttcaacgataatctccattgtattgcttttttgtttttttaagggctaaaaacacttgcaaagataatttgctaataatag
tagtattattcatattgttgtgttttaactgtctgttaagtatgctatttaatattgaacaggttttatcatttaaaattgtactttcaatgt
atagcatcttaataatggcatacgtctcctcttgttatgcacagactttgtggttatgttctgaagaaatacttaagagatccgtct
tttatttgttcgcatttctttgccttattggcattataagtattcttttacagaaaactgagattatacatgataaaagtatgattcttttt
cctgaaccatcagcatttgcattggttttcatacctatcttttcattttgtttatactatacaagggggggggggggtactattgctct
atatattatctttgggtattgcgttaggtatccagaacttaacgatgttagtaggcattgtgattagtgtttttgtgatgaaaaaaat
aacaataaggcaaactattgttatatttttaggggcatggatttttttccatgatattaagtgatttagacatttcttactatacatcgc
ggcttgattttaaaaatactacgaacttatcagtgcttgtatatctttcaggaattgaaagagctttcttgaattttattacaagttat
ggtcttggtattggttttcaacaaatgggcgtgaatggggaggtaggagtgtatcaacaaattttagctgatcttgatgcccct
atgttaaatatatacgatggctcatttatttcttcgaagttaatatctgagtttgggtttattggtgcaataatgtgcattttctatctttt
tatttttttttcgattttatctgcgtttcaaaaaaaaataagagatatccaccgcagtatattttagcatatagtttctatatgtgtttcttc
atcccccctttttatacgtggtgctggctatataaatccctatgtgtttatgttattttcatcaatatttttatgcaaatatcatgctaaaa
ttatcttgatgaaatctaatgtcaaaatggctatataatagtagattatgttatcattaatacgtaaataacatatattctaaccagg
gcataaataatgtgcataaaaaaaattaagttaattaaacgatatggcctttatggtggtcttaggcttcttaaagattattcttaac
aaaagttttattttgttcaaatgttagggttattagattttcatgttatcttagaaaagatggaagtgttagttttggaaaagggttta
catcaggtgtagtattaggaggtgatacatttatggatgctatagtgtctattggagaaaatgttcaaattaatgattatattccac
atcgcggctattaataatgccattattggtagagatacattaatagcaagtaaagtatttattagtgatcataatcatggtattttttt
ctaaatctgatatccatagttcaccaactattattccttcatctaggtctcttgaatctgcacctgtgtatattgaagagcgtgtgtg
gattggtgaaaacgtgacaatattaccaggtgcgtgtataggtaatggtgtagttattggcgcaaatagtgttgttcgtggtga
aattcttaataatgtgatctgagttgacccccgaaaacaccagacagtagatgtatcttaaaataagaggtaggcttggacata
tatctaacttaagatacaatccctgtctggaaataagagggcaagaacagaaaatgtttcgatttgggaaagataggatatgg
cttcttatgtagtatataatatcctgcattcattcggataacttcctatggaagtgtactttgctctgtctgttttcatttgttgaaaatt
tatgttaataagaagctttagataaccacttaggaactgtatgtttgatttgtccaaaagttatattgttgtaagtgcgacggcgct

ggcctccggaggtgcattaactatattaaagcaatttataaaacatgcatcacaaaattcaaatgactatattatgtttgtatctg
cgggattagagttgccggtctgtgataacatcatttacatagaaaacacaccaaaaggatggttgaaaagaatatattgggat
tggttcggttgtcgaaagtttatctctgaacataagattaacgttaagaaagtaatttctctacaaaattccagtttgaatgttcctt
atgagcagattatttacttgcaccagccaattcctttcagtaaagttgattctttttaaatgatatcacgttcgataacgtaaaact
ttttttatataaaaatttttattcctattttatatttaaatatgtgaatgcaaatacaaccatcgtagtgcaaacgaattggatgaaaa
aaggagtgttggatcaatgtgataagattagttccgaaagggtccttgttataaaacctgatatcaaaacatttaataatactaa
atttgatgtagataaggatgtatctgttaaaacactcttatatccagcgacaccacttacctataaaaatcatttggtcattctgaa
ggcgttggttattttaaagcaaaagtattttatagatgatctgaaattccaagtgacttttgaaaagaataggtacaaaaattttga
taagtttgtgcaattaaataacctaagcaaaaacattgattatctcggtgttctttcatactcgaaattgcaaaaaaaatatatgg
cggcatcttttattgtttttcctagctatatagaatcatatgggttaccactcatcgaagctgctagtttaggaaaaaaaatcatta
gtagtgatcttccttatgcccgtgatgttttaaagaattatagcggcgtagattttgtaatttacaatgatgaagatggctgggct
agggcgttgtttaatgttttaaatggcaattcgaagctcaattttaggccttatgaaaaagatagtcgttcatcttggccacagtt
cttctctattttaaaataaggtgtattatgtttaatggtaaaatattgttaattactggtggtacggggtctttcggtaatgctgttcta
agacgttttcttgatacagatatcaaagaaatacgtatttttcccgggatgaaaaaaaacaagatgacatgaggaaaaaatat
aataatcagaagcttaagttctatataggtgatgttcgcgattattcgagtattctcaatgcttctcgaggtgttgattttatttatca
tgctgcagccctgaagcaagtgccttcctgcgaattccaccccatggaagctgtaaaaacgaatattttaggtactgaaaac
gttctagaagctgcaatagctaatcgcgttaggcgaattgtatgtctgagtacagataaagccgtatatcctattaatgcaatg
ggcatatctaaagcaatgatggaaaaagtcattgttgcaaaatcacgtaatcttgatagttcaaaaacagttatctgcggaaca
cgttatgggaatgtaatggcttcacgtggatcggtcatcccattgtttgttgatctaatcaaatctggtaaaccattgaccattac
cgatcccaatatgactcgtttcatgatgacgcttgaggatgctgttgatctggtcctttatgccttcgagcatggaaataacggt
gatattttcgttcagaaagctcctgcggcaacaattcaaacattagccattgcacttaaggaattgctaaatgcccatgaacat
ccaatcaatattattggaactcgacacggggaaaaactttacgaagcgttattgagccgagaggaaatgatagcagcggaa
gatatgggtgattattatcgtgttccaccagatctccgcgatttgaactatggtaaatatgtggaacatggtgaccatcgtatct
cggaagtggaagattataactctcataatactgagagattagatgttgagggtatgaaaaaattactgctaaaacttccttttat
ccgggcaattcgttctggtgaagattatgagttggattcataatatgaaaattttagttactggtgcttcagggtttatcggccgt
aatttggttttccgccttaaggaggctggttataacgaacttattacgatagatcgtaactcttctttggcggatttagagcaggg
acttaagcaggcagatttcatttttcaccttgcaggagtaaatcgtcctgtgaaggagagtgaatttgaagagggaaatagca
acgtaactcaacagattgttgatgttctgaaaaaaaataataaaaatactcctatcatgctgagttcttccatccaggctgaatgt
gataacgcttatggaaagagtaaagcgactgcggagaaaatcattcagcagtatggggaaacgacaaatgccaaatattat
atttatcgcttgccgaatgtattcggtaagtggtgtcgcccaaattataactcctttatagcaactttctgccatcgcattgcaaat
gatgaaactattacaattaatgatccttcagcagttgttgatctggtgtatatagatgacttttgttctgacatattaaagctattgg
aaggagcgaacgaaactggttacaggacattcggtccaatttattctgttactgttggtgaagtggcgcaattaatttaccgatt
taaagaaagtcgccaaacattaatcaccgaagatgtaggtaatggatttacacgtgcattgtactcaacatggttaagttatct
gtctcctgaacagtttgcgtatacggttccttcttatagtgatgatagaggggtattctgtgaagtattgaaaacgaaaaacgc
gggccagttttcgttctttactgcgcatccaggaattactcgggggacattatcatcattccaaaaatgagaaatttattgtcat
ccgaggaagtgcttgtttcaaatttgaaaatattgtcacgggtgagcgatatgaatttaatgtctcctcagatgattttaaaattgt
tgaaacagttccggggtggacgcatgacatcactaataatggctcggatgagctagttgttatgctttgggcaaatgaaatatt
taatcgttctgaaccagatactatagcgagagttttatcgtgaaaaaattgaaagtcatgtcggttgttgggactcgtccagaa
attattcgactctcgcgtgtccttgcaaaattagatgaatattgtgaccaccttattattcatactggccaaaactacgattatgaa
ttgaatgaagtttttttcaaagatttgggtgttcgcaaaccagattattttcttaatgccgcaggtaaaaatgcagcagagactat
tggacaagttatcattaaagttgatgatgtccttgaacaggaaaaaccagaagctatgttagttcttggcgatactaactcctgt
atttcagcaataccagcaaagcgtcgaaaaattccgatcttccatatggaagcggggaatcgttgttttgaccaacgcgtacc
ggaagaaactaacagaaaaatagttgaccacactgctgatatcaatatgacatatagcgatatcgctcgtgaatatcttctgg
ctgaaggtgtaccagccgatagaattattaaaaccggtagcccaatgtttgaagtactcactcattatatgccgcagattgatg

gttccgatgtactttctcgcctgaatttaacacctgggaatttctttgtggtaagtgcccacagagaagaaaatgttgatacccc
taaacagcttgcgaaactggcgaatatacttaataccgtggctgaaaaatatgatgtcccggtagttgtttctactcatcctcgc
actcgtaaccgcatcaatgaaaacggtattcaattccataaaaatatcttgcttcttaagccattaggatttcacgattacaacca
tctgcaaaaaaatgcacgtgctgttttatcggatagtgggactattacagaagagtcctccattatgaacttccctgcactcaat
atacgagaagcgcacgaacgcccggaaggcttcgaagaaggggcagtaatgatggtcggtcttgaatctgagcgcgtttt
acaggcattagaaattattgcaacacagcctcgtggagaagtacgcttacttcgtcaggtcagtgactatagcatgcccaatg
tttcagataaagttgtgcgtattatccattcatatactgactacgttaaacgggttgtctggaaacaatactaatgaaacttgcatt
aatcattgatgattatttgccccatagcacacgtgttggggctaaaatgtttcatgagttaggccttgaattgttgagcagaggc
catgatgtaactgtaattacgcctgacatcacattacaagcaatctattctgttagtatgattgatggtataaaggtttggcgtttc
aaaagtggacctttaaaggatgtaggtaaggctaagcgtgccataaatgaaactcttttatcttttcgtgcatggcgcgcattta
agcacctcattcaacatgatacatttgatggtatcgtttattattcccccctctatttttggggcgacttggttaaaaaaataaagc
agcgatgccagtgcccaagctacctggtcctaagggatatgtttccacagtgggtcattgatgcaggtatgttgaaagccgg
ttcgccaattgaaaaatatttcaggtattttgaaaaaaaatcatatcagcaggctgaccggataggggtaatgtctgataagaa
tcttgagatatttcgtcagaccaataaaggttatccgtgtgaagttttacgtaattgggcctcaatgactcctgtgtctgccagc
gatgattatcattcacttcgtcaaaaatacgatctaaaagataaagtcatttttttctatggcggtaatattgggcatgctcaggat
atggcgaacttaatgcgccttgcgcgtaatatgatgcgttatcatgatgctcatttcctgtttatagggcagggtgatgaagttg
acctgataaaatctcttgctgcagaatggaatttaactaatttcactcacctaccttcagtgaaccaggaagagtttaaattaatt
ttatctgaagttgatgtcggcctgttctccctttcatctcgccattcttcacataatttccctgggaaattactagggtatatggttc
aatcaatcccgatccttgggagtgtgaatggcggcaatgatttaatggatgtaattaataagcacagggccggtttcattcatg
ttaatggtgaagatgataaactgtttaaatctgcacaattgcttcttagtgattcagctttaagaaagcagttaggtcagaacgct
aatgtgttgttaaagtctcaattttcggttgaatcggcggcacatattatcgaagtccgactggaggcaggagaatgcgtttag
ttgatgacaatattctggatgaactttttcgcactgcagcaaattctgaacgtttgcgcgctcattatttattgcacgcatctcatc
aggagaaggttcaacgtttacttattgcatttgtccgcgacagctatgttgaaccccattggcatgagttaccgcatcagtggg
aaatgtttgtcgtcatgcaagggcaattagaagtttgcttgtatgagcaaaatggtgagatccaaaaacagtttgtggttggag
acggtacgggaataagcgtcgtggaattttccccaggagatatacatagtgtcaaatgcttgtcaccaaaagcccttatgttg
gagataaaggaggggccatttgacccactgaaagctaaggctttttctaagtggttataaagcgatataccaccgtttattctt
ctatcttattctatacatgctgggtgaggtcgacggtatcgataagcttgatatcgaattcctgcagcccgggggatccactag
ttctagagcggccgccaccgcggtggagctccaattcgccctatagtgagtcgtattacgcgcgctcactggccgtcgtttt
acaacgtcgtgactgggaaaaccctggcgttacccaacttaatcgccttgcagcacatcccccttcgccagctggcgtaat
agcgaagaggcccgcaccgatcgcccttcccaacagttgcgcagcctgaatggcgaatggaaattgtaagcgttaatatttt
gttaaaattcgcgttaaatttttgttaaatcagctcattttttaaccaataggccgtactgcgatgagtggcagggcggggcgta
atttttttaaggcagttattggtgcccttaaacgcctggttgctacgcctgaataagtgataataagcggatgaatggcagaaat
tcgaaagcaaattcgacccggtcgtcggttcagggcagggtcgttaaatagccgcttatgtctattgctggtttaccggtttatt
gactaccggaagcagtgtgaccgtgtgcttctcaaatgcctgaggccagtttgctcaggctctccccgtggaggtaataatt
gacgatatgatcatttattctgcctcccagagcctgataaaaacggtgaatccgttagcgaggtgccgccggcttccattcag
gtcgaggtggcccggctccatgcaccgcgacgcaacgcggggaggcagacaaggtatagggcggcgaggcggctac
agccgatagtctggaacagcgcacttacgggttgctgcgcaacccaagtgctaccggcgcggcagcgtgacccgtgtcg
gcggctccaacggctcgccatcgtccagaaaacacggctcatcgggcatcggcaggcgctgctgcccgcgccgttccca
ttcctccgtttcggtcaaggctggcaggtctggttccatgcccggaatgccgggctggctgggcggctcctcgccggggc
cggtcggtagttgctgctcgcccggatacagggtcgggatgcggcgcaggtcgccatgccccaacagcgattcgtcctg
gtcgtcgtgatcaaccaccacggcggcactgaacaccgacaggcgcaactggtcgcggggctggccccacgccacgc
ggtcattgaccacgtaggccgacacggtgccggggccgttgagcttcacgacggagatccagcgctcggccaccaagtc
cttgactgcgtattggaccgtccgcaaagaacgtccgatgagcttggaaagtgtcttctggctgaccaccacggcgttctgg
tggcccatctgcgccacgaggtgatgcagcagcattgccgccgtgggtttcctcgcaataagcccggcccacgcctcatg

cgctttgcgttccgtttgcacccagtgaccgggcttgttcttggcttgaatgccgatttctctggactgcgtggccatgcttatct
ccatgcggtaggggtgccgcacggttgcggcaccatgcgcaatcagctgcaacttttcggcagcgcgacaacaattatgc
gttgcgtaaaagtggcagtcaattacagattttctttaacctacgcaatgagctattgcgggggggtgccgcaatgagctgttg
cgtacccccctttttttaagttgttgatttttaagtctttcgcatttcgccctatatctagttctttggtgcccaaagaagggcacccc
tgcggggttcccccacgccttcggcgcggctccccctccggcaaaaagtggcccctccggggcttgttgatcgactgcgc
ggccttcggccttgcccaaggtggcgctgccccccttggaacccccgcactcgccgccgtgaggctcggggggcaggcg
ggcgggcttcgcccttcgactgcccccactcgcataggcttgggtcgttccaggcgcgtcaaggccaagccgctgcgcg
gtcgctgcgcgagccttgacccgccttccacttggtgtccaaccggcaagcgaagcgcgcaggccgcaggccggaggc
ttttccccagagaaaattaaaaaaattgatggggcaaggccgcaggccgcgcagttggagccggtgggtatgtggtcgaa
ggctgggtagccggtgggcaatccctgtggtcaagctcgtgggcaggcgcagcctgtccatcagcttgtccagcagggtt
gtccacgggccgagcgaagcgagccagccggtggccgctcgcggccatcgtccacatatccacgggctggcaaggga
gcgcagcgaccgcgcagggcgaagcccggagagcaagcccgtagggcgccgcagccgccgtaggcggtcacgactt
tgcgaagcaaagtctagtgagtatactcaagcattgagtggcccgccggaggcaccgccttgcgctgcccccgtcgagcc
ggttggacaccaaaagggaggggcaggcatggcggcatacgcgatcatgcgatgcaagaagctggcgaaaatgggca
acgtggcggccagtctcaagcacgcctaccgcgagcgcgagacgcccaacgctgacgccagcaggacgccagagaa
cgagcactgggcggccagcagcaccgatgaagcgatgggccgactgcgcgagttgctgccagagaagcggcgcaag
gacgctgtgttggcggtcgagtacgtcatgacggccagcccggaatggtggaagtcggccagccaagaacagcaggcg
gcgttcttcgagaaggcgcacaagtggctggcggacaagtacggggcggatcgcatcgtgacggccagcatccaccgt
gacgaaaccagcccgcacatgaccgcgttcgtggtgccgctgacgcaggacggcaggctgtcggccaaggagttcatc
ggcaacaaagcgcagatgacccgcgaccagaccacgtttgcggccgctgtggccgatctagggctgcaacggggcatc
gagggcagcaaggcacgtcacacgcgcattcaggcgttctacgaggccctggagcggccaccagtgggccacgtcacc
atcagcccgcaagcggtcgagccacgcgcctatgcaccgcagggattggccgaaaagctgggaatctcaaagcgcgtt
gagacgccggaagccgtggccgaccggctgacaaaagcggttcggcaggggtatgagcctgccctacaggccgccgc
aggagcgcgtgagatgcgcaagaaggccgatcaagcccaagagacggcccgag

pET32a-pglL-CTB4573：

tggcgaatgggacgcgccctgtagcggcgcattaagcgcggcgggtgtggtggttacgcgcagcgtgaccgctacactt
gccagcgccctagcgcccgctcctttcgctttcttcccttcctttctcgccacgttcgccggctttccccgtcaagctctaaatc
gggggctccctttagggttccgatttagtgctttacggcacctcgaccccaaaaaacttgattagggtgatggttcacgtagt
gggccatcgccctgatagacggtttttcgccctttgacgttggagtccacgttctttaatagtggactcttgttccaaactggaa
caacactcaaccctatctcggtctattcttttgatttataagggattttgccgatttcggcctattggttaaaaaatgagctgattta
acaaaaatttaacgcgaattttaacaaaatattaacgtttacaatttcaggtggcacttttcggggaaatgtgcgcggaacccc
tatttgtttatttttctaaatacattcaaatatgtatccgctcatgagacaataaccctgataaatgcttcaataatattgaaaaagg
aagagtatgagtattcaacatttccgtgtcgcccttattcccttttttgcggcattttgccttcctgtttttgctcacccagaaacgc
tggtgaaagtaaaagatgctgaagatcagttgggtgcacgagtgggttacatcgaactggatctcaacagcggtaagatcc
ttgagagttttcgccccgaagaacgttttccaatgatgagcacttttaaagttctgctatgtggcgcggtattatcccgtattgac
gccgggcaagagcaactcggtcgccgcatacactattctcagaatgacttggttgagtactcaccagtcacagaaaagcat
cttacggatggcatgacagtaagagaattatgcagtgctgccataaccatgagtgataacactgcggccaacttacttctgac
aacgatcggaggaccgaaggagctaaccgcttttttgcacaacatgggggatcatgtaactcgccttgatcgttgggaacc
ggagctgaatgaagccataccaaacgacgagcgtgacaccacgatgcctgcagcaatggcaacaacgttgcgcaaacta
ttaactggcgaactacttactctagcttcccggcaacaattaatagactggatggaggcggataaagttgcaggaccacttct
gcgctcggcccttccggctggctggtttattgctgataaatctggagccggtgagcgtgggtctcgcggtatcattgcagca
ctggggccagatggtaagccctcccgtatcgtagttatctacacgacggggagtcaggcaactatggatgaacgaaatag
acagatcgctgagataggtgcctcactgattaagcattggtaactgtcagaccaagtttactcatatatactttagattgatttaa
aacttcatttttaatttaaaaggatctaggtgaagatcctttttgataatctcatgaccaaaatcccttaacgtgagttttcgttcca

ctgagcgtcagaccccgtagaaaagatcaaaggatcttcttgagatcctttttttctgcgcgtaatctgctgcttgcaaacaaa
aaaaccaccgctaccagcggtggtttgtttgccggatcaagagctaccaactctttttccgaaggtaactggcttcagcaga
gcgcagataccaaatactgtccttctagtgtagccgtagttaggccaccacttcaagaactctgtagcaccgcctacatacct
cgctctgctaatcctgttaccagtggctgctgccagtggcgataagtcgtgtcttaccgggttggactcaagacgatagttac
cggataaggcgcagcggtcgggctgaacggggggttcgtgcacacagcccagcttggagcgaacgacctacaccgaa
ctgagatacctacagcgtgagctatgagaaagcgccacgcttcccgaagggagaaaggcggacaggtatccggtaagc
ggcagggtcggaacaggagagcgcacgagggagcttccagggggaaacgcctggtatctttatagtcctgtcgggtttcg
ccacctctgacttgagcgtcgatttttgtgatgctcgtcaggggggcggagcctatggaaaaacgccagcaacgcggcctt
tttacggttcctggccttttgctggccttttgctcacatgttctttcctgcgttatcccctgattctgtggataaccgtattaccgcct
ttgagtgagctgataccgctcgccgcagccgaacgaccgagcgcagcgagtcagtgagcgaggaagcggaagagcgc
ctgatgcggtattttctccttacgcatctgtgcggtatttcacaccgcatatatggtgcactctcagtacaatctgctctgatgcc
gcatagttaagccagtatacactccgctatcgctacgtgactgggtcatggctgcgccccgacacccgccaacacccgctg
acgcgccctgacgggcttgtctgctcccggcatccgcttacagacaagctgtgaccgtctccgggagctgcatgtgtcaga
ggttttcaccgtcatcaccgaaacgcgcgaggcagctgcggtaaagctcatcagcgtggtcgtgaagcgattcacagatgt
ctgcctgttcatccgcgtccagctcgttgagtttctccagaagcgttaatgtctggcttctgataaagcgggccatgttaaggg
cggtttttcctgtttggtcactgatgcctccgtgtaagggggatttctgttcatgggggtaatgataccgatgaaacgagaga
ggatgctcacgatacgggttactgatgatgaacatgcccggttactggaacgttgtgagggtaaacaactggcggtatggat
gcggcgggaccagagaaaaatcactcagggtcaatgccagcgcttcgttaatacagatgtaggtgttccacagggtagcc
agcagcatcctgcgatgcagatccggaacataatggtgcagggcgctgacttccgcgtttccagactttacgaaacacgga
aaccgaagaccattcatgttgttgctcaggtcgcagacgttttgcagcagcagtcgcttcacgttcgctcgcgtatcggtgatt
cattctgctaaccagtaaggcaaccccgccagcctagccgggtcctcaacgacaggagcacgatcatgcgcacccgtgg
ggccgccatgccggcgataatggcctgcttctcgccgaaacgtttggtggcgggaccagtgacgaaggcttgagcgagg
gcgtgcaagattccgaataccgcaagcgacaggccgatcatcgtcgcgctccagcgaaagcggtcctcgccgaaaatga
cccagagcgctgccggcacctgtcctacgagttgcatgataaagaagacagtcataagtgcggcgacgatagtcatgccc
cgcgcccaccggaaggagctgactgggttgaaggctctcaagggcatcggtcgagatcccggtgcctaatgagtgagct
aacttacattaattgcgttgcgctcactgcccgctttccagtcgggaaacctgtcgtgccagctgcattaatgaatcggccaa
cgcgcggggagaggcggtttgcgtattgggcgccagggtggtttttcttttcaccagtgagacgggcaacagctgattgcc
cttcaccgcctggccctgagagagttgcagcaagcggtccacgctggtttgccccagcaggcgaaaatcctgtttgatggt
ggttaacggcgggatataacatgagctgtcttcggtatcgtcgtatcccactaccgagatgtccgcaccaacgcgcagccc
ggactcggtaatggcgcgcattgcgcccagcgccatctgatcgttggcaaccagcatcgcagtgggaacgatgccctcat
tcagcatttgcatggtttgttgaaaaccggacatggcactccagtcgccttcccgttccgctatcggctgaatttgattgcgagt
gagatatttatgccagccagccagacgcagacgcgccgagacagaacttaatgggcccgctaacagcgcgatttgctggt
gacccaatgcgaccagatgctccacgcccagtcgcgtaccgtcttcatgggagaaaataatactgttgatgggtgtctggtc
agagacatcaagaaataacgccggaacattagtgcaggcagcttccacagcaatggcatcctggtcatccagcggatagtt
aatgatcagcccactgacgcgttgcgcgagaagattgtgcaccgccgctttacaggcttcgacgccgcttcgttctaccatc
gacaccaccacgctggcacccagttgatcggcgcgagatttaatcgccgcgacaatttgcgacggcgcgtgcagggcca
gactggaggtggcaacgccaatcagcaacgactgtttgcccgccagttgttgtgccacgcggttgggaatgtaattcagct
ccgccatcgccgcttccactttttcccgcgttttcgcagaaacgtggctggcctggttcaccacgcgggaaacggtctgata
agagacaccggcatactctgcgacatcgtataacgttactggtttcacattcaccaccctgaattgactctcttccgggcgcta
tcatgccataccgcgaaaggttttgcgccattcgatggtgtccgggatctcgacgctctcccttatgcgactcctgcattagg
aagcagcccagtagtaggttgaggccgttgagcaccgccgccgcaaggaatggtgcatgcaaggagatggcgcccaac
agtcccccggccacggggcctgccaccatacccacgccgaaacaagcgctcatgagcccgaagtggcgagcccgatct
tccccatcggtgatgtcggcgatataggcgccagcaaccgcacctgtggcgccggtgatgccggccacgatgcgtccgg
cgtagaggatcgagatctcgatcccgcgaaattaatacgactcactataggggaattgtgagcggataacaattcccctcta

gaactgcataattcgtgtcgctcaaggcgcactcccgttctggataatgttttttgcgccgacatcataacggttctggcaaat
attctgaaatgagctgttgacaattaatcatcggctcgtataatgtgtggaattgtgagcggataacaatttcacacaggaaac
agaattcatgcccgctgaaacgaccgtatccggcgcgcaccccgccgccaaactgccgatttacatcctgccctgcttcctt
tggataggcatcgtcccctttaccttcgcgctcaaactgaaaccgtcgcccgacttttaccacgatgccgccgccgcagccg
gcctgattgtcctgttgttcctcacggcaggaaaaaaactgtttgatgtcaaaatccccgccatcagcttccttctgtttgcaat
ggcggcgttttggtatcttcaggcacgcctgatgaacctgatttaccccggtatgaacgacatcgtctcttggattttcatcttg
ctcgccgtcagcgcgtgggcctgccggagcttggtcgcacacttcggacaagaacgcatcgtgaccctgtttgcctggtcg
ctgcttatcggctccctgcttcaatcctgcatcgtcgtcatccagtttgccggctgggaagacacccctctgtttcaaaacatc
atcgtttacagcgggcaaggcgtaatcggacacatcgggcagcgcaacaacctcggacactacctcatgtggggcatact
cgccgccgcctacctcaacggacaacgaaaaatccccgccgccctcggcgtaatctgcctgattatgcagaccgccgtttt
aggtttggtcaactcgcgcaccatcttgacctacatagccgccatcgccctcatccttcccttctggtatttccgttcggacaaa
tccaacaggcggacgatgctcggcatagccgcagccgtattccttaccgcgctgttccaattttccatgaacaccattctgga
aacctttactggcatccgctacgaaactgccgtcgaacgcgtcgccaacggcggtttcacagacttgccgcgccaaatcga
atggaataaagcccttgccgccttccagtccgccccgatattcgggcacggctggaacagttttgcccaacaaaccttcctc
atcaatgccgaacagcacaacatatacgacaacctcctcagcaacttgttcacccattcccacaacatcgtcctccaactcct
tgcagagatgggaatcagcggcacgcttctggttgccgcaaccctgctgacgggcattgccgggctgcttaaacgccccc
tgacccccgcatcgcttttcctaatctgcacgcttgccgtcagtatgtgccacagtatgctcgaatatcctttgtggtatgtctatt
tcctcatccctttcggactgatgctcttcctgtcccccgcagaggcttcagacggcatcgccttcaaaaaagccgccaatctc
ggcatactgaccgcctccgccgccatattcgcaggattgctgcacttggactggacatacacccggctggttaacgcctttt
cccccgccactgacgacagtgccaaaaccctcaaccggaaaatcaacgagttgcgctatatttccgcaaacagtccgatg
ctgtccttttatgccgacttctccctcgtaaacttcgccctgccggaataccccgaaacccagacttgggcggaagaagcaa
ccctcaaatcactaaaataccgcccccactccgccacctaccgcatcgccctctacctgatgcggcaaggcaaagttgcag
aagcaaaacaatggatgcgggcgacacagtcctattacccctacctgatgccccgatacgccgacgaaatccgcaaactg
cccgtatgggcgccgctgctacccgaactgctcaaagactgcaaagccttcgccgccgcgcccggtcatccggaagcaa
aaccctgcaaatgaaagcttggctgttttggcggatgagagaagattttcagcctgatacagattaaatcagaacgcagaag
cggtctgataaaacagaatttgcctggcggcagtagcgcggtggtcccacctgaccccatgccgaactcagaagtgaaac
gccgtagcgccgatggtagtgtggggtctccccatgcgagagtagggaactgccaggcatcaaataaaacgaaaggctc
agtcgaaagactgggcctttcgttttatctgttgtttgtcggtgaacgctctcctgagtaggacaaatccgccgggagcggatt
tgaacgttgcgaagcaacggcccggagggtggcgggcaggacgcccgccataaactgccaggcatcaaattaagcaga
aggccatcctgacggatggcctttttgcgtttctacaaactcttttgtttatttttctaaatacattcaaatatgtatccgctcatgag
acgagctctgcataattcgtgtcgctcaaggcgcactcccgttctggataatgttttttgcgccgacatcataacggttctggc
aaatattctgaaatgagctgttgacaattaatcatcggctcgtataatgtgtggaattgtgagcggataacaatttcacacagg
aaacagaattcatgaagaaaatttggctggccttagccggcctggttctggcattcagcgccagcgcaaccccgcagaaca
tcaccgacctgtgcgccgagtaccacaacacccaaatttataccctgaacgacaaaatttttagctacaccgagagcctggc
aggcaagcgcgagatggccatcatcaccttcaagaacggcgccattttccaggtggaggtgccgggcagccagcacatc
gacagtcagaagaaggccatcgagcgcatgaaggacaccctgcgcatcgcctacctgaccgaggccaaggtggagaa
gctgtgcgtgtggaacaacaagaccccgcacgccatcgccgcaatcagcatggccaacggcggatccggctccagcgc
cgtgaccgagtactatctgaaccatggcgagtggccgggtaataacaccagcgccggcgtggccacaagcagtgagatc
aagctcgagcaccaccaccaccaccactgagatccggctgctaacaaagcccgaaaggaagctgagttggctgctgcca
ccgctgagcaataactagcataaccccttggggcctctaaacgggtcttgaggggttttttgctgaaaggaggaactatatcc
ggat
